# Banana bunchy top virus genetic diversity in Pakistan and association of diversity with recombination in its genomes

**DOI:** 10.1371/journal.pone.0263875

**Published:** 2022-03-07

**Authors:** Sana Bashir, Syed Muhammad Saqlan Naqvi, Aish Muhammad, Iqbal Hussain, Kazim Ali, Muhammad Ramzan Khan, Sumaira Farrakh, Tayyaba Yasmin, Muhammad Zeeshan Hyder

**Affiliations:** 1 Department of Biosciences, COMSATS University Islamabad, Islamabad, Pakistan; 2 Department of Biochemistry, PMAS-Arid Agriculture University, Rawalpindi, Pakistan; 3 National Institute for Genomics and Advanced Biotechnology, National Agriculture Research Centre, Islamabad, Pakistan; Deen Dayal Upadhyaya Gorakhpur University, INDIA

## Abstract

Banana Bunchy top virus (BBTV) is a multipartite circular single strand DNA virus that belongs to genus *Babuvirus* and family *Nanoviridae*. It causes significant crop losses worldwide and also in Pakistan. BBTV is present in Pakistan since 1988 however, till now only few (about twenty only) sequence of genomic components have been reported from the country. To have insights into current genetic diversity in Pakistan fifty-seven genomic components including five complete genomes (comprises of DNA-R, -U3, -S, -M, -C and -N components) were sequenced in this study. The genetic diversity analysis of populations from Pakistan showed that DNA-R is highly conserved followed by DNA-N, whereas DNA-U3 is highly diverse with the most diverse Common Region Stem-loop (CR-SL) in BBTV genome, a functional region, which previously been reported to have undergone recombination in Pakistani population. A Maximum Likelihood (ML) phylogenetic analysis of entire genomes of isolates by using sequence of all the components concatenated together with the reported genomes around the world revealed deeper insights about the origin of the disease in Pakistan. A comparison of the genetic diversity of Pakistani and entire BBTV populations around the world indicates that there exists a correlation between genetic diversity and recombination. Population genetics analysis indicated that the degree of selection pressure differs depending on the area and genomic component. A detailed analysis of recombination across various components and functional regions suggested that recombination is closely associated with the functional parts of BBTV genome showing high genetic diversity. Both genetic diversity and recombination analyses suggest that the CR-SL is a recombination hotspot in all BBTV genomes and among the six components DNA-U3 is the only recombined component that has extensively undergone inter and intragenomic recombination. Diversity analysis of recombinant regions results on average one and half fold increase and, in some cases up to four-fold increase due to recombination. These results suggest that recombination is significantly contributing to the genetic diversity of BBTV populations around the world.

## Introduction

Banana bunchy top disease (BBTD) is the most common and devastating viral disease of Banana, predominantly found in Pacific and Asian regions. It has been considered one of the most important plant viral diseases around the world [1]. In Pakistan, BBTD was first observed in 1988 in Sindh province. Later, based on symptomology it was identified in 1991 [2]. BBTD is caused by Banana Bunchy top Virus (BBTV), which belongs to genus *Babuvirus* in the family *Nanoviridae* [3]. BBTV is transmitted persistently by the aphid *Pentalonia nigronervosa* Coq, its sole known vector, and infects only the members of *Musaceae* [4].

Banana bunchy top virus (BBTV) is a multipartite circular single stranded (css) DNA virus comprising of six css DNA components each of about 1Kb in size [5, 6]. BBTV genome consists of six integral ssDNA components, including DNA-R, C, M, S and -N which encode master replication initiation protein (M-Rep), cell cycle link protein (Clink) movement protein (MP), capsid protein (CP) and nuclear-shuttle protein (NSP), respectively while DNA-U3 encodes a protein for which no function has been assigned so far [5, 7–10]. Two conserved notable functional regions exist in all six DNA components of BBTV, which include the common region stem-loop (CR-SL), which is about 70 nucleotides long and contains a 31 nucleotides origin of replication (ori) for proposed rolling-circle replication [11, 12]. Second region which is more than 70% identical in six components, and about 90 nucleotide long, is the common region major (CR-M) [5, 13] to which small ssDNA primers bound and initiate complementary-strand DNA synthesis after entering a host cell [14] and is under the course of concerted type evolution [15, 16].

Occurrence of genetic variations due to recombination is a well-established phenomenon in BBTV [17]. This phenomenon is of particular significance in the evolution of viruses as it provides the possibility of natural recombination events, which leads to extensive viral diversity [17]. In geminivirus evolution, the importance of recombination is well recognized [18–20] as it is the most probable mechanism responsible for the genetic diversification of agriculturally important begomovirus species [21, 22]. Whilst generating the descendants with increased fitness, recombination is also a source for increased genetic diversity in begomoviruses [17]. In viruses, recombination breakpoints identification is a useful way to detect circulating recombinant forms and to infer the underlying recombination mechanisms such as intra and intergenomic recombination [23, 24]. Although sequence analysis of individual BBTV components [9, 10, 15, 16, 25–30] and complete BBTV genomes [31–36] revealed evidence of intra and intergenomic recombination, but no study related to the understanding the contribution of these recombination mechanisms in genetic diversity has been reported so far for BBTV. Previous molecular analysis of BBTV from Pakistan has been very limited with only one partial genome [37] and a few DNA components [16, 25, 37] have been sequenced from different districts of Sindh province. Thus, earlier phylogenetic analyses within the country performed on BBTV have used sequences of individual BBTV components rather than full genomes.

In the current study, we reported the sequence of complete genomes of five BBTV isolates originated from different districts of Sindh, Pakistan. The diversity analysis and putative recombination events were studied for Pakistani isolates and then for sequences available in the public database in GenBank. Also, the contribution of genetic diversity by recombination, recombination hotspots and population genetics are studied for BBTV genomes to better understand the evolution of this virus in various geographic regions around the globe.

## Material and methods

### DNA extraction, PCR amplification, cloning and sequencing

BBTV infected plant material showing typical BBTD symptom was collected from district Tandojam, Sindh province in 2006 for P.TJ1 isolate (for which DNA-R was previously reported by Hyder and colleagues (2007)), and in 2007, for P.BS1 & 2, P.GH1 & 2, P.HD1 & 2, P. JS1 (the DNA-U3 for P.GH1, P.JS1 and P.HD1 were previously reported by Hyder and colleagues (2011)), P.KP1 & 2, P.MT1 & 2, P.NS1, P.TA1 & 2 from Tandojam, Bhitshah, Ghotki, Hyderabad, Jamshoro, Khairpur, Matiari, Nawabshah and Tandoadam districts of the Sindh respectively. The P.TJ3, P.NARC and P.Sakrand & P.TJ4 were isolated during 2011, 2017 and 2018 respectively from Tandojam district. The total genomic DNA extraction was performed using the CTAB method as described by Hyder et al., (2007). PCR amplification and DNA sequencing were performed with two pairs of adjacent outwardly extending abutting primer each specific for a different location in each genomic component. Using this technique multiple reads in sequencing every component was generated and the sites where one set of primer binds, were sequenced using 2nd pair of primer and vice versa. for each genomic component of BBTV (Table 1).

**Table 1:** Details of primers used to sequence BBTV genomic components from Pakistan.

BBTV Genomic Component	Set	Name of Primer	Sequence 5’- 3’direction	Nucleotides Coordinate (nt)
DNA-R	Set A	DNA-R AF	ATGGCGCGATATGTGGTATG	102–121
		DNA-R AR	TCTGTCGTCGATGATGATCTTG	102–80
	Set B	DNA-R BF	CCAAATGGAGGAGAAGGAAAG	642–662
		DNA-R BR	GCCATAGACCCAAATTATTCTCCG	641–618
DNA-U3	Set A	DNA-U3 AF	TTGTGCTGAGGCGGAAGAT	313–331
		DNA-U3 AR	CCACCTTCACAGAAGAGAG	312–294
	Set B	DNA-U3 BF	CAGATTAATTCCTTAGCGAC	837–856
		DNA-U3 BR	GACCGTTCATTCAACTTGAC	836–817
DNA-S	Set A	DNA-S AF	GTATCCGAAGAAATCCATC	236–254
		DNA-S AR	CTAGCCATTTGTTGTCTG	235–218
	Set B	DNA-S BF	GGAAGAATGTAACGGAGGTCG	643–663
		DNA-S BR	TCAACACGGTTGTCTTCCTCA	642–622
DNA-M	Set A	DNA-M AF	ATGGCATTAACAACAGAGCG	282–301
		DNA-M AR	TTAGCAGGGTCCTATTTATAGG	281–260
	Set B	DNA-M BF	GGATGATCAAGGAAGACG	593–610
		DNA-M BR	CTTCTATTTGGTTGAGAAGG	592–573
DNA-C	Set A	DNA-C AF	GAATCGTCTGCTATGCCTG	252–270
		DNA-C AR	CCAGAACTCCATTTCTCTTC	251–232
	Set B	DNA-C BF	GTTCTCTCTTCTTCATCG	585–602
		DNA-C BR	CTCATCACAATAGAGATCTTG	584–564
DNA-N	Set A	DNA-N AF	GATGGATTGGGCGGAATCA	276–284
		DNA-N AR	GCTTCTGCTTTGCTTTCGC	275–257
	Set B	DNA-N BF	GAGCAGAGACATGGAAGTTAG	642–662
		DNA-N BR	CAATCTATTCCTGGCGCAAC	641–622
M13 Universal Primers	-	M13 F	TGTAAAACGACGGCCAGT	-
		M13 R	CAGGAAACAGCTATGACC	-

Note: The nucleotide coordinates are according to the P.TJ1 isolate genomic components’ sequences.

The PCR amplifications were carried out either using GoTaq® PCR Core System I (Promega Corp. Madison, WI, USA) or using Taq DNA Polymerase (recombinant) (Fermentas UAB Lithuania), according to manufacturer’s instructions. A typical PCR reaction contained about 50 ng DNA template, respective Taq buffer, 1.5 mM MgCl2, 200 μM of each dNTPs, 2.5 units of Taq DNA polymerase and 50 pM of each primer. The thermal profile for both primer sets included pre-PCR denaturation at 94°C for 3 minutes followed by 35 cycles of denaturing at 94°C for 30 seconds, annealing at 52°C for 30 seconds and extension at 72°C for 45 seconds, and a final extension at 72°C for 20 minutes. The PCR products of P.TJ1 were ligated into pTZ57R (InsTA Cloning Kit, Fermentas UAB Lithuania) according to the manufacturer’s instructions and transformed into Escherichia coli DH5α (supE44, ΔlacU169 (Φ80lacZΔM15), hsdR17, recA1, endA1, gyrA96, thi-1, relA1) cells by electroporation. While for those isolates sampled during 2007, the PCR products were ligated directly into the pGEM®-T Easy Vector (Promega, Madison, WI) and cloned in E. coli DH5α cell. The clones were selected using 50 μg/ml ampicillin and screened for white colonies generated by insertional inactivation of functional beta galactosidase gene whose expression was induced by IPTG (Isopropyl β- d-1-thiogalactopyranoside) converting chromogenic X-GAL (5-bromo-4-chloro-3-indolyl-β-D-galactopyranoside) into blue color in non-recombinant bacterial cells. The plasmid DNA was extracted using a minipreparation protocol according to Sambrook and Russell [38], and confirmed by digestion with appropriate restriction endonucleases. For every component of an isolate, both the strands of two individual clones originated from two different primer sets of the respective component were sequenced. The genomic components of P.TJ1 isolate was sequenced using the CEQ Dideoxy Dye Terminator cycle sequencing Kit (Beckman Coulter, USA) and the CEQ 8000 Genetic Analysis System (Beckman Coulter, USA) by universal M13 and insert-specific primers to obtain full length sequence of each strand. The isolates sampled during 2007 were processed in a similar way and sequenced at sequencing facility of Iowa State University, U.S.A. Both the strands of the PCR products of two different primer sets specific for the same genomic component, from each isolate, sampled in 2011, 2017 and 2018 were directly sequenced using commercial DNA Sequencing Facility of Macrogen (Macrogen, Inc. South Korea). The sequence data of both the strand of a clone/PCR product was assembled first using DNA Dragon Contig Sequence Assembly Software (version 1.5.0) SequentiX—Digital DNA Processing (Germany) (https://www.sequentix.de/software_dnadragon.php). Then the consensus of sequencing, originating from two different primer sets, was developed for each component of an isolate. The consensus sequence of each sequenced component from an isolate was submitted in GenBank. The accession numbers were (MK140625, MK140626, MK140627, FJ859727, FJ859728, FJ859722, FJ859733, FJ859734, FJ859732, FJ859723, FJ859724, FJ859729, FJ859730, FJ859731, FJ859725, FJ859726, JX170762 for DNA-R), (MK140628, MK140629, MK140630, JX1700764 for DNA-U3), (MK140619, MK140620, MK140621, EF593169, FJ859740, FJ859741, FJ859735, FJ859746, FJ859747, FJ859745, FJ859736, FJ859737, FJ859742, FJ859743, FJ859744, FJ859738, FJ859739, JX170763 for DNA-S), (MK140616, MK140617, MK140618, EU095948, JX467685, JX467686, JX46768, JX170760 for DNA-M), (MK140613, MK140614, MK140615, EF520722, JX170759 for DNA-C), (MK140624, MK140625, MK140626, EF529519, JX170761 for DNA-N) respectively.

### Sequence retrieval and genetic diversity

The full-length sequences of all components of BBTV genome were obtained from NCBI GenBank using Taxonomy Browser till April 05, 2020 and given abbreviated names for convenience (S1 Table). The coding and regulatory regions, including CR-SL and CR-M were identified as described by Burns and colleagues (1995), and the sequences were started from the start of major ORF identified by Herrera-Valencia and colleagues (2007) [39] in each component for alignment through MAFFT version 6.864 [40]. The genetic diversity values of each BBTV component and subgenomic regions were determined on the aligned sequences first for Pakistani isolates and then worldwide. The nucleotide diversity range analysis was performed using MatGAT [41], while Pair-wise average nucleotide diversity per 100 sites π (Pi) and Watterson estimator θw (Theta-w) for population mutation rates per 100 sites were determined using DnaSP version 6.12.03 [42].

To study the relationship BBTV isolates of Pakistan with BBTV isolates from other parts of the world, a Maximum Likelihood (ML) phylogenetic genomic tree based on the concatenated nucleotide sequences of entire genomic components (i.e. DNA-R, U3, S, M, C and N concatenated together in single sequence for an isolate) of those isolates for which full genomic sequences were available in the GenBank, was constructed with 1000 bootstrap replicates. In addition, individual component-based ML phylogenetic analyses of full-length sequences of each component separately was also performed in the Molecular Evolutionary Genetics Analysis program (MEGA), version X [43] with 1000 bootstrap replicates. The phylogenetic trees were visualized using FigTree version 1.4.3 [44] (http://tree.bio.ed.ac.uk/software/).

### Selection pressure analysis

The selection pressure was estimated by d_N_/d_S_ ratio, where d_N_ represents the average number of nonsynonymous substitutions per nonsynonymous site and d_S_ is the average number of synonymous substitutions per synonymous site. In MEGA X [43]. d_N_ and d_S_ values were estimated separately by using Nei-Gojobori method (Jukes-Cantor). The gene is under neutral selection when dN/dS ratio = 1, positive (or diversifying) selection when the dN/dS ratio is > 1 and negative (or purifying) selection when dN/dS ratio < 1.

### Neutrality test

DnaSP version 6.12.03 [42] was used for testing Tajima D [45], Fu and Li’s D*and F* [46] number of haplotypes (H), haplotype diversity (Hd) and nucleotide diversity (π). Tajima’s D test in a genomic region measures the departure from neutrality for all mutations. Tajima’s D test is based on the differences between the number of segregating sites and the average number of nucleotide differences. Fu and Li’s D*test is based on the differences between the total number of mutations and number of singletons (mutations appearing only once among the sequence). Fu and Li’s F*test is based on the differences between the number of singletons and the average number of nucleotide differences between every pair of sequences. Haplotype diversity refers to the frequency and number of haplotypes in the population while nucleotide diversity estimates the average pairwise differences among sequences.

### Recombination analysis

The recombination analysis was performed using Recombination Detection Program (RDP) version 4 Beta14 [47]. The intergenomic (intracomponent) recombination was determined by using MAFFT aligned sequences separately for each component, while intragenomic (intercomponent) recombination was determined using MAFFT aligned sequences of all the component together. The recombination analysis was performed using maximum χ2 (MaxChi) [48], bootscan [49], GENECONV [50], LARD [51], Distance Plot (SimPlot) [52], sister scanning (SiSscan) [53], TOPAL [54], chimaera [55] and reticulate (compatibility matrix) [56] implemented in RDP with the default values (P. value = 0.05, Multiple Comparison Correction = Bonferroni Correction, Number of permutations = 0) except that sequence type was set to circular.

The contribution of genetic diversity by recombinants in their respective populations was determined by selecting the recombined genomic regions for each recombination event in alignments of the respective component as identified by RDP and then selecting recombination population in the ML trees from the respective geographic location, aligning only the recombinant regions using MAFFT software and then calculating the genetic diversity range, π (Pi) and Watterson estimator θw (Theta-w) values as described earlier for the respective recombinant population with and without recombinant sequences.

## Results

### Genetic diversity of BBTV population

Total fifty-seven genomic components including five complete genomes of BBTV were sequenced from Pakistan during 2006 to 2018 (accession numbers given in Table 2). The genetic diversity analysis of these components along with other reported isolates of BBTV from Pakistan [16, 25, 37], showed that DNA-R is highly conserved followed by DNA-N, whereas DNA-U3 is highly diverse followed by DNA-C. Similarly, for coding regions, DNA-R is highly conserved followed by DNA-M, while, DNA-C followed by DNA-S are highly diverse (Table 3). However, when different functional regions of BBTV genome such as CR-M and CR-SL are compared with the full-length sequences and coding regions, it is revealed that between BBTV components CR-M is highly conserved while CR-SL is highly diverse in DNA-U3. Contrary to sequences of full-length components and coding regions, CR-M in DNA-R is highly diverse followed by DNA-C. In a similar contradiction, DNA-U3 bears the most conserved CR-M region. The sequence diversity in the CR-SL region of Pakistani BBTV population reveals very interesting findings, the DNA-R, -S, -M and -C have identical CR-SL and there exists no divergence, however, DNA-U3 and N showed diverse CR-SL region. While in DNA-N the genetic diversity was lower than DNA-U3 in this region (Table 3).

**Table 2:** Description of Banana bunchy top virus isolates sequenced from Pakistan for this study.

Isolate	Geographical origin			Accession Numbers						Isolation	
Abbreviation	Region	Country	Subgroup	DNA-R	DNA-U3	DNA-S	DNA-M	DNA-C	DNA-N	Year	Reference
P.TJ1	Tandojam	Pakistan	PIO	-	-	EF593169	EU095948	EF520722	EF529519	2005	This study
P.BS1	Bhitshah	Pakistan	PIO	FJ859727	-	FJ859740	JX467685	-	-	2007	This study
P.BS2	Bhitshah	Pakistan	PIO	FJ859728	-	FJ859741	-	-	-	2007	This study
P.GH1	Ghotki	Pakistan	PIO	FJ859722	-	FJ859735	-	-	-	2007	This study
P.HD1	Hyderabad	Pakistan	PIO	FJ859733	-	FJ859746	JX467686	-	-	2007	This study
P.HD2	Hyderabad	Pakistan	PIO	FJ859734	-	FJ859747	-	-	-	2007	This study
P.JS1	Jamshoro	Pakistan	PIO	FJ859732	-	FJ859745	-	-	-	2007	This study
P.KP1	Khairpur	Pakistan	PIO	FJ859723	-	FJ859736	-	-	-	2007	This study
P.KP2	Khairpur	Pakistan	PIO	FJ859724	-	FJ859737	-	-	-	2007	This study
P.MT1	Matiari	Pakistan	PIO	FJ859729	-	FJ859742	-	-	-	2007	This study
P.MT2	Matiari	Pakistan	PIO	FJ859730	-	FJ859743	-	-	-	2007	This study
P.NS1	Nawabshah	Pakistan	PIO	FJ859731	-	FJ859744	JX467687-	-	-	2007	This study
P.TA1	Tandoadam	Pakistan	PIO	FJ859725	-	FJ859738	-	-	-	2007	This study
P.TA2	Tandoadam	Pakistan	PIO	FJ859726	-	FJ859739	-	-	-	2007	This study
P.TJ3	Tandojam	Pakistan	PIO	JX170762	JX170764	JX170763	JX170760	JX170759	JX170761	2011	This study
P.NARC	Tandojam	Pakistan	PIO	MK140625	MK140628	MK140619	MK140616	MK140613	MK140622	2017	This study
P.Sakrand	Tandojam	Pakistan	PIO	MK140627	MK140630	MK140621	MK140618	MK140615	MK140624	2018	This study
P.TJ4	Tandojam	Pakistan	PIO	MK140626	MK140629	MK140620	MK140617	MK140614	MK140623	2018	This study

**Note:** *For P.TJ1, the DNA-R (accession # AY996562) had been previously reported by Hyder et al., (2007) and DNA-U3 (accession#GQ214699) was reported by Hyder et al., 2011 -and by reporting rest of the components, its genome is completely reported in this stuy. Similarly,DNA-U3 of P.GH1. P. HD1 and P.JS1 (and their respective accession numbers FJ859748, FJ859750 and FJ859749) had been reported earlier by Hyder et al., (2011) and rest are being reported in this study. The isolates sampled in 2006 and 2007 are related to Ph.D thesis (Hyder, 2009) and not reported earlier elsewhere. In total 57 components belonging to 18 isolates from different geograhpic locations are sequenced for this study.

**Table 3:** Genetic diversity of Banana bunchy top virus population in Pakistan.

Components	Population	Full-length			Coding region			CR-M			CR-SL		
		Percent Identity Range	π	θw	Percent Identity Range	π	θw	Percent Identity Range	π	θw	Percent Identity Range	π	θw
**DNA-R**	Total	99.1-100-	0.26± 0.04	0.48± 018	99.5–100	0.13 ± 0.03	0.31 ± 0.13	95.8–100	2.41 ± 0.24	2.40 ± 1.15	100	0.00	0.00
**DNA-U3**	Total	98.4–99.9	0.69 ± 0.15	0.90 ±0.40	98.3–100	0.45± 0.15	0.62 ± 0.38	99.0–100	0.24± 0.18	0.40 ± 0.40	90.2–100	3.18 ± 1.37	3.24 ± 1.40
**DNA-S**	Total	99.1–100	0.48± 0.10	0.99 ±0.36	97.7–100	0.51 ± 0.17	0.88 ± 0.35	97.0–100	0.40 ± 0.21	1.23 ± 0.70	100	0.00	0.00
**DNA-M**	Total	98.5–100	0.33 ± 0.14	0.49 ± 0.23	99.4–100	0.18 ± 0.07	0.31 ± 0.20	99.0–100	0.63 ± 0.10	0.42 ±0.42	100	0.00	0.00
**DNA-C**	Total	98.6–99.8	0.67 ± 0.15	0.80 ± 0.39	97.4–100	0.58 ± 0.13	0.67 ± 0.36	95.0–100	1.46 ± 0.50	1.79 ± 1.13	100	0.00	0.00
**DNA-N**	Total	98.4–99.8	0.31 ± 0.06	0.36 ±0.20	97.4–100	0.25 ± 0.11	0.28 ± 0.19	99.0–100	0.36± 0.23	0.48 ± 0.48	98.8–100	0.55 ± 0.35	0.73 ± 0.73

**Note**: Percent identity range analysis indicates the minimum to maximum percent nucleotide identity values obtained after pair-wise comparison of isolates in the entire population. π (Pi) denotes pair-wise average nucleotide diversity per 100 sites along with standard deviation, while θw (Theta-w) signifies Watterson estimator for population mutation rates per 100 sites with its standard deviation. The population *n* for each components and subgroups are as follows: DNA-R [*n*_(total)_ = 24], DNA-U3 [*n*_(total)_ = 9], DNA-S [*n*_(total)_ = 22], DNA-M [*n*_(total)_ = 9], D*N*A-C [*n*_(total)_ = 7] and DNA-N [*n*_(total)_ = 6] where ‘total’ means all the sequence of isolates of particular genomic component.

The BBTV population in Pakistan is considered to have a monophyletic origin and supposed to have been originated from a single introduction of BBTV in the country [25, 37] that took place before 1988 when BBTV was first observed in the Sindh [2]. The current diversity analysis (Table 3) suggests that BBTV components in Pakistan, are not evolving at a similar rate as some components are quite conserved i.e. DNA-R and DNA-N, while some have more diversification (i.e. DNA-U3). Interestingly, within a component, the divergence is not uniform, the most conserved component i.e. DNA-R has the most diverse CR-M, while DNA-U3 has CR-SL that is the most diverse functional region in the entire genome of BBTV.

Once having these insights about the genetic diversity of BBTV population in Pakistan, the genetic diversity of entire BBTV populations around the world was also calculated in similar way. The full-length sequences (1425 full-length components) from various BBTV isolates were obtained from GenBank using Taxonomy browser (S1 Table) and their diversity was calculated for two major groups of BBTV population i.e., the South Pacific and Asian groups [26] now referred to as Pacific Indian Ocean (PIO) and the Southeast Asian (SEA) group respectively [57] along with the entire world population. The analysis (Table 4) revealed that PIO group is more conserved compared to the SEA group, a finding that is consistent with the previous studies [7, 9, 26, 33, 58]. Among components DNA-R is the most conserved, while DNA-U3 is the most diverse component (Table 4) and the coding region analysis also follows the same trend. However, contradictory to the diversity analysis of BBTV-Pakistan, DNA-U3 has the most diverse CR-M in the entire BBTV population, while DNA-C has the most conserved CR-M region. The DNA-N has the most conserved CR-SL region in the entire population, while, like Pakistani population, DNA-U3 also has the most diverse CR-SL region. These analyses clearly suggest that different components and their functional part in BBTV genome are harboring different extent of genetic diversity and this diversity is related to different broader geographic regions such as PIO and SEA or to a country such as Pakistan.

**Table 4:** Genetic diversity of Banana bunchy top virus population in the world.

Components	Population	Full-length			Coding region			CR-M			CR-SL		
		Percent Identity Range	π	θw	Percent Identity Range	π	θw	Percent Identity Range	π	θw	Percent Identity Range	π	θw
**DNA-R**	Total	86.6–100	4.46± 0.17	6.53±1.31	90.2–100	3.95±0.14	5.87 ± 1.19	59.3–100	9.40± 0.40	11.49±2.84	70.8–100	1.21± 0.15	7.54± 2.01
	PIO	92–100	2.18± 0.76	5.28±1.11	94.6–100	2.00±0.06	4.70±1.00	74.6–100	4.65± 0.27	10.84±2.77	82.2–100	1.39± 0.16	5.89±1.66
	SEA	89.8–100	2.81± 0.30	4.50±1.16	91.9–100	2.65±0.29	4.00+1.04	81.0–100	4.60± 0.96	5.52±3.04	71.4–100	0.92± 0.42	5.39±1.88
**DNA-U3**	Total	66.4–99.9	8.45±0.46	11.11±2.41	67.9–100	7.83±0.49	11.58 ± 2.6	43.0–100	13.38±0.81	14.59±3.66	38.2–100	8.41± 0.47	12.08±3.60
	PIO	70.9–100	5.99±0.33	9.53± 2.14	75.3–100	4.97± 0.41	10.78± 2.55	47.3–100	8.70± 0.66	13.60±3.57	54.1–100	7.58± 0.32	6.57± 2.16
	SEA	67.6–99.7	6.41± 0.82	8.38± 2.74	75.8–100	6.19 ± 0.79	8.14± 2.52	66.3–100	7.57±1.66	11.20±3.66	31.4–100	10.66± 2.35	14.42±5.19
**DNA-S**	Total	79.9–99.6	5.41± 0.26	8.37 ±1.73	86.9–99.6	4.01 ± 0.15	6.79 ± 1.44	60.4–100	13.37 ± 0.85	10.86 ± 2.78	75.6–100	2.22 ± 0.21	7.08 ± 1.99
	PIO	87.2–100	2.59 ± 0.16	7.41 ± 1.59	87.5–100	2.44 ± 0.11	6.28 ± 1.39	79.2–100	3.05± 0.29	10.82 ± 2.71	81.7–100	2.25 ± 0.21	6.50 ± 1.92
	SEA	84.7–100	4.58 ± 0.64	6.24 ± 1.68	91.3–100	2.82 ± 0.32	3.81 ± 1.07	61.4–100	6.17± 2.13	9.80 ± 3.13	76.8–100	2.10 ± 0.67	3.90 ± 1.52
**DNA-M**	Total	72.2–100	8.20± 0.41	9.04± 1.96	80.7–100	6.73± 0.25	9.05± 2.02	63.0–100	10.83± 0.91	8.67± 2.24	72.4–100	2.26± 0.39	5.45±1.70
	PIO	85.2–100	4.34± 0.10	7.30± 1.65	82.6–100	4.55± 0.14	7.99±1.86	85.9–100	2.4 ± 0.15	6.13± 1.72	71.1–100	1.58± 0.21	4.66±1.48
	SEA	79.4–100	5.44± 0.79	7.87± 2.27	89.5–100	4.04± 0.45	5.39± 1.64	62.4–100	1.72± 0.32	2.41± 1.03	79.3–100	0.85± 0.40	4.46±1.13
**DNA-C**	Total	83.6–100	4.80± 0.38	7.43 ± 1.63	83–100	4.71 ± 0.36	7.91 ± 1.77	67.3–100	7.94 ± 0.83	7.86 ± 2.13	80.4–100	2.87 ± 0.36	5.07 ± 1.64
	PIO	92.4–100	1.58± 0.08	5.69 ± 1.30	90.1–100	1.66 ± 0.12	6.44 ± 1.50	92.1–100	0.64 ± 0.10	4.22 ± 1.33	79.3–100	2.41 ± 0.23	4.29 ± 1.44
	SEA	88–100	4.04± 0.60	5.51 ± 1.63	88.9–100	3.46 ± 0.60	5.09 ± 1.55	47.5–100	3.58 ± 0.44	3.83 ± 1.48	72–100	6.35 ± 1.27	7.71 ± 2.81
**DNA-N**	Total	76.2–99.4	7.25 ± 0.39	7.26 ±1.58	87.3–99.8	4.72 ± 0.30	6.04 ± 1.38	66.3–100	8.88± 0.89	10.37 ± 2.68	95.1–100	1.11 ± 0.10	4.11 ± 1.38
	PIO	76.6–100	3.83 ± 0.21	6.66 ± 1.52	78.6–100	2.10 ± 0.16	5.56 ± 1.33	94–100	2.65 ± 0.67	9.22 ± 2.48	90.2–100	0.92 ± 0.09	2.76 ± 1.08
	SEA	84.6–100	5.86 ± 0.86	5.90 ± 1.69	89.7–100	4.44± 0.54	4.20± 1.25	63.4–100	4.31 ± 0.51	3.32± 1.33	96.3–100	1.41 ± 0.26	2.32 ± 1.12

**Note**: Percent identity range analysis indicates the minimum to maximum percent nucleotide identity values obtained after pair-wise comparison of isolates in a given population. π (Pi) denotes pair-wise average nucleotide diversity per 100 sites along with standard deviation, while θw (Theta-w) signifies Watterson estimator for population mutation rates per 100 sites with its standard deviation. The population *n* for each components and subgroups are as follows: DNA-R [*n*_(total)_ = 342 {*n*_(PIO)_ = 263, *n*_(SEA)_ = 79}], DNA-U3 [*n*_(total)_ = 200 {*n*_(PIO)_ = 162, *n*_(SEA)_ = 38}], DNA-S [*n*_(total)_ = 274 {*n*_(PIO)_ = 214, *n*_(SEA)_ = 60}], DNA-M [*n*_(total)_ = 205, {*n*_(PIO)_ = 164, *n*_(SEA)_ = 41}], D*N*A-C [*n*_(total)_ = 194 {*n*_(PIO)_ = 156, *n*(_SEA_) = 38}] and DNA-N [*n*_(total)_ = 198 {*n*_(PIO)_ = 154, *n*_(SEA)_ = 44}] where ‘total’ means all the sequence of isolates of particular genomic component.

### Phylogenetic analysis

**Fig 1:**
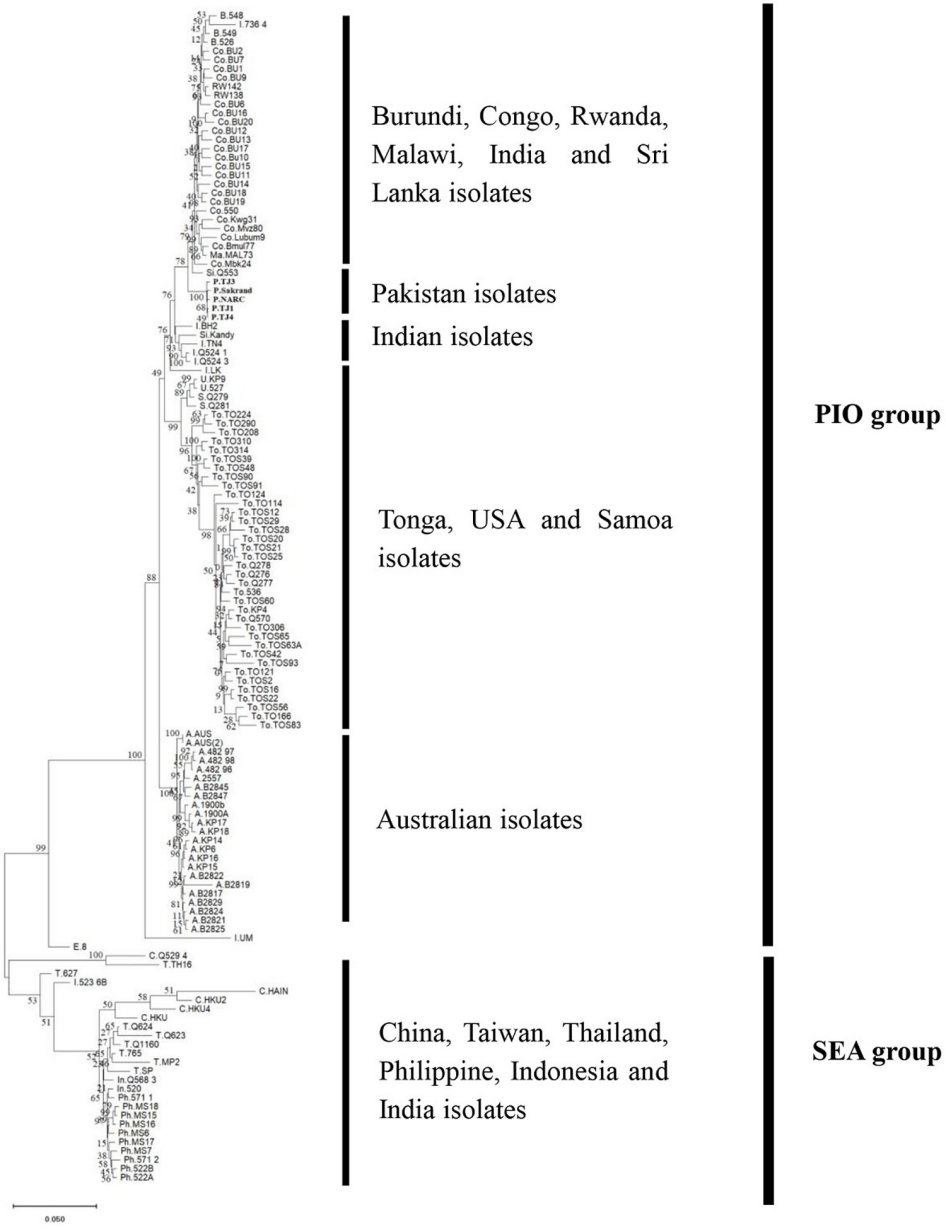
Maximum Likelihood phylogenetic analysis of Banana bunchy top virus entire genomes, showing the relationship of newly characterized BBTV genomes from Pakistan. Phylogenetic Maximum Likelihood analysis based on the concatenated nucleotide sequences of BBTV genomic components (R, U3, S, M, C, N together) indicates the evolutionary analyses of Pakistani isolates with the isolates from other parts of the world. The analysis involved 132 complete genome sequences. Each component is labelled with letters representing its geographic origin: Aus Australia, B. Burundi, C. China, Co. Congo, E. Egypt, I. India, M. Malawi, U. USA, S. Samoa, RW. Rwanda, Si. Sri Lanka, P. Pakistan, Ph. Philippines, T. Taiwan, To. Tonga, In. Indonesia, TH. Thailand. Pakistan isolates were shown in bold. The analysis showed two geographic regions in BBTV i.e Pacific Indian Ocean and Southeast Asia. Within the PIO Pakistan isolates showed homology with Indian, Samoa, Tonga Egypt, Australia and USA isolates. While SEA group includes isolates from China, Taiwan, Philippine, Thailand, Indonesia and a reassorted isolate of India.

**Fig 2:**
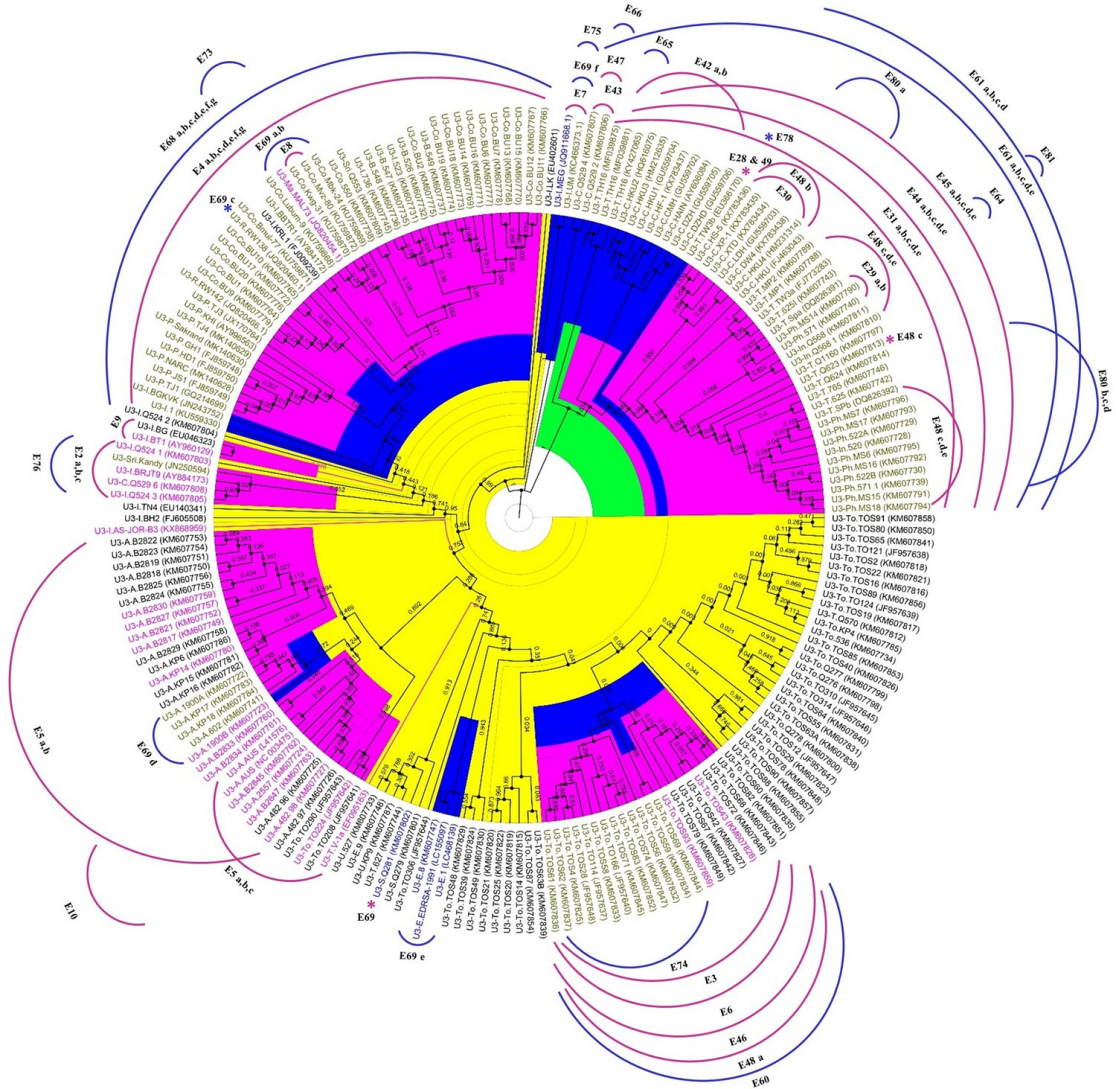
Phylogenetic analysis of BBTV DNA-U3 component illustrating intra and intergenomic recombination events at different nodes. Phylogenetic analysis of DNA-U3 based on nucleotide sequences of BBTV isolates from Pakistan along with previously reported PIO and SEA isolates of BBTV. The evolutionary history was inferred using the Maximum likelihood (ML) method. The percentage of replicate trees in which the associated taxa clustered together in the bootstrap test (1000 replicates). Each sequence is labelled with the GenBank accession number followed by origin and isolate name. The phylogenetic trees were further annotated in FigTree software v1.4.3 (Rambaut, 2010). Light green colour in the circle represents SEA population, while yellow colour represents PIO population. Similar analysis of DNA-R, -S, -M, -C and -N were performed and provided as supplementary figures (S1–S5 Figs) Intergenomic recombination events were marked with pink arc, while Intragenomic recombination events marked with blue arc. Isolates involved in both events were coloured brown. E represents the number of events and a,b,c,d etc. shows different populations in one event detected in RDP. Asterisk sign (*) shows only one isolate was involved in recombination at the given node. Arrows on both sides indicates that all the isolates were involved in respective recombination event. Blue arrow on both sides indicates that all the isolates of PIO group were involved in respective recombination event. The recombinant events described in S2 and S3 Tables are marked. Due to recombination different isolates showed intra and intergenomic events at different nodes.

The phylogenetic relationship of BBTV isolates from different banana growing regions was analyzed to determine the relationships of isolates from Pakistan with those from worldwide. For this purpose, a concatenated genome-based ML phylogenetic tree was generated. The tree includes a total of 132 BBTV genomes out of which 107 were from PIO and 25 were from SEA group. The phylogenetic analysis showed close homology of Pakistan isolates with each other making a single clade with close relationships with isolates from Congo, Sri Lanka, Rwanda, Malawi, Burundi and India with a bootstrap support of 78% (Fig 1) in PIO. In addition, the multiple sequence alignments of a total 345 full-length DNA-R sequences (266 from PIO and 79 from the SEA), 208 full-length sequences of DNA-U3 (166 from PIO and 42 from SEA), 274 full-length sequences of DNA-S (214 from PIO and 60 from SEA), for DNA-M, 206 full-length sequences (164 from PIO and 42 from SEA), 194 full-length sequences of DNA-C (156 from PIO and 38 from SEA) and for DNA-N a total of 198 full-length sequences (154 from PIO and 44 from SEA) were conducted by MAFFT for each component separately and phylogenetic relationships were inferred using Maximum Likelihood (ML) method with 1000 bootstrap replications using MEGA X and Newick format files were also generated and annotated in FigTree version 1.4.3 [44] (Fig 2, S1–S5 Figs. This analysis revealed a clear-cut partitioning of BBTV isolates into two major clusters (PIO and SEA group) [57]. Interestingly, though Pakistani BBTV population phylogenetically belonged to PIO based on all the genetic components however, their genetic diversity pattern (Table 3) is quite distinct compared to the PIO group (Table 4). Notably, the most conserved component in BBTV Pakistan is DNA-R, while in PIO it is DNA-C, while most conserved functional region in BBTV Pakistan is CR-SL (identical in DNA-R, -S, -M and -C) while in PIO it is CR-M of DNA-C.

### Population genetics and selection pressure

To understand the evolutionary selection pressure on various BBTV genomic components, the nonsynonymous to synonymous substitutions rate per site were calculated (Table 5) for coding regions of each component. For all the components (DNA-R, -S, -M, -C, -N and -U3 in many geographic regions), the d_N_ values (ranging from 0.001–0.062) for the coding region were smaller than d_S (_ranging from 0.002–0.352) and the d_N_/d_S_ ratios (ranging from 0.031–0.612) were <1, indicating negative/purifying selection, except for DNA-U3 where d_N_/d_S_ ratio was different between different geographic populations (Table 5). In Pakistani, Taiwanese, Egyptian and Tongan population DNA-U3 showed negative/purifying selection (d_N_/d_S_ ratio <1), while for Australian, Congo and USA populations showed positive/diversifying selection (d_N_/d_S_ ratio >1) while population from India and Samoa showed neutral (d_N_/d_S_ ratio = 1) selection pressure. The data of selection pressure indicated that the coding regions of all the components (except DNA-U3) of BBTV genome in various geographic populations are under purifying selection pressure. The purifying selection pressure is known to preserve the biological function and help in removal of deleterious mutations [59, 60] indicating that proteins encoded by BBTV genomic components are essential and any deleterious mutations occurring in their genes are eliminated from these geographic populations. However, DNA-U3 is interesting exception to this general observation noted for BBTV genome. In Congo it is under high positive selection with dN/dS value of 3.828. While it is under moderate positive selection in USA and Australia (dN/dS value of 1.773 and 1.496 respectively) and under neutral selection pressure in India and Samoa (with dN/dS value of 1.070 and 1.038 respectively) (Table 5).

**Table 5:** Component wise population genetic parameters of Banana bunchy top virus.

Component	Population	d_N_	d_S_	d_N_/d_S_	π (CDS)	π (Full length)
**DNA-R**	Australia (n = 37)	0.00207 (0.00089) ^a^	0.00884 (0.00398) ^a^	0.234163	0.35	0.46
	Burundi (n = 3)	0.00398 (0.00194)	0.00724 (0.00497)	0.549724	0.46	0.72
	China (n = 9)	0.00690 (0.00203)	0.11547 (0.01807)	0.059756	2.80	3.00
	Congo (n = 69)	0.00142 (0.00052)	0.01960 (0.00479)	0.072449	0.52	0.87
	Egypt (n = 5)	0.01424 (0.00312)	0.15724 (0.02306)	0.090562	3.93	4.85
	India (n = 59)	0.01092 (0.00191)	0.09927 (0.01252)	0.110003	2.75	2.95
	Indonesia (n = 17)	0.00195 (0.00084)	0.01734 (0.00472)	0.112457	0.51	0.62
	Japan (n = 7)	0.00171 (0.00099)	0.01814 (0.00583)	0.094267	0.52	0.43
	Pakistan (n = 24)	0.00062 (0.00026)	0.00393 (0.00227)	0.157761	0.13	0.26
	Philippine (n = 16)	0.00145 (0.00078)	0.01453 (0.00456)	0.099794	0.42	0.60
	Rwanda (n = 2)	0.00149 (0.00145)	0.00540 (0.00528)	0.275926	0.23	0.54
	Samoa (n = 2)	0.00149 (0.00144)	0.04433 (0.01509)	0.033612	1.04	1.17
	Sri Lanka (n = 3)	0.0079 (0.00301)8	0.04654 (0.01313)	0.171465	1.58	1.53
	Myanmar (n = 3)	0.00099 (0.00099)	0.02191 (0.00841)	0.045185	0.54	0.84
	Taiwan (n = 13)	0.00869 (0.00185)	0.08537 (0.01217)	0.101792	2.25	3.02
	Tonga (n = 55)	0.00493 (0.00110)	0.02075 (0.00421)	0.23759	0.81	1.05
	Vietnam (n = 9)	0.01312 (0.00271)	0.16801 (0.02192)	0.078091	4.18	4.33
	Total (n = 333)	0.01252 (0.00229)	0.16648 (0.01825)	0.075204	3.98	4.45
**DNA-U3**	Australia (n = 29)	0.02309 (0.01033)	0.01543 (0.00691)	1.496436	2.07	1.45
	Congo (n = 21)	0.02148 (0.00797)	0.00561 (0.00398)	3.828877	1.69	2.10
	Egypt (n = 4)	0.02045 (0.00863)	0.02876 (0.01665)	0.711057	2.20	2.97
	India (n = 9)	0.05893 (0.01346)	0.05504 (0.01372)	1.07067	5.38	8.60
	Pakistan (n = 7)	0.00348 (0.00242)	0.00447 (0.00454)	0.778523	0.36	0.56
	Samoa (n = 2)	0.01746 (0.00980)	0.01681 (0.01691)	1.038667	1.70	1.79
	Taiwan (n = 2)	0.06266 (0.02339)	0.09895 (0.04278)	0.633249	6.83	3.97
	Tonga (n = 37)	0.02590 (0.00961)	0.03434 (0.01250)	0.754222	2.74	3.78
	USA (n = 2)	0.01460 (0.01063)	0.00823 (0.00817)	1.773998	1.28	0.66
	Total (n = 121)	0.03358 (0.01133)	0.04488 (0.01309)	0.748217	3.86	5.41
**DNA-S**	Australia (n = 34)	0.00766 (0.00193)	0.03630 (0.00955)	0.211019	1.37	1.50
	Burundi (n = 5)	0.00697 (0.00283)	0.02353 (0.00990)	0.296218	1.06	1.17
	China (n = 8)	0.00892 (0.00328)	0.10067 (0.02055)	0.088606	2.76	3.64
	Congo (n = 25)	0.00498 (0.00165)	0.03648 (0.00979)	0.136513	1.18	1.22
	Gabon (n = 2)	0.00998 (0.00492)	0.03371 (0.01644)	0.296055	1.51	1.58
	India (n = 45)	0.00648 (0.00158)	0.09444 (0.01666)	0.068615	2.47	3.56
	Indonesia (n = 7)	0.00331 (0.00168)	0.03558 (0.01175)	0.09303	1.04	1.0
	Japan (n = 3)	0.00166 (0.00160)	0.01663 (0.00929)	0.09982	0.50	0.63
	Malawi (n = 2)	0.00497 (0.00341)	0.04244 (0.01873)	0.117107	1.32	1.02
	Pakistan (n = 22)	0.00259 (0.00098)	0.01434 (0.00556)	0.180614	0.51	0.48
	Philippine (n = 17)	0.00450 (0.00186)	0.02594 (0.00853)	0.173477	0.92	0.62
	Samoa (n = 2)	0.00248 (0.00246)	0.07822 (0.02541)	0.031705	1.89	1.48
	Sri Lanka (n = 3)	0.00497 (0.00280)	0.03958 (0.01471)	0.125568	1.26	1.42
	Taiwan (n = 15)	0.01076 (0.00299)	0.09035 (0.01612)	0.119092	2.60	4.19
	Tonga (n = 58)	0.00460 (0.00140)	0.02736 (0.00876)	0.168129	0.96	1.18
	USA (n = 2)	0.00248 (0.00242)	0.01663 (0.01162)	0.149128	0.56	0.27
	Vietnam (n = 2)	0	0.00824 (0.00809)	0	0.18	1.20
	Total (n = 252)	0.01203 (0.00322)	0.15249 1 (0.02345)	0.080661	3.96	5.46
**DNA-M**	Australia (n = 38)	0.01772 (0.00361)	0.06685 (0.01724)	0.265071	2.79	2.17
	Burundi (n = 4)	0.00375 (0.00260)	0.02653 (0.01359)	0.141349	0.89	0.89
	China (n = 9)	0.03767 (0.00693)	0.08435 (0.02027)	0.446592	4.60	5.33
	Congo (n = 22)	0.01404 (0.00350)	0.03646 (0.01202)	0.38508	1.87	2.01
	Egypt (n = 2)	0.01505 (0.00739)	0.06313 (0.02917)	0.238397	2.82	3.15
	India (n = 13)	0.03377 (0.00547)	0.07776 (0.01687)	0.434285	4.12	3.63
	Indonesia (n = 3)	0.01527 (0.00611)	0.05001 (0.02125)	0.305339	2.27	1.21
	Pakistan (n = 9)	0.00166 (0.00117)	0.00271 (0.00264)	0.612546	0.18	0.33
	Philippine (n = 12)	0.00775 (0.00318)	0.04778 (0.01594)	0.162202	1.66	1.06
	Samoa (n = 2)	0.00373 (0.00369)	0.02464 (0.01779)	0.15138	0.84	0.66
	Sri Lanka (n = 3)	0.01001 (0.00489)	0.03304 (0.01668)	0.302966	1.50	1.69
	Taiwan (n = 9)	0.01701 (0.00481)	0.03682 (001427)	0.461977	2.10	2.37
	Thailand (n = 2)	0.00378 (0.00372)	0.01174 (0.01144)	0.321976	0.56	1.24
	Tonga (n = 64)	0.02751 (0.00566)	0.07546 (0.01861)	0.364564	3.67	3.26
	USA (n = 2)	0.01127 (0.00643)	0.02454 (0.01773)	0.45925	1.41	0.66
	Total (n = 194)	0.04629 (0.00790)	0.13683 (0.02857)	0.33830	8.17	8.21
**DNA-C**	Australia (n = 39)	0.00483 (0.00183)	0.02081 (0.00824)	0.2321	0.81	0.92
	Burundi (n = 4)	0.01294 (0.00422)	0.01752 (0.01004)	0.738584	1.37	1.09
	China (n = 11)	0.03415 (0.00627)	0.16481 (0.02479)	0.207208	5.58	5.59
	Congo (n = 22)	0.00524 (0.00185)	0.02390 (0.00817)	0.219247	0.91	1.34
	Egypt (n = 2)	0.07069 (0.01319)	0.35203 (0.06966)	0.200807	11.31	13.25
	India (n = 12)	0.04324 (0.00539)	0.10264 (0.01637)	0.421278	5.14	4.62
	Indonesia (n = 3)	0.00708 (0.00349)	0.02604 (0.01270)	0.271889	1.09	0.92
	Pakistan (n = 5)	0.00106 (0.00103)	0.01548 (0.00751)	0.068475	0.41	0.47
	Philippine (n = 12)	0.00463 (0.00218)	0.02141 (0.00908)	0.216254	0.81	0.81
	Sri Lanka (n = 3)	0.00711 (0.00346)	0.07307 (0.02109)	0.097304	2.05	2.43
	Taiwan (n = 9)	0.02073 (0.00383)	0.08691 (0.01680)	0.238523	3.14	4.04
	USA (n = 2)	0.00265 (0.00260)	0.00958 (0.00934)	0.276618	0.41	0.19
	Tonga (n = 60)	0.00655 (0.00194)	0.03476 (0.00955)	0.188435	1.23	1.28
	Total (n = 184)	0.02849 (0.00409)	0.13653 (0.02201)	0.208672	4.60	4.82
**DNA-N**	Australia (n = 35)	0.00297 (0.00146)	0.01753 (0.00751)	0.169424	0.60	0.52
	Burundi (n = 4)	0.00279 (0.00194)	0.00988 (0.00692)	0.282389	0.43	0.44
	China (n = 15)	0.01732 (0.00508)	0.15771 (0.02774)	0.109822	4.34	5.54
	Congo (n = 22)	0.00441 (0.00149)	0.02111 (0.00831)	0.208906	0.79	1.25
	India (n = 10)	0.01684 (0.00335)	0.08905 (0.01723)	0.189107	2.90	4.36
	Indonesia (n = 3)	0.00187 (0.00186)	0.03949 (0.01559)	0.047354	1.00	0.73
	Pakistan (n = 5)	0 (0)	0.00396 (0.00395)	0	0.86	0.22
	Philippine (n = 11)	0.00143 (0.00142)	0.02524 (0.00927)	0.056656	0.66	0.72
	Samoa (n = 3)	0.03056 (0.00828)	0.28592 (0.05765)	0.106883	7.16	15.04
	Sri Lanka (n = 2)	0.01119 (0.00549)	0.04028 (0.01965)	0.277805	1.72	3.75
	Taiwan (n = 11)	0.01037 (0.00267)	0.10100 (0.01865)	0.102673	2.62	3.64
	Tonga (n = 62)	0.00406 (0.00169)	0.02781 (0.00971)	0.145991	0.90	1.53
	USA (n = 2)	0	0.03010 (0.01734)	0	0.86	0.45
	Total (n = 185)	0.01752 (0.00374)	0.18114 (0.02986)	0.096721	4.63	7.09

**Note:** n, number of isolates, dS, number of synonymous substitutions per site; dN, number of nonsynonymous substitutions per site, ^a^ Numbers in parentheses are the standard errors, isolates whose CDS were not given by the authors in GenBank were not included in the analysis; π, denotes pair-wise average nucleotide diversity per 100 sites.

The DNA-U3 encodes U3 protein for which any function is not yet been determined, however, it is an integral [5] and essential component for infection as demonstrated by infectivity assay for nanoviruses [61, 62]. Despite being essential component, the higher dN/dS values noted for DNA-U3 in some countries where rest of the components are under purifying selection, indicates that some significant factors are at play differently for DNA-U3. Interestingly, three out of four DNA-U3 components analyzed in this study were previously reported to have undergone recombination in Burundi [31], similarly, all of the DNA-U3 components of India and most of the components from Samoa, USA and Australia have also been reported for recombination [31] thus pointing toward the possible role of recombination in observed positive selection seen in DNA-U3 in some countries.

Similarly, the Indian subcontinent is noted as a major hub [31] of long-distance banana and BBTV movements. Stainton and colleagues (2015) indicated it as both the major donor location (for BBTV dispersal events to other parts of the world) and the major recipient location (of virus introductions in it). In general, BBTV isolates from the PIO group are found throughout the natural geographical range of *Musa balbisiana*, whereas isolates from the SEA group are found throughout the ranges of *M*. *balbisiana* and *M*. *acuminata* [63]. However, the banana germplasm of some Indian regions primarily comprised of hybrids between *M*. *balbisiana* from the Indian subcontinent and *M*. *acuminata* from Southeast Asia [63, 64]. This might explain the neutral pressure seen in DNA-U3 in India isolates. Different selection pressure was also observed among different banana genome types [30]. Later, Chiaki and colleagues (2015) also noted that selection pressure was higher in viruses infecting banana varieties with the AAB or ABB genotypes than those infecting with AA or AAA genotypes. The data of selection pressure in present study, suggests that BBTV genome is under negative selection pressure, however, the coding regions of DNA-U3 in some geographic regions around the word are under neutral and positive selection which might be due to mixing of isolates from PIO and SEA groups, and/or due to virus propagation in hosts of different genetic backgrounds and/or due to recombination.

Haplotype diversity values were high for BBTV components ranging from 0.4–1.0 (Table 6). Tajima’s *D* values were calculated to determine the impact of demographic expansion and contraction in different BBTV DNA component populations. Negative values with statistical significance (P<0.02 or P<0.01) were obtained for DNA-R and DNA-C (Tonga population) and in the Australian population of DNA-S. These results were further confirmed by Fu and Li’s D and F statistical test values (Table 6). Which suggests that these populations may be under expansion phase. On the contrary, no statistically significant positive or negative values were found in the remaining populations of BBTV components suggesting that these populations may be undergoing a neutral or contraction period.

**Table 6:** Neutrality test, haplotype and nucleotide diversity of each BBTV population.

Component	Geographical group	Fu & Li’s D*	Fu & Li’s F*	Tajima’s D	Nucleotide diversity (π)	Number of Haplotype (H)	Haplotype diversity (Hd)
	Australia (n = 37)	-2.04035	-2.09769	-1.246	0.350 (3.015) ^a^	19	0.908
	China (n = 9)	-0.00578**	0.00812**	0.29796*	2.804 (24.139)	8	0.972
	Congo (n = 69)	-3.93364	-3.84941	-2.03161	0.529 (4.557)	30	0.928
	Egypt (n = 5)	-0.91326	-1.00473	-0.71713	3.937 (33.900)	4	0.900
	India (n = 59)	-0.56954	-1.16610	-1.22798	2.755 (23.720)	48	0.991
**DNA-R**	Indonesia (n = 17)	-1.25467	-1.56227	-1.37207	0.519 (4.470)	13	0.963
	Japan (n = 7)	-1.27255	-1.37953	-1.19248	0.520 (4.476)	7	1.000
	Pakistan (n = 24)	-3.12612	-3.22066	-1.91443	0.133 (1.141)	9	0.616
	Philippine (n = 16)	-1.55326	-1.65731	-1.14040	0.424 (3.650)	15	0.992
	Taiwan (n = 13)	-2.28488	-2.48829	-1.57368	2.251 (19.384)	11	0.962
	Tonga (n = 55)	-4.99012**	-4.81334**	-2.35564**	0.819 (7.047)	44	0.989
	Vietnam (n = 9)	-0.04727	-0.08322	0.51654	4.184 (36.027)	9	1.000
	Australia (n = 29)	-0.08497	0.00827	0.20706	2.143 (5.013)	28	0.995
	Burundi (n = 4)	0.95621	0.90358	0.95621	1.282 (3.000)	4	1.000
	China (n = 7)	-1.42725	-1.52246	-1.35841	0.824 (0.857)	4	0.714
	Congo (n = 21)	-1.55082	-1.59338	-0.77744	1.691 (3.957)	20	0.995
**DNA-U3**	India (n = 13)	-2.10432	-2.34211	-1.52051	3.901 (9.128)	11	0.962
	Egypt (n = 4)	-0.52807	-0.52801	-0.52807	2.208 (5.166)	3	0.833
	Pakistan (n = 7)	-1.42725	-1.52246	-1.35841	0.366 (0.8571)	3	0.524
	Philippine (n = 11)	-2.38446**	-2.55684**	-1.83180	7.564 (17.472)	11	1.000
	Tonga (n = 38)	-2.99753*	-2.89995*	-1.16514	2.731 (6.389)	37	0.999
	Australia (n = 34)	-4.33599**	-4.23105**	-1.978714*	1.376 (7.2656)	29	0.991
	Burundi (n = 5)	-0.20090	-0.21293	-0.20090	1.061 (5.600)	5	1.000
	China (n = 8)	0.24399	0.13415	0.27767	2.767 (14.607)	8	1.000
	Congo (n = 25)	-1.66789	-1.79259	-1.22107	1.188 (6.273)	20	0.983
	India (n = 45)	-3.21478*	-3.14296*	-1.33542	2.476 (13.075)	38	0.989
**DNA-S**	Indonesia (n = 7)	0.30430	0.31478	0.22467	1.046 (5.523	5	0.857
	Pakistan (n = 22)	-2.37337	-2.51062	-1.51140	0.518 (2.735)	9	0.658
	Philippine (n = 17)	-1.17566	-1.19675	-0.68156	0.927 (4.897)	15	0.978
	Taiwan (n = 15)	0.98697	0.62737	-0.44960	2.608 (13.771)	15	1.000
	Tonga (n = 58)	-4.84147**	-4.47257**	-1.88300	0.966 (5.102)	52	0.996
	Australia (n = 38)	-0.83943	-0.99304	-0.77740	2.794 (9.890)	35	0.994
	Burundi (n = 4)	-0.31446	-0.30226	-0.31446	0.895 (3.166)	4	1.000
	China (n = 9)	-0.79528	-0.89198	-0.79111	4.606 (16.166)	7	0.944
	Congo (n = 22)	-2.82192*	-2.94040*	-1.66120	1.873 (6.632)	21	0.996
**DNA-M**	India (n = 13)	-1.81015	-2.00681	-1.52243	4.121 (14.589)	11	0.974
	Pakistan (n = 9)	-1.68268	-1.82046	-1.51297	0.188 (0.666)	4	0.583
	Philippine (n = 12)	-0.59573	-0.65666	-0.09300	1.662 (5.833)	12	1.000
	Taiwan (n = 9)	0.43134	0.37198	0.02015	2.105 (7.388)	8	0.972
	Tonga (n = 64)	-2.41535	-2.28545	-0.91800	3.675 (13.010)	64	1.000
	Australia (n = 39)	-2.52616*	-2.49715	-1.29407	0.817 (3.973)	33	0.992
	Burundi (n = 4)	0.18677	0.18886	0.18677	1.372 (6.666)	4	1.000
	China (n = 11)	-0.02852	-0.17311	-0.14515	5.585 (27.145)	9	0.945
**DNA-C**	Congo (n = 22)	-2.31978	-2.39685	-1.23370	0.912 (4.432)	20	0.987
	India (n = 12)	-1.78246	-2.00370	-1.49321	5.144 (25.000)	10	0.970
	Pakistan (n = 5)	-1.12397	-1.15583	-1.12397	0.412 (2.000)	4	0.900
	Philippine (n = 12)	-1.04405	-0.98064	-0.34465	0.814 (3.954)	12	1.000
	Taiwan (n = 9)	-1.85984	-2.01186	-1.45335	3.144 (15.277)	9	1.000
	Tonga (n = 60)	-4.95616**	-4.65426**	-2.03254*	1.239 (6.023)	59	0.999
	Australia (n = 35)	-3.08712*	-3.03503*	-1.51902	0.607 (2.823)	22	0.956
	Burundi (n = 4)	-0.78012	-0.72052	-0.78012	0.430 (2.000)	3	0.833
	China (n = 15)	0.92233	0.73699	0.74237	4.340 (20.180)	12	0.971
	Congo (n = 22)	-2.46716	-2.62629*	-1.65832	0.794 (3.692)	20	0.991
**DNA-N**	India (n = 10)	-2.19752**	-2.39401*	-1.83388*	2.906 (13.511)	9	0.978
	Pakistan (n = 5)	-0.81650	-0.77152	-0.81650	0.086 (0.400)	2	0.400
	Philippine (n = 11)	-0.86187	-0.84325	-0.40305	0.665 (3.090)	11	1.000
	Taiwan (n = 11)	-2.02194	-2.20438	-1.65409	2.628 (12.218)	11	1.000
	Tonga (n = 62)	-4.81607**	-4.50832**	-1.79112	0.908 (4.222)	47	0.987

Note:

^a^ Numbers in parentheses are the average number of nucleotide differences, all isolates whose CDS were not given by the authors in GenBank were not included in the analysis, also populations having less than four isolates were excluded due to the software requirement, π, denotes pair-wise average nucleotide diversity per site.

* P<0.02

** P<0.01.

### Recombination analysis

A detailed analysis on full-length sequences of BBTV genomic components using various methods implemented in the Recombination Detection Program (RDP), reveals many recombination events of intergenomic (homologous recombination between the same components) (S2 Table) and intragenomic (heterologous recombination between the two different components) (S3 Table) in BBTV genome. There are about fifty-four (66%) intergenomic recombination events out of total eighty-two events, while there are twenty-eight (34%) intragenomic recombinant events. Component wise, DNA-U3 is involved in the majority (thirty-five events, about 43%) of detected recombination events, followed by DNA-M (thirteen events, about 16%) while DNA-R and -N are involved in only nine (11%) events each. The data of recombination occurrence for each component revealed that DNA-S which encodes coat protein of BBTV has the least recombination occurrence of 1.8% (5 recombination events from 274 components) followed by DNA-R with 2.6% (9 recombination events detected in 345 components) (Table 7). It is worthy to note that DNA-U3 showed the highest 16.8% (35 recombination events occurred in 208 component) occurrence of recombination in BBTV genome which is about 9 times higher than the least recombined component of DNA-S. Interestingly, the DNA-U3 which was found to have the greatest genetic diversity (Table 4) shows the highest involvement in the recombination events occurring in BBTV genome, while DNA-R that is the most conserved component, is also among the least recombined components in BBTV genome.

**Table 7:** Occurrence of recombination in Banana bunchy top virus genome.

Components	Among PIO group			Among SEA group			Between PIO & SEA group			Entire Population		
	Number of isolates	Number of Recombination Events	Percentage	Number of isolates	Number of Recombination Events	Percentage	Number of isolates	Number of Recombination Events	Percentage	Number of isolates	Number of Recombination Events	Percentage
**Intergenomic Recombination**												
**DNA-R**	266	1	0.37	79	1	1.26	345	4	1.16	345	6	1.73
**DNA-U3**	166	9	5.42	42	4	9.52	208	8	3.84	208	21	10.09
**DNA-S**	214	2	0.93	60	1	1.66	274	1	0.36	274	4	1.45
**DNA-M**	164	4	2.43	42	2	4.76	206	2	0.97	206	8	3.88
**DNA-C**	156	4	2.56	38	2	5.26	194	2	1.03	194	8	4.12
**DNA-N**	154	6	3.89	44	1	2.27	198	0	0	198	7	3.53
**Total**	1425	26	1.82	305	11	3.60	1425	17	1.19	1425	54	3.78
**Intragenomic Recombination**												
**DNA-R**	266	1	0.37	79	0	0	345	2	0.57	345	3	0.86
**DNA-U3**	166	6	3.01	42	3	7.14	208	5	2.40	208	14	6.73
**DNA-S**	214	0	0	60	0	0	274	1	0.36	274	1	0.36
**DNA-M**	164	2	1.21	42	1	2.38	206	2	0.97	206	5	2.42
**DNA-C**	156	0	0	38	0	0	194	3	1.54	194	3	1.54
**DNA-N**	154	1	0.37	44	1	2.27	198	0	0	198	2	1.51
**Total**	1425	10	0.70	305	5	1.63	1425	13	0.91	1425	28	2.175

**Note**: The population *n* for each components and subgroups are as follows: DNA-R [*n*_(total)_ = 345 {*n*_(PIO)_ = 266, *n*_(SEA)_ = 79}], DNA-U3 [*n*_(total)_ = 208 {*n*_(PIO)_ = 166, *n*_(SEA)_ = 42}], DNA-S [*n*_(total)_ = 274 {*n*_(PIO)_ = 214, *n*_(SEA)_ = 60}], DNA-M [*n*_(total)_ = 206, {*n*_(PIO)_ = 164, *n*_(SEA)_ = 42}], D*N*A-C [*n*_(total)_ = 194 {*n*_(PIO)_ = 156, *n*_(SEA)_ = 38}] and DNA-N [*n*_(total)_ = 198 {*n*_(PIO)_ = 154, *n*_(SEA)_ = 44}] where ‘total’ means all the sequence of isolates of particular genomic component.

To understand the contribution of recombination in BBTV genetic diversity, the recombined regions identified by RDP for the respective events were selected and the diversity analysis of these geographic populations with and without recombinants was performed. The analysis (Table 8) revealed that recombination is responsible to increase the genetic diversity of many of the geographic populations which harbor recombinant isolates. In some instances (recombination event (1), Table 8), it is responsible to increase the diversity of about 4 times due to the recombinants in those populations. In some cases (recombination events (3), (15), (16), (17), (18), (33), (38), (39), (40), (48), (50), (53), (67), (72), Table 8) the diversity of recombinant population is less than their non-recombinant population, however, the average of the entire dataset (for which recombinant and non-recombinant population of certain geographic region was available) showed a net contribution of about 1.4 times suggesting a significant contribution by recombination in BBTV genetic diversity.

**Table 8:** Contribution of genetic diversity by recombinant isolates in their population.

Recombination Event		Diversity without recombinants			Diversity with recombinants			Fold Increase	
Subpopulations		Identity Range	π	θw	Identity Range	π	θw	π	θw
**INTERGENOMIC RECOMBINATION**									
**Among PIO subgroup**									
DNA-R (1)	India	98.7–99.4	0.83±0.22	1.00±0.61	98.7–99.4	3.83±1.95	5.03±2.48	4.6	5.03
DNA-U3 (3)	Tonga	87–99.6	4.49±0.32	4.80±1.61	86.9–99.6	3.18±0.53	3.76±1.41	0.70	0.78
DNA-U3 (5)	Australia	98.5–100	0.98±0.21	0.83±0.49	92.6–100	2.58±0.55	2.59±1.01	2.63	2.64
DNA-U3 (6)	Tonga	64.0–99.7	14.47±3.27	12.55±5.08	59.6–99.7	17.08±1.36	10.85±3.85	1.18	0.86
DNA-U3 (10)	Tonga	100	0.36±0.18	0.36±0.36	99.5–99.7	0.72±0.25	0.72±0.55	2	2
DNA-M (15)	Congo	97.9–100	0.77±0.21	1.08±0.58	90.8–100	1.73±0.55	3.37±1.31	2.24	3.12
	Tonga	95.7–99.3	1.54±0.43	1.59±0.97	95.7–100	1.22±0.27	1.50±0.76	0.79	0.94
DNA-M (16)	Congo	97.8–98.0	1.96±0.57	1.88±1.20	97.8–98.9	1.53±0.35	1.54±0.91	0.78	0.81
DNA-C (17)	India	94.2–99.8	2.16±0.81	2.72±1.23	94.0–99.4	2.15±0.74	2.87±1.26	0.99	1.05
DNA-C (18)	India	92.8–99.8	2.91±0.97	3.22±1.43	93.1–99.8	2.74±0.92	3.19±1.38	0.94	0.99
DNA-C (20)	India	83.2–100	4.89±3.08	6.93±3.35	83.2–100	7.24±2.79	6.61±3.12	1.48	0.95
DNA-N (22)	Tonga	88.2–100	6.16±1.05	4.80±3.03	77.1–100	12.81±4.92	14.74±7.32	2.07	3.07
DNA-N (26)	Tonga	58.3–100	2.01±0.26	3.44±1.04	57.7–100	2.86±0.27	3.54±1.04	1.42	1.02
**Among SEA subgroup**									
DNA-U3 (30)	China	94.9–100	3.52±2.09	4.23±2.98	62.7–100	7.64± 2.99	7.72±4.56	2.17	1.82
DNA-M (33)	Philippine	99.1–100	0.26±0.19	0.41±0.41	98.2–100	0.23±0.17	0.39±0.39	0.88	0.95
**Between PIO and SEA subgroups**									
DNA-R (38)	Congo	93.0–100	2.81±0.51	2.23±1.06	93.0–100	2.74±0.48	2.13±0.99	0.97	0.95
	Tonga	91.8–100	2.25±0.73	2.20±0.98	87.8–100	2.92±0.77	3.25±1.31	1.29	4.45
	Indonesia	96.3–100	1.29±0.23	0.94±0.56	96.3–100	1.34±0.18	0.91±0.54	1.03	0.96
	India	89.5–100	6.12±0.36	6.12±4.56	78.3–100	7.27±1.49	7.18±3.80	1.18	1.17
	Taiwan	97.0	2.89±1.44	2.89±2.22	94.0–97.0	5.31±1.83	5.31±3.42	1.83	1.83
DNA-R (39)	China	99.3–100	0.91±0.30	0.91±0.75	99.3–100	0.79±0.25	0.74 ±0.60	0.86	0.65
	Congo	97.9–99.3	2.74±0.81	3.42±1.37	97.9–99.3	2.95±0.67	3.46±1.34	1.07	1.01
	India	85.6–100	2.77±1.02	3.86±1.74	85.5–100	2.83± 0.85	3.98± 1.69	1.02	1.03
	Tonga	49.0–100	2.24± 0.46	3.65± 1.34	49.3–100	2.50± 0.43	3.89± 1.28	1.11	1.06
DNA-R (40)	India	97.8–100	0.51+0.14	0.85+0.54	97.8–100	0.50±0.13	0.82±0.51	0.98	0.96
	Congo	92.2–100	1.33+0.46	1.33+1.00	92.2–100	1.33+0.36	1.45+0.99	1	1.09
	Tonga	97.8–100	0.28+0.10	1.25+0.64	94.4–100	0.40+0.17	1.70+0.77	1.43	1.36
	Australia	98.9–100	0.74+0.34	0.74+0.74	98.9–100	0.74+0.22	0.60+0.60	1	0.81
DNA-R (41)	India	99.0–100	0.57±0.16	0.60±0.37	99.0–100	0.60±0.13	0.65±0.38	1.05	1.08
DNA-U3 (44)	China	97.9	2.23±1.11	2.23±1.76	83.1–100	8.64±1.28	8.45±3.16	3.87	3.78
DNA-U3 (45)	China	89.9–100	5.17±0.40	4.01±1.66	84.5–100	6.82± 0.96	5.87± 2.27	1.32	1.46
DNA-U3 (46)	Tonga	95.6–100	3.32±0.64	2.77± 1.54	80.7–100	7.80± 3.28	9.36±4.35	2.34	3.37
DNA-U3 (48)	Philippine	93.9–97.0	4.54±1.10	4.95±3.59	87.9–97.0	7.21±1.26	7.42±4.27	1.59	1.49
	Taiwan	93.9	6.06±3.03	6.06±5.24	90.9–100	4.44±0.94	3.98± 2.75	0.73	0.66
	Tonga	87.9–100	7.011±0.61	6.16±3.04	72.7–100	8.57±1.53	10.95±4.62	1.22	1.77
DNA-S (50)	Australia	92.4	15.04±7.52	15.07±10.75	97.9–100	0.76±0.08	1.50±0.51	0.05	0.09
	Pakistan	99.4	0.67±0.33	0.67±0.53	98.3–100	0.42±0.11	1.05±0.40	0.62	1.56
	Taiwan	98.9–99.4	0.88±0.32	0.88±0.63	98.9–100	0.81±0.12	0.96±0.46	0.92	1.09
DNA-C (53)	China	89.6–100	6.22±2.93	6.22±4.16	87.0–100	4.80±1.43	5.00±2.40	0.80	0.89
**INTRAGENOMIC RECOMBINANT**									
**Among PIO and SEA subgroup**									
DNA-C (67)	Australia	99.1–100	1.70±0.52	1.39±1.12	98.6–100	1.83±0.37	1.57±1.07	1.07	1.12
	Congo	83.3–92.2	15.78±5.29	15.78±9.89	85.7–100	8.86±2.37	10.94±5.58	0.56	0.69
	Tonga	97.0–100	1.76±0.29	2.40±1.24	97.0–100	1.32±0.17	1.82±0.94	0.75	0.75
	Taiwan	88.4–100	4.90±2.50	6.44±3.51	67.1–100	11.11±4.18	11.45±5.71	2.26	1.77
**Among PIO subgroup**									
DNA-U3 (69)	Congo	98.6–100	1.33±0.62	1.33±1.33	98.6–100	1.35±0.53	1.07±1.09	1.01	0.80
DNA-N (70)	Tonga	96.1–100	0.50±0.22	0.49±0.49	90.9–100	1.24±0.62	1.95±1.17	2.48	3.98
DNA-M (71)	Congo	94.2	7.69±3.84	7.69±5.95	88.4–96.2	12.82± 4.6	12.30 7.88	1.66	1.59
DNA-R (72)	Congo	94.7–96.0	4.27±1.21	4.05±2.65	94.0–100	1.98±0.32	2.45±1.03	0.46	0.60
	Tonga	61.7–100	2.35±0.59	3.21±1.29	62.2–100	2.46±0.44	0.30±1.12	1.04	0.09
**Average**								**1.4**	**1.5**

**Note:** The analyses are performed only for those recombined regions in different genomic components which were identified to have undergone Intergenomic & Intragenomic recombination detected by Recombination Detection Program (RDP) version 4 Beta14 (Martin et al., 2015) No. in parenthesis () represents those recombination events for which recombinant and non-recombinant population of certain geographic region was available, the recombination events involving entire population at a certain geographic region was not included due to unavailability of non-recombinant population.

**Fig 3:**
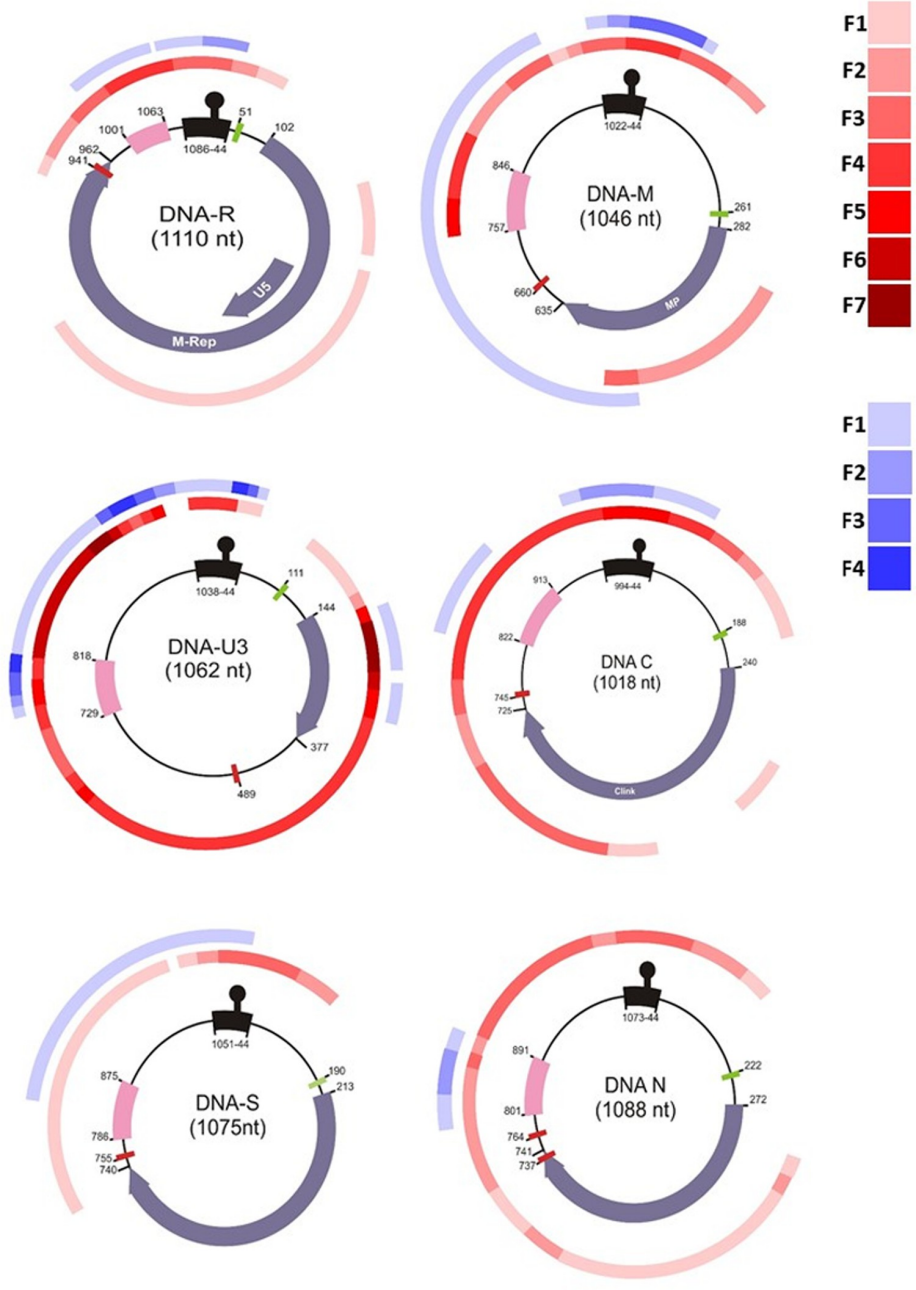
Recombination map of Banana bunchy top virus genome. The genomic components of BBTV are represented with inner circle having CR-SL, CR-M and ORF in black, pink and blue colours respectively. The nucleotide coordinates are corresponding to P.TJ1 isolates for reference. The red circles represent the intergenomic recombination, while the blue circle represent the intragenomic recombination. The intensity of colours was used to depict the frequency of recombination at a particular region using the key above. The recombinant events described in S2 and S3 Tables are marked. The figures were not drawn on scale.

The frequency of recombined regions in BBTV genome was analyzed by plotting them on various components (Fig 3) using TJ1 as a reference for nucleotides coordinates. The data show that DNA-U3 is highly involved both in inter and intragenomic recombination and DNA-R and DNA-S are least recombined components. In the BBTV genome, CR-SL, a functional regulatory region is identified as the recombinant hotspot for both intragenomic and intergenomic recombination.

## Discussion

Understanding the genetic structure of virus populations and their evolutionary mechanisms is an important aspect of managing viral diseases [65]. In Pakistan, information on the molecular analysis of BBTV was very limited with only a partial genome [37] and few full-length DNA components sequenced and deposited within the public database in GenBank [16, 25, 37]. However, inferences based on full-length BBTV genomes suggest that genetic exchange by recombination and reassortment might have played an important role in BBTV evolution around the world [31]. Therefore, fifty-seven genomic components including five complete genomes were sequenced in this study from Pakistan and their genetic diversity and recombination analyses was performed.

In the present study to understand existing variability in BBTV population, a detailed analysis of genetic diversity of its all component was performed first for the Pakistani population and later for the entire population of BBTV in the world. The genetic diversity data of Pakistani population (Table 3) suggested that BBTV full-length components diversity (ℼ) ranged from 0.26 to 0.69 pair-wise average nucleotide diversity per 100 sites, with Watterson estimator (θw) for population mutations rate of 0.36 to 0.99 per 100 sites in the country. The analysis showed that DNA-R a highly conserved component has the most diverse Common Region-Major (CR-M) while DNA-U3, a highly diverse component with the most conserved Common Region-Major (CR-M) and most diverse Common Region Stem-loop (CR-SL) (Table 3). An earlier study of recombination in CR-SL of DNA-U3 from Pakistan has also verified the diverse nature of this subgenomic regulatory region [16]. The analysis also indicated the heterogeneity of genetic diversity values associated with each component and their different parts, the overall diversity remained relatively small compared to the previously reported values for the Pacific Indian Ocean group [4, 26, 58]. Which has been split from the total population of BBTV probably a much larger time than the first introduction of BBTV in 1988 in Pakistan.

The genetic diversity of entire BBTV population around the world (Table 4) was also calculated and compared with the diversity analysis of Pakistani population. In contrary to the existing BBTV diversity, diversity analysis of Pakistani BBTV population showed that the CR-M of DNA-U3, is the most diverse CR-M in the entire BBTV population. The analysis also confirmed the heterogeneity of genetic diversity values associated with each component and their different parts, through the diversity values (ℼ) for full-length sequences ranged between 4.42 to 8.54 pair-wise average nucleotide diversity per 100 sites, with Watterson estimator (θw) for population mutations rate of 6.61 to 11.07 per 100 sites, greater of an order of 10 to 20 times than the Pakistani population, suggesting that other factors might be at play for this observed diversity. Multiple sequence alignment of full-length DNA-R component worldwide shares 92–100% and 89.8–100% sequence identity (Table 4) with members of PIO and SEA group respectively. Which shows on average about 3% and 7% increase in PIO and SEA group as compared to earlier studies [15, 26, 33, 58] while in the case of CR-M and CR-SL on average increase was approximately 38% and 18.8% respectively in SEA population. For PIO population increase was about 12.3% and 1.85% in CR-M and CR-SL respectively. Notably multiple sequence alignment of full-length components of Pakistan population showed DNA-R, the most conserved component with 99.1–100% sequence identity while CR-SL was the conserved functional region with 100% sequence identity in all components except DNA-U3 and DNA-N with sequence identities of 90.2–100% and 98.8–100% respectively (Table 3). While multiple sequence analysis of full-length components in PIO showed DNA-C as most conserved component (92.4–100%) and CR-M of DNA-C as conserved functional region (92.1–100%) (Table 4). These differences of genetic diversity in various components of Pakistani BBTV population compared to PIO group might be due to various factors such as differential evolutionary pressure on important conserved proteins encoded by respective components or due to reassorted genomic components introduced in the country belonging to different regions. However, the phylogenetic analysis of individual genomic components (Fig 2, S1–S5 Figs and full entire genomic ML phylogenetic analysis (Fig 1) indicate that all Pakistani isolates clustered together as a clade of BBTV indicating that they have a common origin. This observation argues strongly against any possible reassortment. Therefore, the observed difference in the genetic diversity values of Pakistan isolates compared to PIO group, is not due to different origin rather it might be due to different evolutionary pressure on each component and/or possible recombination. The phylogenetic analysis of full-length DNA-R component (S1 Fig) showed that Pakistan isolates have closed homology with Egyptian and Indian isolates. Which is similar to previous studies [25, 37, 66] based on DNA-R full-length where Pakistan isolates have close homology with Egypt, India and Australia isolates. Similar analysis for other full-length BBTV components (DNA-U3, -S, -M and -N) indicated close homology of Pakistan isolates with India (Fig 2, S3–S5 Figs). While in DNA- C (S4 Fig) the order of homology of Pakistan isolates with other PIO isolates was Egypt, Sri Lanka and India respectively. Notably the phylogenetic analysis of concatenated sequences of BBTV (Fig 1) showed clustering of Pakistan isolates within PIO which shows its homology with members of this region. However, within the PIO, Pakistan isolates have close homology with isolates from India, Congo, Sri Lanka, Rwanda, Malawi, and Burundi. Based on entire genome wise and individual component wise (DNA-U3, -S, -M and -N) ML analyses it is evident that Pakistani isolates have very close relationships with the Indian isolates (except for DNA-R and DNA-C component where they are closer to Egyptian isolates than Indian). The previous phylogenetic analyses from Pakistan based on Neighbor-Joining phylogenetic analysis of DNA-R only however, indicated that Pakistani isolates were closer to Egyptian isolates [25, 37]. Based on more rigorous Maximum Likelihood phylogenetic analyses of entire as well as other genomic components (i.e. DNA-U3, -S, -M and -N) it is evident that Pakistani isolates are closely related to Indian isolates than rest of any country in the PIO group.

The tendency of BBTV to induce genetic diversity is perhaps relevant for their ecological fitness. Selection pressure is an important estimator of evolutionary constraints imposed on coding regions [67]. The dN/dS ratio for coding regions (Table 5) of BBTV components showed that the population of DNA-R, DNA-S, DNA-M, DNA-C, and DNA-N components have strong purifying/negative selection while DNA-U3 have either positive or negative selection, which corroborates with the results of earlier studies for component R and S [30, 64]. In a comparative susceptibility study between two banana cultivars (Dwarf Brazilian AAB and Williams AAA) to BBTV, Dwarf Brazilian showed lower percentage of BBTV infection (39%) as compared to Williams (79%) in field experiments [68]. Based on their observation, Hooks and colleagues (2009) hypothesize that one or more morphological differences between the two cultivars might impact *P*. *nigronervosa* ability to inoculate BBTV. Banana pseudostem is waxy and banana aphid prefers to feed in pesudostem so the differences in wax content or composition between the two cultivars may lead to observed differences in virus transmission in their studied varieties. Furuya and colleagues (2012) [69] in a susceptibility study between Dwarf Cavendish (AAA) and Itobasho (BB) also observed reduced virus transmission in itobasho variety. Our analysis exhibited an excess of synonymous over nonsynonymous substitutions, indicating strong purifying (negative) selection as an additional mechanism constraining genetic variation [70]. BBTV is disseminated by aphid in a persistent manner, hence it is admissible that the respective constraint is inflicted on the CP gene to circumvent the accumulation of deleterious mutations which might be able to impede the virus-vector complex interactions [64, 71]. Majority of substitutions in DNA S were synonymous. As the sequence of DNA-S is conserved among all areas, the role of CP is crucial for the endurance of virus in the host plant [30]. Another possible sign of nucleotide encoding protein sequence having an impact on recombination patterns in BBTV is that the DNA-U3 component, which has no confirmed protein coding function, has a higher concentration of detectable recombination breakpoints (S2 and S3 Tables, Fig 3) than those of the known protein coding genes of other components. Remarkably, BBTV DNA-U3 seems to be most frequently exchanged by reassortment [31]. The presence of higher frequencies in the respective components depicts that it is substantially evolving neutrally without any risk that the recombinants might express defective chimeric proteins [72, 80]. Therefore, there is little conservation of coevolved epistatic interactions within the component. In comparison to the full-length sequences, low genetic diversity was observed in the coding regions (Table 6) delineating that recombination is not only confined to coding regions but it also proliferates to CR-M and CR-SL (Fig 3) in BBTV.

To evaluate natural selection at the population level significant negative values of neutrality test in different BBTV components (Table 6) suggest population expansion that is consistent with the previous studies for BBTV components R and S [30]. This suggests that whenever a population is increasing the number of segregating sites will increase more rapidly than that of nucleotide diversity leading to a negative test value. Positive values in few populations of BBTV components indicate that during balancing selection, alleles are kept at intermediate frequencies because there will be more pairwise differences than segregating sites [73]. This is due to considerable sequence heterogeneity that imparts a reservoir of virus variants in the population. It enables a significant adaptation to the changing environmental conditions. Therefore, the gene flow provided by the recombination exploit the mechanism to ameliorate their evolutionary tendencies and local adaptation.

Recombination is a major contributor to genetic diversity [18, 74] and genetic variations [75] in ssDNA viruses. Recombination analysis of BBTV components based on nucleotide sequence showed that DNA-U3 exhibits complex intra- and inter recombination patterns (Fig 3) with a recombination occurrence (Table 7) of about 16.8% which is about 9 times more than the least recombined DNA-S component (1.8%). While the recombination occurrence for other BBTV components ranged from 2.6%-7.92%. These observations suggest that recombination in components such as DNA-U3 may be selectively more favorable than recombination in components such as DNA-R, -S, -M, -C and -N [31]. DNA-R and DNA-S showed more frequent intergenomic recombination than intragenomic with a percentage of 1.73 and 1.45 respectively. Few incidences of recombination in DNA-R and DNA-S suggest that because of conserved nature and core functions [16, 64, 76] they are more prone to intergenomic rather than intragenomic recombination.

The contribution of genetic diversity by recombination (Table 8) revealed by analyzing the recombined and without recombination regions for each recombination event detected in RDP. The analysis showed that overall, on average there was about a 56.25% (27 populations out of 48 populations) increase in genetic diversity due to recombination. While within the BBTV components DNA-N (population of Tonga) and DNA-U3 (population of Australia, Tonga, China, Philippines and Congo) showed on average 100% and 90% increased diversity of recombinant populations. which indicates that there is a possibility that with component reassortment, recombination is also a significant evolutionary process driving the diversification of BBTV [30, 64, 76–78].

The frequency of recombined regions in BBTV genome undergoing recombination (Fig 3) involved as many as seven times in intergenomic, while as many as four times in intragenomic recombination in various isolates of BBTV. DNA-U3 showed higher frequencies of recombined regions than other components of BBTV and overall recombined regions reside mostly in the CR-SL region. This region is a recombination hot spot in members of the *geminiviridae* [12, 20, 72] due to the production of a nick in the stem-loop by Rep which serves as the initiation site for rolling circle replication [79] through host DNA polymerase. Since BBTV also replicates by a rolling-circle mechanism [14], the CR-SL of DNA-U3 may similarly be a region subject to high levels of recombination.

Conclusively, five full genomes have been sequenced from BBTV infected banana plants from Pakistan. Deep insight into the genetic diversity analysis of Pakistani and entire BBTV populations around the world showed some interesting contradictions in the diversity of functional regions which might be attributable to recombination. This study highlights the benefits of characterizing complete BBTV genomes rather than focusing on individual components from Pakistan. This analysis also complements the facts that different BBTV genomic components are diversifying at different rates and within one component, different parts have different levels of divergence. These differences suggest that geographic factors are also playing role in shaping the diversity and evolution of BBTV. Recombination analysis showed DNA-U3 a recombination hot component while CR-SL a recombination hotspot in BBTV genome. This in certain cases leads towards about four-fold increase in genetic diversity in the recombined population.

## Supporting information

S1 Table Abbreviations, accession numbers and geographical origin of Banana bunchy top virus isolates and their components used in the study. (DOCX)

S2 Table Intergenomic recombination in Banana bunchy top virus genomes. (DOCX)

S3 Table Intragenomic recombination in Banana bunchy top virus genomes. (DOCX)

S1 Fig Phylogenetic analysis of BBTV DNA-R illustrating intra and intergenomic recombination events at different nodes. (TIF)

S2 Fig Phylogenetic analysis of BBTV DNA-S illustrating intra and intergenomic recombination events at different nodes. (TIF)

S3 Fig Phylogenetic analysis of BBTV DNA-M illustrating intra and intergenomic recombination events at different nodes. (TIF)

S4 Fig Phylogenetic analysis of BBTV DNA-C illustrating intra and intergenomic recombination events at different nodes. (TIF)

S5 Fig Phylogenetic analysis of BBTV DNA-N illustrating intra and intergenomic recombination events at different nodes. (TIF)
