# Supplementary material for: Banana bunchy top virus genetic diversity in Pakistan and association of diversity with recombination in its genomes
Source: PLoS One. 2022 Mar 7;17(3):e0263875. doi: 10.1371/journal.pone.0263875 (PMC8901069; doi:10.1371/journal.pone.0263875)
Supplement: S1 Table — (DOCX) [file pone.0263875.s001.docx]

| **S1 Table: Abbreviations, accession numbers and geographical origin of Banana bunchy top virus isolates and their components used in the study.** | | | | | | | | | | |
| --- | --- | --- | --- | --- | --- | --- | --- | --- | --- | --- |
| **Geographical origin** | | | |  | **Accession Numbers** | | | | | |
| **Isolate** | **Region** | **Country** | **Subgroup** |  | **DNA-R** | **DNA-U3** | **DNA-S** | **DNA-M** | **DNA-C** | **DNA-N** |
| A.AUS | - | Australia | South Pacific |  | NC_003479 (S56276) | NC_003475 (L41576) | NC_003473 (L41574) | NC_003474 (L41575) | NC_003477 (L41578) | NC_003476 (L41577) |
| A.B2817 | The Channon | Australia | South pacific |  | KM607614 | KM607749 | KM607472 | KM607178 | KM607035 | KM607325 |
| A.B2818 | The Channon | Australia | South pacific |  | KM607615 | KM607750 | - | KM607179 | KM607036 | KM607326 |
| A.B2819 | The Channon | Australia | South pacific |  | KM607616 | KM607751 | KM607473 | KM607180 | KM607037 | KM607327 |
| A.B2820 | The Channon | Australia | South pacific |  | KM607617 | - | KM607474 | KM607181 | KM607038 | - |
| A.B2821 | The Channon | Australia | South pacific |  | KM607618 | KM607752 | KM607475 | KM607182 | KM607039 | KM607328 |
| A.B2822 | The Channon | Australia | South pacific |  | KM607619 | KM607753 | KM607476 | KM607183 | KM607040 | KM607329 |
| A.B2823 | The Channon | Australia | South pacific |  | KM607620 | KM607754 | KM607477 | - | KM607041 | KM607330 |
| A.B2824 | The Channon | Australia | South pacific |  | KM607621 | KM607755 | KM607478 | KM607184 | KM607042 | KM607331 |
| A.B2825 | The Channon | Australia | South pacific |  | KM607622 | KM607756 | KM607479 | KM607185 | KM607043 | KM607332 |
| A.B2826 | The Channon | Australia | South Pacific |  | KM607623 | - | KM607480 | KM607186 | - | KM607333 |
| A.B2827 | The Channon | Australia | South Pacific |  | KM607624 | KM607757 | KM607481 | - | KM607044 | - |
| A.B2828 | The Channon | Australia | South Pacific |  | KM607625 | - | KM607482 | KM607187 | KM607045 | - |
| A.B2829 | The Channon | Australia | South Pacific |  | KM607626 | KM607758 | KM607483 | KM607188 | KM607046 | KM607334 |
| A.B2830 | The Channon | Australia | South Pacific |  | - | KM607759 | KM607484 | KM607189 | KM607047 | KM607335 |
| A.B2832 | Tweed valley | Australia | South Pacific |  | KM607627 | - | KM607485 | KM607190 | KM607048 | - |
| A.B2833 | Tweed valley | Australia | South Pacific |  | KM607628 | KM607760 | - | KM607191 | KM607049 | KM607336 |
| A.B2834 | Tweed valley | Australia | South Pacific |  | KM607629 | KM607761 | KM607486 | KM607192 | - | KM607337 |
| A.B2844 | Flaxton | Australia | South Pacific |  | KM607630 | - | - | - | KM607050 | KM607338 |
| A.B2845 | Flaxton | Australia | South Pacific |  | KM607631 | KM607762 | KM607487 | KM607193 | KM607051 | KM607339 |
| A.B2846 | Yandina | Australia | South Pacific |  | KM607632 | - | KM607488 | KM607194 | KM607052 | KM607340 |
| A.B2847 | Yandina | Australia | South pacific |  | KM607633 | KM607763 | KM607489 | KM607195 | KM607053 | KM607341 |
| A.KP14 | Burringbah | Australia | South Pacific |  | KM607650 | KM607780 | KM607506 | KM607212 | KM607070 | KM607358 |
| A.KP15 | Burringbah | Australia | South Pacific |  | KM607651 | KM607781 | KM607507 | KM607213 | KM607071 | KM607359 |
| A.KP16 | Burringbah | Australia | South Pacific |  | KM607652 | KM607782 | KM607508 | KM607214 | KM607072 | KM607360 |
| A.KP17 | Burringbah | Australia | South Pacific |  | KM607653 | KM607783 | KM607509 | KM607215 | KM607073 | KM607361 |
| A.KP18 | Burringbah | Australia | South Pacific |  | KM607654 | KM607784 | KM607510 | KM607216 | KM607074 | KM607362 |
| A.KP6 |  | Australia | South Pacific |  | KM607657 | KM607786 | KM607513 | KM607219 | KM607077 | KM607365 |
| A.KP7 | Currumbin | Australia | South Pacific |  | KM607658 | - | - | KM607220 | KM607078 | - |
| A.KP8 | Currumbin | Australia | South Pacific |  | KM607659 | - | - | KM607221 | KM607079 | KM607366 |
| A-1429B | - | Australia | South Pacific |  | KM607585 | - | - | KM607149 | KM607006 | KM607295 |
| A.1429A | - | Australia | South Pacific |  | - | - | KM607439 | KM607148 | KM607005 | KM607294 |
| A.1900A | - | Australia | South Pacific |  | KM607586 | KM607722 | KM607440 | KM607150 | KM607007 | KM607296 |
| A.1900B | - | Australia | South Pacific |  | KM607587 | KM607723 | KM607441 | KM607151 | KM607008 | KM607297 |
| A.2557 | - | Australia | South Pacific |  | KM607588 | KM607724 | KM607442 | KM607152 | KM607009 | KM607298 |
| A.482_96 | - | Australia | South Pacific |  | KM607589 | KM607725 | KM607443 | KM607153 | KM607010 | KM607299 |
| A.482_97 | - | Australia | South Pacific |  | KM607590 | KM607726 | KM607444 | KM607154 | KM607011 | KM607300 |
| A.482_98 | - | Australia | South Pacific |  | KM607591 | KM607727 | KM607445 | KM607155 | KM607012 | KM607301 |
| A.482P2 | - | Australia | South Pacific |  | KM607592 | - | - | - | - | - |
| A.602 | - | Australia | South Pacific |  | - | KM607741 | KM607460 | KM607170 | KM607026 | KM607315 |
| A.737 | Pimpama | Australia | South Pacific |  | - | - | KM607467 | KM607173 | KM607030 | KM607321 |
| B.526 | - | Burundi | South Pacific |  | KM607598 | KM607732 | KM607451 | KM607161 | KM607017 | KM607306 |
| B.547 | - | Burundi | South Pacific |  |  | KM607735 | KM607454 | KM607164 | KM607020 | KM607309 |
| B.548 | - | Burundi | South Pacific |  | KM607601 | KM607736 | KM607455 | KM607165 | KM607021 | KM607310 |
| B.549 | - | Burundi | South Pacific |  | KM607602 | KM607737 | KM607456 | KM607166 | KM607022 | KM607311 |
| B.BUR | - | Burundi | South Pacific |  | - | - | AF148943 | - | - | - |
| Cam.TV14.1 | - | Cameroon | South Pacific |  | - | - | JF755979 | - | - | - |
| Cam.TV4.1 | - | Cameroon | South Pacific |  | - | - | JF755978 | - | - | - |
| CAME | - | Cameroon | South Pacific |  | - | - | GQ249344 | - | - | - |
| C.C4 | - | China | Asian |  | U97525 | - | - | - | - | - |
| C.CMH | - | China | Asian |  | - | GU559702 | - | - | - | - |
| C.DW4 | Guangdong | China | Asian |  | - | KX783438 | KX779465 | KX779455 | KX779460 | KX787074 |
| C.DZH | - | China | Asian |  | - | GU559705 | - | - | - | - |
| C.DZHD | - | China | Asian |  | - | GU559706 | - | - | - | - |
| C.HAIN | Hainan | China | Asian |  | AY450396 | AY606084 | AY494786 | AY494788 | AY606085 | AY494787 |
| C.HF-1 | Hainan | China | Asian |  |  | KX783437 | KX779466 | KX779456 | KX779461 | KX787073 |
| C.HKU | Haikou | China | Asian |  | FJ463042 | FJ463043 | FJ463044 | FJ463045 | FJ463046 | FJ463047 |
| C.HKU1 | Haikou | China | Asian |  | - | GU559704 | - | - | - | - |
| C.HKU2 | Haikou | China | Asian |  | HQ616074 | HQ616075 | HQ616076 | HQ616077 | HQ616078 | HQ616079 |
| C.HKU3 | Haikou | China | Asian |  | - | HM212635 | - | - | - | - |
| C.HKU4 | Haikou | China | Asian |  | HQ378190 | HM231314 | HQ378191 | HQ378192 | HQ378193 | HQ378194 |
| C.HS-5 | Hainan | China | Asian |  | - | KX783436 | KX779467 | KX779457 | KX779462 | KX787072 |
| C.LDH | Haikou | China | Asian |  | - | GU559703 | - | - | - | - |
| C.NS | Guangdong | China | Asian |  | - | - | - | - | - | AF238878 |
| C.NSP | Guangdong | China | Asian |  | - | - | - | - | - | AF238879 |
| C.NSP2 | - | China | Asian |  | - | - | - | - | - | EF470243 |
| C.Q529_1 | - | China | Asian |  | KM607676 | - | KM607533 | - | - | KM607385 |
| C.Q529_2 | - | China | Asian |  | KM607677 | - | KM607534 | - | KM607098 | KM607386 |
|  | - | - | South Pacific |  | - | KM607806 | - | - | - | - |
| C.Q529_4 | - | China | Asian |  | KM607678 | - | KM607535 | KM607239 | KM607099 | KM607387 |
|  | - | - | South Pacific |  | - | KM607807 | - | - | - | - |
| C.Q529_6 | - | China | Asian |  | KM607679 | - | KM607536 | KM607240 | - | - |
|  | - | - | South Pacific |  | - | KM607808 | - | - | - | - |
| C.XP-1 | Guangxi | China | Asian |  | - | KX783435 | KX779468 | KX779458 | KX779463 | KX787070 |
| C.XTD | Guangxi | China | Asian |  | - | KX783434 | KX779469 | KX779459 | KX779464 | KX787071 |
| C.ZANG | Zhangzhou | China | Asian |  | - | - | - | AF349568 | - | - |
| Co.550 | Brazzaville | Congo | South Pacific |  | KM607603 | KM607738 | KM607457 | KM607167 | KM607023 | KM607312 |
| Co.Bit-20 | - | Congo | South Pacific |  | KU687046 | - | - | - | - | - |
| Co.Bit-21 | - | Congo | South Pacific |  | KU687047 | - | - | - | - | - |
| Co.Bit-26 | - | Congo | South Pacific |  | KU687052 | - | - | - | - | - |
| Co.Bit-29 | - | Congo | South Pacific |  | KU687055 | - | - | - | - | - |
| Co.Bit-33 | - | Congo | South Pacific |  | KU687058 | - | - | - | - | - |
| Co.Bit-38 | - | Congo | South Pacific |  | KU687063 | - | - | - | - | - |
| Co.Bita-52 | - | Congo | South Pacific |  | KU687072 | - | - | - | - | - |
| Co.Bmul-63 | - | Congo | South Pacific |  | KU687077 | - | - | - | - | - |
| Co.Bmul-65 | - | Congo | South Pacific |  | KU687079 | - | - | - | - | - |
| Co.Bmul-70 | - | Congo | South Pacific |  | KU687081 | - | - | - | - | - |
| Co.Bmul-77 | - | Congo | South Pacific |  | KU687083 | KU759871 | KU759885 | KU759886 | KU759887 | KU759888 |
| Co.BU1 | - | Congo | South Pacific |  | KM607634 | KM607764 | KM607490 | KM607196 | KM607054 | KM607342 |
| Co.BU10 | - | Congo | South Pacific |  | KM607635 | KM607765 | KM607491 | KM607197 | KM607055 | KM607343 |
| Co.BU11 | - | Congo | South Pacific |  | KM607636 | KM607766 | KM607492 | KM607198 | KM607056 | KM607344 |
| Co.BU12 | - | Congo | South Pacific |  | KM607637 | KM607767 | KM607493 | KM607199 | KM607057 | KM607345 |
| Co.BU13 | - | Congo | South Pacific |  | KM607638 | KM607768 | KM607494 | KM607200 | KM607058 | KM607346 |
| Co.BU14 | - | Congo | South Pacific |  | KM607639 | KM607769 | KM607495 | KM607201 | KM607059 | KM607347 |
| Co.BU15 | - | Congo | South Pacific |  | KM607640 | KM607770 | KM607496 | KM607202 | KM607060 | KM607348 |
| Co.BU16 | - | Congo | South Pacific |  | KM607641 | KM607771 | KM607497 | KM607203 | KM607061 | KM607349 |
| Co.BU17 | - | Congo | South Pacific |  | KM607642 | KM607772 | KM607498 | KM607204 | KM607062 | KM607350 |
| Co.BU18 | - | Congo | South Pacific |  | KM607643 | KM607773 | KM607499 | KM607205 | KM607063 | KM607351 |
| Co.BU19 | - | Congo | South Pacific |  | KM607644 | KM607774 | KM607500 | KM607206 | KM607064 | KM607352 |
| Co.BU2 | - | Congo | South Pacific |  | KM607645 | KM607775 | KM607501 | KM607207 | KM607065 | KM607353 |
| Co.BU20 | - | Congo | South Pacific |  | KM607646 | KM607776 | KM607502 | KM607208 | KM607066 | KM607354 |
| Co.BU6 | - | Congo | South Pacific |  | KM607647 | KM607777 | KM607503 | KM607209 | KM607067 | KM607355 |
| Co.BU7 | - | Congo | South Pacific |  | KM607648 | KM607778 | KM607504 | KM607210 | KM607068 | KM607356 |
| Co.BU9 | - | Congo | South Pacific |  | KM607649 | KM607779 | KM607505 | KM607211 | KM607069 | KM607357 |
| Co.Bum-105 | - | Congo | South Pacific |  | KU687088 | - | - | - | - | - |
| Co.Kase-111 | - | Congo | South Pacific |  | KU687090 | - | - | - | - | - |
| Co.Kbd-66 | - | Congo | South Pacific |  | KU687080 | - | - | - | - | - |
| Co.Kim-86 | - | Congo | South Pacific |  | KU687087 | - | - | - | - | - |
| Co.Kip-15 | - | Congo | South Pacific |  | KU687042 | - | - | - | - | - |
| Co.Kip-16 | - | Congo | South Pacific |  | KU687043 | - | - | - | - | - |
| Co.Kiy-106 | - | Congo | South Pacific |  | KU687089 | - | - | - | - | - |
| Co.Kwd-64 | - | Congo | South Pacific |  | KU687078 | - | - | - | - | - |
| Co.Kwg-19 | - | Congo | South Pacific |  | KU687045 | - | - | - | - | - |
| Co.Kwg-27 | - | Congo | South Pacific |  | KU687053 | - | - | - | - | - |
| Co.Kwg-28 | - | Congo | South Pacific |  | KU687054 | - | - | - | - | - |
| Co.Kwg-31 | - | Congo | South Pacific |  | KU687057 | KU759870 | KU759881 | KU759882 | KU759883 | KU759884 |
| Co.Kwg-36 | - | Congo | South Pacific |  | KU687061 | - | - | - | - | - |
| Co.Kwg-37 | - | Congo | South Pacific |  | KU687062 | - | - | - | - | - |
| Co.Kwg-41 | - | Congo | South Pacific |  | KU687065 | - | - | - | - | - |
| Co.Kwg-49 | - | Congo | South Pacific |  | KU687069 | - | - | - | - | - |
| Co.Kwg-50 | - | Congo | South Pacific |  | KU687070 | - | - | - | - | - |
| Co.Kwg-51 | - | Congo | South Pacific |  | KU687071 | - | - | - | - | - |
| Co.Kwg-56 | - | Congo | South Pacific |  | KU687074 | - | - | - | - | - |
| Co.Kwg-58 | - | Congo | South Pacific |  | KU687075 | - | - | - | - | - |
| Co.Lubum-4 | - | Congo | South Pacific |  | KU687040 | - | - | - | - | - |
| Co.Lubum-9 | - | Congo | South Pacific |  | KU687041 | KU759868 | KU759873 | KU759874 | KU759875 | KU759876 |
| Co.Mbk-17 | - | Congo | South Pacific |  | KU687044 | - | - | - | - | - |
| Co.Mbk-23 | - | Congo | South Pacific |  | KU687049 | - | - | - | - | - |
| Co.Mbk-24 | - | Congo | South Pacific |  | KU687050 | KU759869 | KU759877 | KU759878 | KU759879 | KU759880 |
| Co.Mbk-34 | - | Congo | South Pacific |  | KU687059 | - | - | - | - | - |
| Co.Mbk-35 | - | Congo | South Pacific |  | KU687060 | - | - | - | - | - |
| Co.Mbk-44 | - | Congo | South Pacific |  | KU687066 | - | - | - | - | - |
| Co.Mbk-47 | - | Congo | South Pacific |  | KU687067 | - | - | - | - | - |
| Co.Menk-22 | - | Congo | South Pacific |  | KU687048 | - | - | - | - | - |
| Co.Menk-25 | - | Congo | South Pacific |  | KU687051 | - | - | - | - | - |
| Co.Menk-30 | - | Congo | South Pacific |  | KU687056 | - | - | - | - | - |
| Co.Menk-39 | - | Congo | South Pacific |  | KU687064 | - | - | - | - | - |
| Co.Menk-48 | - | Congo | South Pacific |  | KU687068 | - | - | - | - | - |
| Co.Menk-54 | - | Congo | South Pacific |  | KU687073 | - | - | - | - | - |
| Co.Menk-59 | - | Congo | South Pacific |  | KU687076 | - | - | - | - | - |
| Co.Mvz-80 | - | Congo | South Pacific |  | KU687084 | KU759872 | KU759889 | KU759890 | KU759891 | KU759892 |
| Co.Mvz-81 | - | Congo | South Pacific |  | KU687085 | - | - | - | - | - |
| Co.Mvz-82 | - | Congo | South Pacific |  | KU687086 | - | - | - | - | - |
| Co.Nsan | - | Congo | South Pacific |  | KU687091 | - | - | - | - | - |
| Co.Tshil-75 | - | Congo | South Pacific |  | KU687082 | - | - | - | - | - |
| DRC.TV23.3 | Canon | Congo | South Pacific |  | - | - | JF755986 | - | - | - |
| DRC.TV24.9 | Canon | Congo | South Pacific |  | - | - | JF755984 | - | - | - |
| DRC.TV25.2 | Canon | Congo | South Pacific |  | - | - | JF755987 | - | - | - |
| E.1 | - | Egypt | South Pacific |  | LC468138 | LC468139 | LC468140 | LC468141 | LC468142 | LC468143 |
| E.8 | - | Egypt | Asian |  | KM607612 | - | - | KM607176 | KM607033 | - |
|  | - | - | South Pacific |  | - | KM607747 | KM607470 | - | - | KM607324 |
| E.9 | - | Egypt | South Pacific |  | KM607613 | KM607748 | KM607471 | KM607177 | KM607034 | - |
| E.EDRSA-1991 | - | Egypt | South Pacific |  | - | LC155097 | - | - | - | - |
| E.EGY | - | Egypt | South Pacific |  | AF416465 | - | - | - | - | - |
| E.EGY1 | - | Egypt | South Pacific |  | HQ259074 | - | - | - | - | - |
| E.KAL | Kalubia | Egypt | South Pacific |  | AF102780 | - | - | AF102783 | - | - |
| F.FIJ | - | Fiji | South Pacific |  | AF416466 | - | AF148944 | - | - | - |
| G.TV17.5 | - | Gabon | South Pacific |  | - | - | JF755982 | - | - | - |
| G.TV18.2 | - | Gabon | South Pacific |  | - | - | JF755981 | - | - | - |
| I.1 | - | India | South Pacific |  | KU559329 | KU559330 | KU559331 | KU559332 | KU559333 | KU559334 |
| I.MEG | - | India | South Pacific |  | JQ911667 | JQ911668 | - | - | - | - |
| I.UM | - | India | South Pacific |  | KC119098 | KC466373 | KC466374 | KC466375 | KC466376 | KC466377 |
| I.Palani hills | - | India | South Pacific |  | - | - | - | MN011924 | - | - |
| I.2 | - | India | South Pacific |  | - | - | - | MN011925 | - | - |
| I.523_6A | - | India | Asian |  | KM607596 | - | - | - | - | - |
|  | - | - | South Pacific |  | - | - | KM607449 | KM607159 | - | - |
| I.523_6B | - | India | Asian |  | KM607597 | - | KM607450 | - | KM607016 | KM607305 |
|  | - | - | South Pacific |  | - | KM607731 | - | KM607160 |  |  |
| I.736_4 | - | India | South Pacific |  | KM607609 | KM607745 | KM607466 | KM607172 | KM607029 | KM607320 |
| I.AR4-gn | Arunachal | India | South pacific |  | KP876490 | - | - | - | - | - |
| I.AR7-gc | Arunachal | India | South Pacific |  | KP876491 |  |  |  |  |  |
| I.As1 | - | India | South Pacific |  | KP876497 | - | - | - | - | - |
| I.AS-JOR-B12 | - | India | South Pacific |  | - | - | - | - | - | KX592200 |
| I.AS-JOR-B2 | - | India | South Pacific |  | KX868958 | - | - | - | - | - |
| I.AS-JOR-B3 | - | India | South Pacific |  | - | KX868959 | - | - | - | - |
| I.AS-JOR-B5 | Jorhat Assam | India | South pacific |  | - | - | KX592198 | - | - | - |
| I.AS-JOR-B8 | Jorhat Assam | India | South pacific |  | - | - | - | KX592199 |  | - |
| I.AS-JOR-B9 | Jorhat Assam | India | South pacific |  | - | - | - | - | KX868960 | - |
| I.BBTR1 |  | India | South Pacific |  | - | AY884172 | - | - | - | - |
| I.BG | Bangalore | India | South Pacific |  | - | EU046323 | - | - | - | - |
| I.BGKVK | Bangalore | India | South Pacific |  | JN243751 | JN243752 | JN243753 | - | - | JN243754 |
| I.BH2 | Bihar | India | South Pacific |  | FJ605506 | FJ605508 | FJ605507 | FJ609642 | FJ609643 | FJ609644 |
| I.BRJT9 | - | India | South Pacific |  | - | AY884173 | - | - | - | - |
| I.BT1 | Hessaraghatta | India | South Pacific |  | - | AY960129 | - | AY948439 | - | AY948438 |
| I.DL | Delhi | India | South Pacific |  | HM120718 | - | - | - | - | - |
| I.ET | Etowah | India | South Pacific |  | DQ656119 | - | - | - | - | - |
| I.IND | - | India | South Pacific |  | AF416470 | - | - | - | - | - |
| I.IND2/I.TN4 | Tamil Nadu | India | South Pacific |  | EU140342 | EU140341 | EU589459 | EU190971 | EU190969 | EU190970 |
| I.KP | Kanpur | India | South Pacific |  | DQ656118 | - | - | - | - | - |
| I.KRL1 | Kerala | India | South Pacific |  | FJ009238 | FJ009239 | - | - | - | - |
| I.KRL2 | Kerala | India | South Pacific |  | FJ009240 | - | - | - | - | - |
| I.LK | Lucknow | India | South Pacific |  | DQ256267 | EU402601 | EF687856 | EU516323 | EU051379 | EU391633 |
| I.Manp6-gn | Manipur | India | South Pacific |  | KP876492 | - | - | - | - | - |
| I.Manp13-un | Manipur | India | South pacific |  | KP876494 | - | - | - | - | - |
| I.Manp9-gn | Manipur | India | South pacific |  | KP876493 | - | - | - | - | - |
| I.Mizo3-gc | Mizoram | India | Asian |  | KP876495 | - | - | - | - | - |
| I.Mizo4-sab | Mizoram | India | Asian |  | KP876496 | - | - | - | - | - |
| I.Q524_1 | - | India | South Pacific |  | KM607674 | KM607803 | KM607530 | KM607237 | KM607095 | KM607383 |
| I.Q524_2 | - | India | South Pacific |  | - | KM607804 | KM607531 | - | KM607096 |  |
| I.Q524_3 | - | India | South Pacific |  | KM607675 | KM607805 | KM607532 | KM607238 | KM607097 | KM607384 |
| I.SL | - | India | South Pacific |  | - | - | - | AY953429 | - | - |
| I.TN3 | Tamil Nadu | India | South Pacific |  | AY845437 | - | - | - | - | - |
| I.Tri8 | Tamil Nadu | India | South Pacific |  | - | - | KJ513017 | KJ513018 | KJ513019 | KJ513020 |
| I.Trp2 | - | India | South Pacific |  | KP876498 | - | - | - | - | - |
| I.Trp-DH4 | Tripura | India | South Pacific |  | KR350615 | - | KT180298 | - | - | - |
| I.Trp-DH6 | - | India | South Pacific |  | KR350616 | - | KT180299 | - | - | - |
| I.Trp-DH7 | - | India | South Pacific |  | KR350617 | - | KT180300 | - | - | - |
| I.Trp-DH8 | - | India | South Pacific |  | KR350618 | - | KT180301 | - | - | - |
| I.Trp-GO1 | Tripura | India | South Pacific |  | KR350592 | - | KT180275 | - | - | - |
| I.Trp-GO2 | Tripura | India | South Pacific |  | KR350593 | - | KT180276 | - | - | - |
| I.Trp-GO3 | Tripura | India | South Pacific |  | KR350594 | - | KT180277 | - | - | - |
| I.Trp-GO4 | Tripura | India | South Pacific |  | KR350595 | - | KT180278 | - | - | - |
| I.Trp-GO5 | Tripura | India | South Pacific |  | KR350596 | - | KT180279 | - | - | - |
| I.Trp-GO6 | Tripura | India | South Pacific |  | KR350597 | - | KT180280 | - | - | - |
| I.Trp-GO7 | Tripura | India | South Pacific |  | KR350598 | - | KT180281 | - | - | - |
| I.Trp-GO8 | Tripura | India | Asian |  | - | - | KT180282 | - | - | - |
|  | - | - | South Pacific |  | KR350599 | - | - | - | - | - |
| I.Trp-KH1 | Tripura | India | Asian |  | - | - | KT180283 | - | - | - |
|  | - | - | South Pacific |  | KR350600 | - | - | - | - | - |
| I.Trp-KH4 | Tripura | India | South Pacific |  | KR350601 | - | KT180284 | - | - | - |
| I.Trp-N3 | Tripura | India | Asian |  | - | - | KT180271 | - | - | - |
|  | - | - | South Pacific |  | KR350588 | - | - | - | - | - |
| I.Trp-N4 | Tripura | India | South Pacific |  | KR350589 | - | KT180272 | - | - | - |
| I.Trp-N5 | Tripura | India | Asian |  | - | - | KT180273 | - | - | - |
| - | - | - | South Pacific |  | KR350590 | - | - | - | - | - |
| I.Trp-N6 | Tripura | India | Asian |  | - | - | KT180274 | - | - | - |
|  | - | - | South Pacific |  | KR350591 | - | - | - | - | - |
| I.Trp-SH2 | Tripura | India | South Pacific |  | KR350606 | - | KT180289 | - | - | - |
| I.Trp-SH3 | Tripura | India | South Pacific |  | KR350607 | - | KT180290 | - | - | - |
| I.Trp-SH4 | Tripura | India | South Pacific |  | KR350608 | - | KT180291 | - | - | - |
| I.Trp-SH8 | Tripura | India | South Pacific |  | KR350609 | - | KT180292 | - | - | - |
| I.Trp-DH2 | Tripura | India | South Pacific |  | KR350614 | - | KT180297 | - | - | - |
| I.Trp-SO2 | Tripura | India | South Pacific |  | KR350602 | - | KT180285 | - | - | - |
| I.Trp-SO3 | Tripura | India | South Pacific |  | KR350603 | - | KT180286 | - | - | - |
| I.Trp-SO5 | Tripura | India | South Pacific |  | KR350604 | - | KT180287 | - | - | - |
| I.Trp-SO6 | Tripura | India | South Pacific |  | KR350605 | - | KT180288 | - | - | - |
| I.Trp-W1 | Tripura | India | South Pacific |  | KR350610 | - | KT180293 | - | - | - |
| I.Trp-W2 | Tripura | India | South Pacific |  | KR350611 | - | KT180294 | - | - | - |
| I.Trp-W7 | Tripura | India | South Pacific |  | KR350612 | - | KT180295 | - | - | - |
| I.Trp-W8 | Tripura | India | South Pacific |  | KR350613 | - | KT180296 | - | - | - |
| I.AR1 | Arunachal | India | South Pacific |  | KP876489 | - | - | - | - | - |
| In.520 | - | Indonesia | Asian |  | KM607593 | KM607728 | KM607446 | KM607156 | KM607013 | KM607302 |
| In.Bali5 | Gianyar | Indonesia | Asian |  | - | - | LC481518 | - | - | - |
| In.Bali2 | Denpasar | Indonesia | Asian |  | - | - | LC481517 | - | - | - |
| In.BSI | Bali Sempidi | Indonesia | Asian |  | JN003633 | - | - | - | - | - |
| In.BTII | - | Indonesia | Asian |  | JN003631 | - | - | - | - | - |
| In.BTPI | Bali TukadPetanu | Indonesia | Asian |  | JN003632 | - | - | - | - | - |
| In.GM_109001 | - | Indonesia | Asian |  | MK940788 | - | - | - | - | - |
| In.GM_109003 | - | Indonesia | Asian |  | MK940789 | - | - | - | - | - |
| In.GM_112906 | - | Indonesia | Asian |  | MN037872 | - | - | - | - | - |
| In.GM_619001 | - | Indonesia | Asian |  | MN037873 | - | - | - | - | - |
| In.GM_519006 | - | Indonesia | Asian |  | MN037874 | - | - | - | - | - |
| In.GM_519005 | - | Indonesia | Asian |  | MN037875 | - | - | - | - | - |
| In.GM_418004 | - | Indonesia | Asian |  | MN037876 | - | - | - | - | - |
| In.GM_418002 | - | Indonesia | Asian |  | MN037877 | - | - | - | - | - |
| In.GM_212004 | - | Indonesia | Asian |  | MN037878 | - | - | - | - | - |
| In.GM_214020 | - | Indonesia | Asian |  | MK805529 | - | - | - | - | - |
| In.IG33 | - | Indonesia | Asian |  | AB186924 | - | AB186927 | - | - | - |
| In.IG64 | - | Indonesia | Asian |  | AB186925 | - | AB186928 | - | - | - |
| In.IJS11 | - | Indonesia | Asian |  | AB186926 | - | AB186929 | - | - | - |
| In.Q568_1 | - | Indonesia | Asian |  | KM607681 | KM607810 |  | KM607242 | KM607101 | KM607389 |
| In.Q568_3 | - | Indonesia | Asian |  | KM607682 | KM607811 | KM607538 | KM607243 | KM607102 | KM607390 |
| J.JK3 | Okinawa | Japan | Asian |  | AB108453 | - | AB108450 | - | - | - |
| J.JM5 | - | Japan | Asian |  | AB108454 | - | - | - | - | - |
| J.JM6 | - | Japan | Asina |  | AB108455 | - | - | - | - | - |
| J.JN4 | Okinawa | Japan | Asian |  | AB108452 | - | AB108449 | - | - | - |
| J.JY1 | Okinawa | Japan | Asian |  | AB108456 | - | AB108451 | - | - | - |
| J.JY3 | Taketomi | Japan | Asian |  | AB108457 | - | - | - | - | - |
| J.JY7 | Iriomote | Japan | Asian |  | AB108458 | - | - | - | - | - |
| M.MY01 | - | Myanmar | South Pacific |  | AB252639 | - | AB252642 | - | - | - |
| M.MY02 | - | Myanmar | South Pacific |  | AB252640 | - | AB252643 | - | - | - |
| M.MY03 | - | Myanmar | South Pacific |  | AB252641 | - | AB252644 | - | - | - |
| Ma.MAL.TV5.4 | - | Malawi | South Pacific |  | - | - | JF755980 | - | - | - |
| Ma.MAL73 | - | Malawi | South Pacific |  | JQ820453 | JQ820454 | JQ820455 | JQ820456 | JQ820457 | JQ820458 |
| P.BS1 | Bhitshah | Pakistan | South Pacific |  | FJ859727 | - | FJ859740 | JX467685 | - | - |
| P.BS2 | Bhitshah | Pakistan | South Pacific |  | FJ859728 | - | FJ859741 | - | - | - |
| P.CM | Chamber | Pakistan | South Pacific |  | AM418537 | - | - | - | - | - |
| P.GH1 | Ghotki | Pakistan | South Pacific |  | FJ859722 | FJ859748 | FJ859735 | - | - | - |
| P.HD1 | Hyderabad | Pakistan | South Pacific |  | FJ859733 | FJ859750 | FJ859746 | -JX467686 | - | - |
| P.HD2 | Hyderabad | Pakistan | South Pacific |  | FJ859734 | - | FJ859747 | - | - | - |
| P.HL | Hala | Pakistan | South Pacific |  | AM418539 | - | - | - | - | - |
| P.JS1 | Jamshoro | Pakistan | South Pacific |  | FJ859732 | FJ859749 | FJ859745 | - | - | - |
| P.KHI | Karachi | Pakistan | South Pacific |  | - | AY996563 | - | - | - | - |
| P.KM | Kisanamari | Pakistan | South Pacific |  | - | - | AM418565 | - | - | - |
| P.KP1 | Khairpur | Pakistan | South Pacific |  | FJ859723 | - | FJ859736 | - | - | - |
| P.KP2 | Khairpur | Pakistan | South Pacific |  | FJ859724 | - | FJ859737 | - | - | - |
| P.MT1 | Matiari | Pakistan | South Pacific |  | FJ859729 | - | FJ859742 | - | - | - |
| P.MT2 | Matiari | Pakistan | South Pacific |  | FJ859730 | - | FJ859743 | - | - | - |
| P.NARC | Tandojam | Pakistan | South Pacific |  | MK140625 | MK140628 | MK140619 | MK140616 | MK140613 | MK140622 |
| P.NR | Nasarpur | Pakistan | South Pacific |  | - | - | AM418567 | - | - | - |
| P.NS | Nawabshah | Pakistan | South Pacific |  | AM418534 | - | - | - | - | - |
| P.NS1 | Nawabshah | Pakistan | South Pacific |  | FJ859731 | - | FJ859744 | JX467687- | - | - |
| P.Sakrand | Tandojam | Pakistan | South Pacific |  | MK140627 | MK140630 | MK140621 | MK140618 | MK140615 | MK140624 |
| P.SK | Sarkand | Pakistan | South Pacific |  | AM418535 | - | - | - | - | - |
| P.TA1 | Tandoadam | Pakistan | South Pacific |  | FJ859725 | - | FJ859738 | - | - | - |
| P.TA2 | Tandoadam | Pakistan | South Pacific |  | FJ859726 | - | FJ859739 | - | - | - |
| P.TH | Thatta | Pakistan | South Pacific |  | AM418538 | - | AM418566 | AM418541 | AM418569 | AM418568 |
| P.TJ1 | Tandojam | Pakistan | South Pacific |  | AY996562 | GQ214699 | EF593169 | EU095948 | EF520722 | EF529519 |
| P.TJ2 | Tandojam | Pakistan | South Pacific |  | AM418536 | - | AM418540 | - | AM418564 | - |
| P.TJ3 | Tandojam | Pakistan | South Pacific |  | JX170762 | JX170764 | JX170763 | JX170760 | JX170759 | JX170761 |
| P.TJ4 | Tandojam | Pakistan | South Pacific |  | MK140626 | MK140629 | MK140620 | MK140617 | MK140614 | MK140623 |
| Ph.522A | - | Philippine | Asian |  | KM607594 | KM607729 | KM607447 | KM607157 | KM607014 | KM607303 |
| Ph.522B | - | Philippine | Asian |  | KM607595 | KM607730 | KM607448 | KM607158 | KM607015 | KM607304 |
| Ph.571_1 | - | Philippine | Asian |  | KM607604 | KM607739 | KM607458 | KM607168 | KM607024 | KM607313 |
| Ph.571_2 | - | Philippine | Asian |  | KM607605 | KM607740 | KM607459 | KM607169 | KM607025 | KM607314 |
| Ph.768 | - | Philippine | Asian |  | KM607611 | - | KM607469 | KM607175 | KM607032 | KM607323 |
| Ph.MS14 | - | Philippine | Asian |  | - | KM607790 | KM607517 | KM607224 | KM607082 | - |
| Ph.MS16 | Los Banos | Philippine | Asian |  | KM607663 | KM607792 | KM607519 | KM607226 | KM607084 | KM607371 |
| Ph.MS17 | Los Banos | Philippine | Asian |  | KM607664 | KM607793 | KM607520 | KM607227 | KM607085 | KM607372 |
| Ph.MS18 | Los Banos | Philippine | Asian |  | KM607665 | KM607794 | KM607521 | KM607228 | KM607086 | KM607373 |
| Ph.MS6 | Los Banos | Philippine | Asian |  | KM607666 | KM607795 | KM607522 | KM607229 | KM607087 | KM607374 |
| Ph.MS7 | Los Banos | Philippine | Asian |  | KM607667 | KM607796 | - | KM607230 | KM607088 | KM607375 |
|  | - | - | South Pacific |  | - | - | KM607523 | - | - | - |
| Ph.PHI | - | Philippine | Asian |  | AF416469 | - | AF148068 | - | - | - |
| Ph-MS15 | Los Banos | Philippine | Asian |  | KM607662 | KM607791 | KM607518 | KM607225 | KM607083 | KM607370 |
| Ph.bP5 | - | Philippine | Asian |  | AB189067 | - | AB189068 | - | - | - |
| Ph.bP26 | - | Philippine | Asian |  | AB250955 | - | AB250958 | - | - | - |
| Ph.aP34 | - | Philippine | Asian |  | AB250954 | - | AB250957 | - | - | - |
| Ph.aP32 | - | Philippine | Asian |  | AB250953 | - | - | - | - | - |
|  | - | - | South Pacific |  | - | - | AB250956 | - | - | - |
| R.RW138 | - | Rwanda | South Pacific |  | JQ820459 | JQ820460 | JQ820461 | JQ820462 | JQ820463 | JQ820464 |
| R.RW142 | - | Rwanda | South Pacific |  | JQ820465 | JQ820466 | JQ820467 | JQ820468 | JQ820469 | JQ820470 |
| S.Q279 | - | Samoa | South Pacific |  | KM607672 | KM607801 | KM607528 | KM607235 | KM607093 | KM607380 |
| S.Q280 | - | Samoa | Asian |  | - | - | - | - | - | KM607381 |
| S.Q281 | - | Samoa | South Pacific |  | KM607673 | KM607802 | KM607529 | KM607236 | KM607094 | KM607382 |
| Sri.KP5 | - | Sri Lanka | South Pacific |  | KM607656 |  | KM607512 | KM607218 | KM607076 | KM607364 |
| Sri.Kandy | Kandy | Sri Lanka | South Pacific |  | JN250593 | JN250594 | JN250595 | JN250596 | JN250597 | JN250598 |
| Sri.Q553 | - | Sri Lanka | South Pacific |  | KM607680 | KM607809 | KM607537 | KM607241 | KM607100 | KM607388 |
| T.625 | - | Taiwan | Asian |  | KM607606 | KM607742 | KM607461 | - | KM607027 | KM607316 |
| T.625I | - | Taiwan | Asian |  | KM607607 | KM607743 | KM607462 | - | - | - |
|  | - | - | South Pacific |  | - | - | - | - | - | KM607317 |
| T.626 | - | Taiwan | Asian |  | - | - | KM607463 | - | - | - |
|  | - | - | South Pacific |  | - | - | - | - | - | KM607318 |
| T.626M | - | Taiwan | Asian |  | - | - | KM607464 | - | - | - |
| T.627 | - | Taiwan | Asian |  | KM607608 | - | KM607465 | KM607171 | KM607028 | KM607319 |
|  | - | - | South Pacific |  | - | KM607744 | - | - | - | - |
| T.765 | - | Taiwan | Asian |  | KM607610 | KM607746 | KM607468 | KM607174 | KM607031 | KM607322 |
| T.MP1 | - | Taiwan | Asian |  | - | KM607788 | KM607515 | - | - | KM607368 |
| T.MP2 | - | Taiwan | Asian |  | KM607661 | KM607789 | KM607516 | KM607223 | KM607081 | KM607369 |
| T.Q1160 | - | Taiwan | Asian |  | KM607668 | KM607797 | KM607524 | KM607231 | KM607089 | KM607376 |
| T.Q623 | - | Taiwan | Asian |  | KM607684 | KM607813 | KM607540 | KM607245 | KM607104 | KM607392 |
| T.Q624 | - | Taiwan | Asian |  | KM607685 | KM607814 | KM607541 | KM607246 | KM607105 | KM607393 |
| T.SP (Severe phenotype) | - | Taiwan | Asian |  | DQ826390 | DQ826391 | DQ826393 | DQ826394 | DQ826395 | DQ826396 |
|  | - | - | South Pacific |  | - | DQ826392 | - | - | - | - |
| T.TW3 | - | Taiwan | Asian |  | EU366169 | FJ773283 | EU366171 | EU366172 | EU366173 | - |
|  |  | - | South Pacific |  | - | EU366170 | - | - | - | - |
| T.V-1a | - | Taiwan | Asian |  | EF095161 | - | - | EF095165 | - | - |
|  | - | - | South Pacific |  | EF095162 | EF095163 | EF095164 | - | EF095166 | - |
| T.TAI | - | Taiwan | Asian |  | AF416468 | - | AF148942 | - | - | - |
| T.Chaiyaphum-TH16 | - | Thailand | South Pacific |  | - | MF039875 | - | - | - | - |
| T.TH16 | Nakhon Ratchasima | Thailand | South Pacific |  | - | MF039881 | - | - | - | - |
| T.TH16 | Nong Khai | Thailand | Asian |  | KY427063 | - | KY427064 | KY427061 | KY427060 | KY427062 |
|  | - | - | South Pacific |  | - | KY427065 | - | - | - | - |
| Th.Ubon | - | Thailand | Asian |  | - | - | - | KC581796 | - | - |
| To.536 | - | Tonga | South Pacific |  | KM607600 | KM607734 | KM607453 | KM607163 | KM607019 | KM607308 |
| To.KP4 | - | Tonga | South Pacific |  | KM607655 | KM607785 | KM607511 | KM607217 | KM607075 | KM607363 |
| To.Q276 | Tongatapu | Tonga | South Pacific |  | KM607669 | KM607798 | KM607525 | KM607232 | KM607090 | KM607377 |
| To.Q277 | Tongatapu | Tonga | South Pacific |  | KM607670 | KM607799 | KM607526 | KM607233 | KM607091 | KM607378 |
| To.Q278 | Tongatapu | Tonga | South Pacific |  | KM607671 | KM607800 | KM607527 | KM607234 | KM607092 | KM607379 |
| To.Q570 | - | Tonga | South Pacific |  | KM607683 | KM607812 | KM607539 | KM607244 | KM607103 | KM607391 |
| To.TO114 | - | Tonga | South Pacific |  | JF957625 | JF957637 | JF957649 | JF957661 | JF957673 | JF957685 |
| To.TO121 | - | Tonga | South Pacific |  | JF957626 | JF957638 | JF957650 | JF957662 | JF957674 | JF957686 |
| To.TO124 | - | Tonga | South Pacific |  | JF957627 | JF957639 | JF957651 | JF957663 | JF957675 | JF957687 |
| To.TO166 | - | Tonga | South Pacific |  | JF957628 | JF957640 | JF957652 | JF957664 | JF957676 | JF957688 |
| To.TO208 | - | Tonga | South Pacific |  | JF957629 | JF957641 | JF957653 | JF957665 | JF957677 | JF957689 |
| To.TO224 | - | Tonga | South Pacific |  | JF957630 | JF957642 | JF957654 | JF957666 | JF957678 | JF957690 |
| To.TO290 | - | Tonga | South Pacific |  | JF957631 | JF957643 | JF957655 | JF957667 | JF957679 | JF957691 |
| To.TO306 | - | Tonga | South Pacific |  | JF957632 | JF957644 | JF957656 | JF957668 | JF957680 | JF957692 |
| To.TO310 | - | Tonga | South Pacific |  | JF957633 | JF957645 | JF957657 | JF957669 | JF957681 | JF957693 |
| To.TO314 | - | Tonga | South Pacific |  | JF957634 | JF957646 | JF957658 | JF957670 | JF957682 | JF957694 |
| To.TON | - | Tonga | South Pacific |  | AF416467 | - | - | - | - | - |
| To.TOS12 | - | Tonga | South Pacific |  | JF957635 | JF957647 | JF957659 | JF957671 | JF957683 | JF957695 |
| To.TOS14 | - | Tonga | South Pacific |  |  | KM607815 | KM607542 | KM607247 | KM607106 | KM607394 |
| To.TOS15 | - | Tonga | South Pacific |  | KM607686 |  | KM607543 | KM607248 | KM607107 |  |
| To.TOS16 | Tongatapu | Tonga | South Pacific |  | KM607687 | KM607816 | KM607544 | KM607249 | KM607108 | KM607395 |
| To.TOS19 | Tongatapu | Tonga | South Pacific |  | KM607688 | KM607817 | KM607545 | KM607250 | - | KM607396 |
| To.TOS2 | Tongatapu | Tonga | South Pacific |  | KM607689 | KM607818 | KM607546 | KM607251 | KM607109 | KM607397 |
| To.TOS20 | Tongatapu | Tonga | South Pacific |  | KM607690 | KM607819 | KM607547 | KM607252 | KM607110 | KM607398 |
| To.TOS21 | Tongatapu | Tonga | Asian |  | - | - | - | KM607253 | - | - |
|  | - | - | South Pacific |  | KM607691 | KM607820 | KM607548 | - | KM607111 | KM607399 |
| To.TOS22 | Tongatapu | Tonga | South Pacific |  | KM607692 | KM607821 | KM607549 | KM607254 | KM607112 | KM607400 |
| To.TOS25 | Tongatapu | Tonga | South Pacific |  | KM607693 | KM607822 | KM607550 | KM607255 | KM607113 | KM607401 |
| To.TOS28 | Tongatapu | Tonga | South Pacific |  | JF957636 | JF957648 | JF957660 | JF957672 | JF957684 | JF957696 |
| To.TOS29 | Tongatapu | Tonga | South Pacific |  | KM607694 | KM607823 | KM607551 | KM607256 | KM607114 | KM607402 |
| To.TOS34 | Tongatapu | Tonga | South Pacific |  | KM607695 | KM607824 | KM607552 | - | - | - |
| To.TOS39 | Tongatapu | Tonga | South Pacific |  | KM607696 | KM607826 | KM607553 | KM607257 | KM607115 | KM607403 |
| To.TOS4 | Tongatapu | Tonga | South Pacific |  | KM607697 | KM607825 | - | KM607258 | KM607116 | KM607404 |
| To.TOS40 | Tongatapu | Tonga | South Pacific |  | KM607698 | - | - | KM607259 | KM607117 | KM607405 |
| To.TOS42 | Tongatapu | Tonga | South Pacific |  | KM607699 | KM607827 | KM607554 | KM607260 | KM607118 | KM607406 |
| To.TOS43 | Tongatapu | Tonga | South Pacific |  | - | KM607828 | KM607555 | KM607261 | KM607119 | - |
| To.TOS45 | Tongatapu | Tonga | South Pacific |  | KM607700 | - | KM607556 | - | - | - |
| To.TOS46 | Tongatapu | Tonga | South Pacific |  | KM607701 | - | KM607557 | - | - | - |
| To.TOS48 | Tongatapu | Tonga | South Pacific |  | KM607702 | KM607829 | KM607558 | KM607262 | KM607120 | KM607407 |
| To.TOS49 | Tongatapu | Tonga | South Pacific |  | KM607703 | KM607830 | - | KM607263 | KM607121 | KM607408 |
| To.TOS5 | Tongatapu | Tonga | South Pacific |  | KM607704 | - | KM607559 | KM607264 | - | KM607409 |
| To.TOS53 | - | Tonga | South Pacific |  | - | - | - | - | - | KM607410 |
| To.TOS55 | - | Tonga | South Pacific |  | - | KM607831 | KM607560 | KM607265 | KM607122 | KM607411 |
| To.TOS56 | Tongatapu | Tonga | South Pacific |  | KM607705 | KM607832 | KM607561 | KM607266 | KM607123 | KM607412 |
| To.TOS57 | Tongatapu | Tonga | South Pacific |  | KM607706 | - | - | KM607267 | KM607124 | KM607413 |
| To.TOS58 | Tongatapu | Tonga | South Pacific |  | - | KM607833 | KM607562 | KM607268 | KM607125 | KM607414 |
| To.TOS59 | Tongatapu | Tonga | South Pacific |  | - | KM607834 | KM607563 | KM607269 | KM607126 | - |
| To.TOS60 | Tongatapu | Tonga | South Pacific |  | KM607707 | KM607835 | KM607564 | KM607270 | KM607127 | KM607415 |
| To.TOS61 | Tongatapu | Tonga | South Pacific |  | - | KM607836 | KM607565 | - | KM607128 | KM607416 |
| To.TOS62 | Tongatapu | Tonga | South Pacific |  | KM607708 | KM607837 | KM607566 | KM607271 | KM607129 | - |
| To.TOS63A | Tongatapu | Tonga | South Pacific |  | KM607709 | KM607838 | KM607567 | KM607272 | KM607130 | KM607417 |
| To.TOS63B | Tongatapu | Tonga | South Pacific |  | - | KM607839 | KM607568 | KM607273 | KM607131 | KM607418 |
| To.TOS64 | Tongatapu | Tonga | South Pacific |  | - | KM607840 | KM607569 | KM607274 | KM607132 | KM607419 |
| To.TOS65 | Tongatapu | Tonga | South Pacific |  | KM607710 | KM607841 | KM607570 | KM607275 | KM607133 | KM607420 |
| To.TOS67 | - | Tonga | South Pacific |  | - | KM607842 | - | KM607276 | KM607134 | KM607421 |
| To.TOS68 | Tongatapu | Tonga | South Pacific |  | - | KM607843 | KM607571 | KM607277 | KM607135 | KM607422 |
| To.TOS69 | - | Tonga | South Pacific |  | - | KM607844 | - | KM607278 | KM607136 | - |
| To.TOS7 | Tongatapu | Tonga | South Pacific |  | KM607711 | - | - | - | - | KM607423 |
| To.TOS70 | Tongatapu | Tonga | South Pacific |  | - | - | KM607572 | KM607279 | KM607137 | - |
| To.TOS71 | Nambour | Tonga | South Pacific |  | - | KM607845 | KM607573 | KM607280 | KM607138 | KM607424 |
| To.TOS72 | - | Tonga | South Pacific |  | KM607712 | KM607846 | - | KM607281 | KM607139 | KM607425 |
| To.TOS74 | - | Tonga | South Pacific |  | - | KM607847 | - | - | - | KM607426 |
| To.TOS76 | - | Tonga | South Pacific |  | KM607713 | - | - | KM607282 | KM607140 | - |
| To.TOS77 | - | Tonga | South Pacific |  | KM607714 | - | - | - | - | - |
| To.TOS78 | - | Tonga | South Pacific |  | - | KM607848 | KM607574 | KM607283 | KM607141 | KM607427 |
| To.TOS79 | - | Tonga | South Pacific |  | - | KM607849 | - | - | - | - |
| To.TOS80 | - | Tonga | South Pacific |  | - | KM607850 | - | KM607284 | - | KM607428 |
| To.TOS82 | - | Tonga | South Pacific |  | - | KM607851 | KM607575 | KM607285 | KM607142 | KM607429 |
| To.TOS83 | - | Tonga | South Pacific |  | KM607715 | KM607852 | KM607576 | KM607286 | KM607143 | KM607430 |
| To.TOS85 | - | Tonga | South Pacific |  | KM607716 | KM607853 | KM607577 | KM607287 | - | KM607432 |
| To.TOS86 | - | Tonga | South Pacific |  | KM607717 | - | - | - | - | KM607431 |
| To.TOS87 | - | Tonga | South Pacific |  | - | KM607854 | KM607578 | KM607288 | KM607144 | KM607433 |
| To.TOS88 | - | Tonga | South Pacific |  | KM607718 | KM607855 | KM607579 | KM607289 | - | KM607434 |
| To.TOS89 | - | Tonga | South Pacific |  | - | KM607856 | KM607580 | KM607290 | - | KM607435 |
| To.TOS90 | - | Tonga | South Pacific |  | KM607719 | KM607857 | KM607581 | KM607291 | KM607145 | KM607436 |
| To.TOS91 | - | Tonga | South Pacific |  | KM607720 | KM607858 | KM607582 | KM607292 | KM607146 | KM607437 |
| To.TOS92 | Nambour | Tonga | South Pacific |  | - | - | KM607583 | - | - | - |
| To.TOS93 | - | Tonga | South Pacific |  | KM607721 | KM607859 | KM607584 | KM607293 | KM607147 | KM607438 |
| U.527 | Hawaii | USA | South Pacific |  | KM607599 | KM607733 | KM607452 | KM607162 | KM607018 | KM607307 |
| U.KP9 | Hawaii | USA | South Pacific |  | KM607660 | KM607787 | KM607514 | KM607222 | KM607080 | KM607367 |
| V.BMT | Buon Ma Thout Region | Vietnam | Asian |  | AF416476 | - | - | - | - | - |
| V.BN | Bac Ninh Region | Vietnam | Asian |  | AF416474 | - | - | - | - | - |
| V.DBP | Dien Bhien Phu Region | Vietnam | Asian |  | AF416473 | - | - | - | - | - |
| V.DN | Da Nang Region | Vietnam | Asian |  | AF416477 | - | - | - | - | - |
| V.HCM | Ho Chi Minh City Region | Vietnam | Asian |  | AF416478 | - | - | - | - | - |
| V.HUE | Hue Region | Vietnam | Asian |  | AF416475 | - | - | - | - | - |
| V.SL | Son La Region | Vietnam | Asian |  | AF416472 | - | - | - | - | - |
| V.V14 | North of Hanoi | Vietnam | Asian |  | AB113660 | - | AB113662 | - | - | - |
| V.V6 | Hanoi Region | Vietnam | Asian |  | AB113659 | - | AB113661 | - | - | - |
| V.YB | Yen Bai Region | Vietnam | Asian |  | AF416479 | - | - | - | - | - |
| **Note:** The sequences of full length BBTV genomic components were accessed from GenBank on April 05, 2020. For A.AUS isolate the original accession numbers are also given (in parenthesis) along with the GenBank reference accessions for BBTV type isolate. | | | | | | | | | | |
