# Supplementary material for: Banana bunchy top virus genetic diversity in Pakistan and association of diversity with recombination in its genomes
Source: PLoS One. 2022 Mar 7;17(3):e0263875. doi: 10.1371/journal.pone.0263875 (PMC8901069; doi:10.1371/journal.pone.0263875)
Supplement: S2 Table — (DOCX) [file pone.0263875.s002.docx]

| **S2 Table. Intergenomic recombination in Banana bunchy top virus genomes** | | | | | | |
| --- | --- | --- | --- | --- | --- | --- |
| **Component (event)** | **Recombinant^1^** | **Minor Parent^1^** | **Major Parent^1^** | **Position^2^ in alignment/in recombinant** | **Position of recombinant according to TJ1** | **Method^3^ of recombination detection with average probability values at 95% confidence** |
| **Intergenomic recombination among South Pacific subgroup** | | | | | | |
| DNA-R (1) | R-I.1 | Unknown | T.V-1a,P.TJ2, Indian isolates (Manp9-gn,Trp-GO8,Trp-SO6,Trp-SH3,SH8,LK,DL,JOR-B2,Trp-KH1,Trp-N3,N6,TN4,TN3)Ma.MAL73,E.EGY1,S.Q281,A.B2847,Sri.Kandy,Sri.KP5,B.548,F.FIJ, Congo isolates (BU16,Kwg-31,BU18,Kiy-106) Tonga isolates (TON,TOS65,TOS5,TO310,TOS39,TOS86,Q277 | 803-1110/797-1084 Around CR-SL | 898-1110,1-74 End of coding region including CR-M & CR-SL | Maxchi 1.43x10^-05^, SiScan 6.72x10^-8^, 3Seq 3.02x10^-3^ |
| DNA U3 (2) | U3-Sri.Kandy,U3-C.Q529_6,U3-I.BRJT9,I.Q524_1,I.Q524_3 | Burundi isolates, Indian (KRL1,BBTR1,BGKVK,736,523)Sri.Q553,Ma.MAL73, All Congo isolates except (BU1,BU17,BU7) | Tonga isolates | 1-752/1-669 Coding region &  CR-M | 144-624 Coding region & CR-M | RDP 1.93x10^-08^, GENECONV 9.48x10^-08^, Bootscan 9.50x10^-09^, Maxchi 3.21x10^-10^, Chimaera 3.93x10^-11^, SiScan 5.05x10^-12^, 3Seq 2.85x10^-21^ |
| DNA U3 (3) | Tonga Isolates TOS69,TO114,TOS28,TOS4,TOS58,TOS56,TOS61,TOS62,TO166,TOS71,TOS74,TOS83,TOS59) | Unknown | Mostly Tonga isolates, All AUS,USA & Samoa isolates, Indian isolates(TN4,Q524_3,Q529_6,Q524_1,BRHT9,Q524_2,BT1,BH2,BG,AS-JOR-B3), Egypt (E.1,E.8,EDDRSA,E.9),T.627,T.V-1a,Sri.Kandy | 947-1072/805-881 Around CR-SL | 736-802 Around CR-M | RDP 1.64x10^-13^, GENECONV 6.20x10^-12^, Bootscan 6.82x10^-12^, Maxchi 6.82x10^-12^, Chimaera 3.65x10^-6^, SiScan 3.97x10^-9^, 3Seq 1.14x10^-20^ |
| DNA U3 (4) | All Pak,Burundi, Congo, Malawai & Rwanda isolates, Indian (BBTR1,736,523,I.1,BGKVK), Sri.Q553 | Unknown | Mostly Tonga isolates, All AUS,USA & Samoa isolates, Egypt (E.1,E.8,EDDRSA,E.9),T.627,T.V-1a,T.627,Indian isolates(AS-JORB3,I.1,BG;BH2,BT1,Q524), U3-C.Q529_6 | 952-1114/749-885 Around CR-M | 1-29,950-1062 CR-SL | RDP 2.49x10^-12^, GENECONV 6.50x10^-13^, Bootscan 2.98x10^-11^, Maxchi 3.60x10^-08^, Chimaera 6.65x10^-9^, SiScan 3.47x10^-13^, 3Seq 2.20x10^-18^ |
| DNA U3 (5) | Mostly AUS isolates and U3-I.AS-JOR-B3 | All PAK, Rwanda, Malawi & Congo isolates (except Co.Mbk-24), Indian (Q524_2,523,736,BBTR1,I.1), Sri.Q553, | U3-To.TOS93 | 716-919/636-786 Around CR-M | 780-930 CR-M | RDP 8.28x10^-3^, GENECONV 1.70x10^-3^, Bootscan 6.17x10^-4^, Maxchi 5.33x10^-6^, Chimaera 1.55x10^-6^, SiScan 6.03x10^-10^, 3Seq 6.10x10^-9^ |
| DNA U3 (6) | To.TOS71,To.TO114,To.TOS28,To.TOS4,To.TOS56,To.TOS58,To.TOS61,To.TO66,To.TOS74 | Unknown | Mostly Tonga isolates,E.8,E.EDRSA-1991,S.Q279,S.Q281,A.1900B | 900-1270/774-949 Around CR-M | 1-48,831-1062 CR-SL | RDP 0.01635, MaxChi 3.06x10^-3^, Chimaera 3.15x10^-2^, Sisscan 4.23x10^-6^ |
| DNA U3 (7) | I.UM | Unknown | I.MEG | 95-181/94-175 Coding region | 237-318 Coding region | RDP 6.61x10^-5^, GENECONV 5.52x10^-4^,Bootscan 3.38x10^-5^, 3Seq 1.42x10^-2^ |
| DNA U3 (8) | Co.Mvz-80 | Unknown | Co.BU7,Co.BU10,Co.BU13 | 107-1420/94-1014 Coding region & around CR-SL | 98-260 Coding region | RDP 1.13x10^-2^, GENECONV 1.13x10^-3^,Sisscan 1.73x10^-3^ |
| DNA U3 (9) | I.BT1 | I.BG | Unknown | 27-724/26-645 Coding region & around CR-M | 169-788 Coding region & Around CR-M | Sisscan 7.64x10^-7^, 3Seq 1.35x10^-3^ |
| DNA U3 (10) | A.B2845,A.2557,To.TO224,T.V-1a | E.8 | I.1 | 497-911/449-783 Around CR-M | 591-940 CR-M | Siscan 1.89x10^-6^, 3Seq 7.41x10^-5^ |
| DNA S (11) | Mostly Australians isolates, I.Tri8,E.1,R.RAW138,B.BUR,Co.BU13,Co.BU17,U.KP9,Co.BU2,Co.BU6,Co.BU7,Co.BU16,Sri.Q553,I.523_6ACo.Mbk-24,Sri.Kandy,Cam.TV4.1,I.AS-JOR-B5 | Tonga isolates (TO114,TOS22,TOS93,TOS34,TO208,TOS90,TOS65,TOS43,TOS63a&B,TOS64,TOS59,TOS55,TOS70,TOS83,Q278) | I.TN4 | 842-1084/812-1019 Around CR-SL | 147-156,1023-1075, CR-SL | RDP 1.26x10^-2^, GENECONV 3.52x10^-14^ ,Siscan 9.70x10 ^-6^,  Chimaera 3.79x10 ^-2^ |
| DNA S (12) | I.TN4 | Unknown | Co.BU9,I.Trp-DH6 | 867-1084/836-1018 Around CR-SL | 1048-1060,147-156 CR-SL | SiScan 5.91x10^-5^, 3Seq 0.043 |
| DNA M (13) | E.1,A.KP17,A.KP18,A.602,A.1900A,A.1900B | Co.Lubum-9,I.Palani hills,Co.Bmul-77,B.547,Co.BU20,I.BH2,Co.Kwg-31,To.TOS91,To.TOS88,I.BS-JOR-B8,Co.Mvz-80,I.TN4 | Unknown | 89-588/89-567 CR-M | 370-848 coding region & CR-M | RDP 6.65x10^-3^, Bootscan 2.89x10^-2^, Maxchi 8-09x10^-6^,  Chimaera 1.75x10^-8^, SiScan 1.71x10^-4^ |
| DNA M (14) | All Australian isolates except (KP17,KP18,602,1900A,1900B,1429A,1429B,KP7,KP8) All Tonga isolates except (TOS39,TOS40,TOS49,TOS14,TO310,TO314,TO224,TOS91,TOS90,TO208,TOS48) I.736_4 | Unknown | A.1429A,Co.Lubum-9 | 89-518/89-499 Coding region & CR-M | 370-781 Coding region & CR-M | RDP 4.06x10^-4^, GENECONV 6.56x10^-3^,Bootscan 3.17x10^-4^, Maxchi 2.05x10^-5^, Chimaera 2.04x10^-5^, SiScan 2.98x10^-8^,3Seq 9.99x10^-4^ |
| DNA M (15) | Co.Mvz-80,I.Palani hills,Co.Bmul-77,Co.Mbk-24,Co.BU13,Co.BU9,Co.BU19,U.527,B.547,Co.BU20,A.KP8,A.1429A,I.BH2,Co.Kwg-31,P.TH,To224,TOS39,TOS40,TOS49,TOS88,TOS48,TO208,Sri.KP5,I.Tri8,Co.BU14 & 15,I.BT1,I.LK,I.AS-JOR-B8,E..KAL,I.UM,I.1,Co.Lubum-9 | Unknown | E.9,P.TH,To.TOS49,TO208,I.UM,A.B2828,To.KP4,To.TOS60,To.TOS92,To.TOS55,TO.TOS80,To.TOS70,To.536,To.TOS63A,To.TOS71,To.TOS72 | 739-971/713-895 CR-SL | 994- 1046,1-5 CR-SL | RDP 8.35x10^-4^, Bootscan 7.19x10^-4^, 3Seq 1.74x10^-2^,SiScan 5.38x10^-22^ |
| DNA M (16) | Co.Lubum-9 | Co.Kwg-31,Co.Bmul-77,Ma.MAL73 | Unknown^,^ | 257-706/256-678 CR-M | 537-961 End of coding region & CR-M | 3Seq 2.17 x10^-4^,Siscan 1.22 x10^-3^, Maxchi 1.45x10^-2^ |
| DNA C (17) | C-I.LK | All Pak. Isolates, E.1, E.9, A.AUS,I.TN4,I.Tri8, I.AS-JOR-B9 | C-I.UM | 497-1029/489-1012 around CR-M & CR-SL | 728-1018,1-233, CR-M & CR-SL | Siscan 1.16×10^-6^,Chimaera 7.46×10^-7^,MaxChi 1.0510^-5^ |
| DNA C (18) | C-I.1 | E.1,E.9,Pak. Isolates (TJ4,TJ1,NARC,Sakrand,TJ2,TJ3,TH) USA(KP9,527)some Indian, AUS Rwanda, Congo,Tonga and Burundi isolates | C-I.UM,C.I.LK | 345-982/343-965 Around Coging region & CR-SL | 581-1018,1-186, End of coding region & CR-M,CR-SL | MaxChi 2.02 x10^-3^,3Seq 3.08 x10^-7^,Siscan 4.03 x10^-11^ |
| DNA C (19) | C-I.AS-JOR-B9 | To.TO314,To.TO114,To.TOS64,To.TOS57,To.Q277,To.TOS65,To.TOS87,Sri.KP5,Sri.Kandy,Sri.Q553, A.B2830, P.Sakrand | C-Co.Kwg-31,C-Co.Mvz-81 | 524-870/517-854 around CR-M & CR-SL | 754-1018,1-74 CR-M & CR-SL | Siscan 4.97 x10^-5^,MaxChi 2.32 x10^-2^ |
| DNA C (20) | C-I.LK | Unknown | I-AS-JOR-B9,I.Q524_3,A.B2830,A.1429A,To.TOS91,To.TOS64,To.TOS72,To.TO208,Co.BU20,Co.Kwg-31, Sri.KP5 | 343-438/342-431 Coding region | 581-670 Coding region | Siscan 8.81 x10^-5^,3Seq 2.59 x10^-2^ |
| DNA N (21) | Tonga isolates (TOS-39,40,48,90,49,58,TO-310,314,290,224,114,124) | Congo isolates (BU6,13,20,9,2,10,Kwg-31,Mvz-80)I.BGKVK,I.Q524_3,I.BH2,I.1,A.482_98,A.B2819,P.TH,Sr.KP5,Sri.Q553 | Mostly Tonga isolates | 464-739/451-716 CR-M | 727-992 End of coding region & CR-M | 3Seq 5.97 x10^-9^,Siscan 2.37 x10^-7^,Chimaera 3.15 x10^-5^,MaxChi 1.84 x10^-5^,Bootscan 2.72 x10^-5^,GENECONV 7.02 x10^-5^,RDP 5.25 x10^-6^ |
| DNA N (22) | N-To.TOS53 | Unknown | All USA & Samoa isolates, Al Tonga isolates except TOS53, Co.BU7,12,11,E.1, AUS isolates (B2846,25,21,47,34)I.Q524_3,I.BT1,Sri.KP5,T.625I | 6-39/6-39 Coding region | 282-315 Coding region | 3Seq 1.12 x10^-5^,GENECONV 1.98 x10^-8^ |
| DNA N (23) | N-E.1, N-I.BT1 | Unknown | All Sri Lankan isolates, Congo isolates (BU6,13,20,11,9,2,10,Mvz-80,Kwg-31)I.BGKVK,P.TH,I.1,A.B2819,A.482_98 | 352-613/343-589 Coding region & CR-M | 619-870 End of coding region & around CR-M | 3Seq 1.54 x10^-4^,Siscan 1.09 x10^-6^,Chimaera 2.11 x10^-3^,MaxChi 2.86 x10^-3^,Bootscan 1.05 x10^-3^,GENECONV 4.68 x10^-4^,RDP2.85x10^-3^ |
| DNA N (24) | N-Co.BU6 | unknown | All Sri Lanka isolates,E.1,I.BGKVK,I.Q524_3,I.BH2,I.1, Congo isolates (Mvz-80,Mbk-24,BU10,Kwg-31,BU2,BU9,BU11,BU20) | 801-897/773-867 Around CR-SL | 1057-1088,1-57, CR-SL | 3Seq 3.12 x10^-4^,Chimaera 3.06 x10^-2^,MaxChi 3.43 x10^-2^,Bootscan 1.51 x10^-3^,GENECONV 1.02 x10^-4^,RDP 1.76 x10^-5^ |
| DNA N (25) | N-I.UM | N-P.TH | Unknown | 16-404/16-397 Coding region | 292-667 Coding region | Siscan 2.15 x10^-5^,MaxChi 4.65 x10^-2^,Bootscan 5.14 x10^-3^,GENECONV 3.91 x10^-2^ |
| DNA N (26) | All Tonga isolates except (TOS39,TOS48,TOS49,TOS58,TO310,TO224,TO314,TOS90,TOS40),U.KP9,U.527,S.Q281,S.Q279,T.625I | Unknown | Congo (BU1,BU7,BU10,BU13,BU16,BU17,BU2,BU9,Kwg-31,Bmul-77,Mvz-80) Australian (482_96,482_97,482,_98,1429A,1900A,B2834,KP6,KP8,KP17,KP18,A.602) B.526,B.549,I.Q524_3,I.Q524_1,R.RW138,R.RW142,E.8,I.AS-JOR-B12, P.TJ1,P.TJ3,P.TJ4,P.Sakrand,I.Tri8,I.736_4,Ma.MAL73 | 614-926/596-896 Around CR-M & CR-SL | 871-1088,1-85,CR-M & CR-SL | MaxChi 1.52 x10^-3^,Sisscan 2.89 x10^-22^ |
| **Component wise Intergenomic recombination among Asian subgroup** | | | | | | |
| DNA R (27) | T.TW3,T.625I | Unknown | In.IG33,Ph.MS18 | 904-1085/889-1043 CR-SL | 995-1110,1-51CR-M & CR-SL | RDP 4.18x10^-4^, GENECONV 2.67x10^-3^,3Seq 6.38x10 ^-05^10^-^Chimaera 1.10x10 ^-04^,MaxChi 1.73x10 ^-05^ |
| DNA U3 (28) | U3-T.TW3b | Philippine isolates (MS6,MS7,MS15,MS16,MS17,MS18,522A,522B,571_1) Chinese isolates(XTD,HS-5,XP-1,LDH,DW4,HKU,HKU4, Taiwan(Q1160,625,765,Q624,SPb)Indonesian (Q568_1,Q56,520) | Unknown | 120-968/118-839 Around coding region & CR-M | 262-967 coding region & CR-M | 3Seq1.49 x10^-38^,Siscan 7.54 x10^-23^,MaxChi 1..81 x10^-14^,Bootscan 8.74 x10^-8^,GENECONV 9.94 x10^-13^,RDP 4.54 x10^-12^,c |
| DNA U3 (29) | U3-T.Spa, U3-Ph.MS14 | All Philippine isolates except (Ph.MS14 & Ph.571_2),T.SPb,T.Q623,T.Q624,T.Q1160,In.Q568 | Unknown | 24-911/23-786 Around CR-M & Coding region | 165-928 Coding region & CR-M | RDP 4x10^-2^, GENECONV 2.25x10^-03^, Siscan 7.58x10^-09^, Maxchi 1.22x10^-5^, Chimaera 7.25x10^-73^, 3Seq 8.69x10^-11^ |
| DNA U3 (30) | C.HKU4 | C-HAIN,C.HKU2,C.CMH, C.DZHD,C.HF-1,C.HKU3 | T.MP1, T.MP2, | 633-691/576-625 around CR-M | 721-756 CR-M | MaxChi 1.04x10^-3^, 3Seq 7.66x10^-4^ |
| DNA U3 (31) | T.TW3a,T.TH16,T.TH16,C.HF-1,C.DZH,C.XTD,C.HS-5,C.LDH,C.DW4,C.HKU,T.SPb,T.Q623,T.Q624,In.520,T.Q1160,In.Q568,Ph.522B,Ph.571,Ph.571_1,T.Spa,T.MP1,T.MP2 | Unknown | C.Q529_4,C.Q529_2 | 202-558/193-505 Around coding region | 338-651 End of coding region | MaxChi 1.28×10^-3^,Sisscan 4.79×10^-9^ |
| DNA-S (32) | S-T.MP2,T.626M,T.626 | Unknown | All isolates of Japan, Indonesia and Philippine,C.HKU,C.HAIN;C.HKU, Taiwan isolates(V-1b,625,627,TAI,765,Q1160,Q623,Q624,SP),I.523_6B,I.523_6A,E.8 | 864-986/817-931 Around CR-SL | 1044-1058,1-65 CR-SL | RDP 6.23x10^-10^, GENECONV 1.04x10^-9^,Bootscan 4.91x10^-10^, Maxchi 1.35x10^-3^, Chimaera 1.27x10^-3,^3Seq 3.40x10^-07^ |
| DNA-M (33) | C.HAIN,C.HF-1,Ph.522A | C.DW4 | Unknown | 506-617/501-593 CR-M | 768-876 CR-M | Maxchi 1.94x10^-4^, Sisscan 4.52x10^-14^ |
| DNA M (34) | T.SP,T.Q623,T.Q624,T.Q1160,T.MP2,E.8,T.765 | Unknown | All Philippine isolates, C.HKU,C.HS-5,C.XTD,T.627,T.V-1a,I.523,I.523_6a,In.520,In.Q568,In.Q568_1 | 733-886/701-810 CR-SL | 988-1046, 1-51 CR-SL | Bootscan 1.56x10^-3^, GENECONV 4.71x10^-2^, Siscan 3.32x10^-12^, Maxchi 4.46x10^-02^, Chimaera 4.93x10^-2^, 3Seq 1.57x0^-6^ |
| DNA C (35) | C-T.TH16,C-C.Q529,C-C.Q529_2 | Unknown | C-C.HAIN | 259-899/259-879 coding region & CR-SL | 497-1018,1-103, End of coding region CR-M & CR-SL | Siscan 2.66 x10^-13^,Chimaera 5.33 x10^-5^,MaxChi 1.28 x10^-4^ |
| DNA C (36) | C.XP-1 | Unknown | Taiwan isolates (SP,Q623,Q624,MP2) Philippine isolates (,571_1,MS17,MS7) In.Q568_3, I.523_6B | 49-89/49-89 Coding region | 287-328 Coding region | RDP 2.86 x10^-3^,GENECONV 1.43 x10^-2^ |
| DNA N (37) | C.NS,C.HS-5,XP-1,C.XTD | T.SP,I.523_6B,T.Q623,S.Q280,In.520,Ph.768,Ph.522,Ph.MS7,Ph.571_1,Ph.571_2,T.625 | Unknown | 584-1037/568-994 Around CR-M & CR-SL | 843-1088,1-190, CR-M & CR-SL | Chimaera 1.49 x10^-2^,Sisscan 5.57 x10^-14^ |
| **Component wise intergenomic recombination between South Pacific and Asian subgroup isolates** | | | | | | |
| DNA-R (38) | To.TOS5,Co.Mbk-23,I.AS-JOR-B2,Co.BU16,M.MY01,A.B2847,To.Q277,To.TOS57,I.Trp-N6,I.Trp-N5,I.UM,C.HAIN,C.HKU4,In.BSI,T.MP2 | V.BN | I.1 | 842-997/836-985 Around CR-M | 937-1082 End of Coding region & CR-M | Siscan 7.02x10 ^-7^,3Seq 0.037404 |
| DNA R (39) | V.BMT,V.DN,I.Trp-SO6,I.LK,Co.BU15,F.FIJ,I.TN4,Sri.Kandy,To.TOS86,To.TO310,To.TOS39,To.TOS65,To.TOS91,T.V-1a,I.Trp-KH1,C.Q529_6,V.DN | Ph.MS16 | T.625I | 915-1077/900-1051 Around CR-M & CR-SL | 1005-1110, 1-43 CR-M & CR-SL | RDP 1.10x10^-2^, GENECONV 4.63x10^-3^,3Seq 2.60x10 ^-7^,MaxChi 4.80x10 ^-3^ |
| DNA R (40) | S.Q279,S.Q281,P.TJ2,I.Manp9-gn,I.Trp-SH3,SH8,I.Trp-SO5,I.DL,B.548,Co.BU16,Sri.KP5,A.482P2,Co.Kiy-106,To.Q277,To.TON,To.TOS57,To.TOS63A,To.TOS93,To.TOS5,To.TOS72 | C.HKU2 | I.TN3 | 79-170/77-167 Coding region | 177-268 Coding region | RDP 1.29x10^-5^, GENECONV 1.01x10^-5^,3Seq 1.76x10 ^-7^ |
| DNA R (41) | I.TN3 | I.523_6A | V.HCM | 236-638/234-633 Coding region | 334-733 Coding region | MaxChi 1.51x10^-5^,Chimaera 9.33x10^-3^ |
| DNA U3 (42) | Chinese isolates (HF-1,HKU1-3,CMH,HAIN,DZH;DZHD) Thailand.(TH16.1,TH16.2,TH16.3) | Mostly Tonga isolates, All AUS,USA & Samoa isolates, Egypt (E.1,E.8,EDDRSA,E.9),T.627,T.V-1a,Sri.Kandy,Indian isolates(AS-JORB3,I.1,BG;BH2,BT1,Q524), U3-C.Q529_6 | Unknown | 954-1105/821-953 Around CR-M | 957-1042 Around CR-SL | RDP 1.05x10^-14^, GENECONV 3.52x10^-14^, Bootscan 1.78x10^-12^, , 3Seq 1.68x10 ^-20^,Siscan 2.19x10 ^-18^,Chimaera 6.94x10 ^-08^,MaxChi 4.99x10 ^-08^ |
| DNA U3 (43) | C.Q529_4,C.Q529_2 | Australain isolates (AUS,B2845,482_96,482_97,KP6,14,15,16,17,B2825,B2817,B2818,B2822,B2823,B2830,B2824,B2821,B2819,B2827,B2829,2557,1900B,602),Tonga (208,224,290,TOS29,78,55,20,60,63A)T.627,T.V-1a,I.UM,I.LK,I.MEG,I.BH2,E.1 | C.HKU,C.XTD,C.HS-5,C.LDH,C.DW4,C.HKU4,T.SPb,T.Q623,T.Q624,T.765,Ph.MS6,Ph.MS15,Ph.MS18,In.520,Ph.MS7,Ph.MS16,Ph.MS17,T.Q1160,Ph.522A,Ph.522B,In.Q568,In.Q568_1,T.625,Ph.571_1 | 949-1052/817-911 Around CR-M | 874-953 1-29,CR-SL | GENECONV 1.65x10^-9^, Bootscan 1.75x10^-3^, Siscan 7.88x10 ^-6^,Chimaera 5.03x10 ^-4^,MaxChi 7.86x10 ^-6^ |
| DNA U3 (44) | All Asian isolates except Ph.MS14,C.Q529_4,C.Q529_2 | I.MEG,I.UM | Unknown | 708-944/625-796 Around CR-M | 772-947 CR-M | RDP 2.72x10^-2^,Bootscan 9.12x10^-3^, 3Seq 2.23x10 ^-6^,Siscan 1.43x10 ^-21^,Chimaera 1.34x10 ^-4^,MaxChi 2.03x10 ^-6^ |
| DNA U3 (45) | All Asian isolates except Ph.MS14,Th.TH16,C.Q529_4,C.Q529_2 | Mostly Tonga & all Australian isolates,E.1,E.8,E.9,E.EDRSA-1991,S.Q279,S.Q281,U.KP9,U.527,T.627,T.V-1a,Co.BU2,Co.BU11,Co.BU12,I.TN4,I.Q524_2,I.AS-JOR-B3 | Unknown | 25-201/24-191 Coding region | 166-337 Coding region | RDP 3.05x10^-5^, Bootscan 8.16x10^-5^, 3Seq 5.20x10 ^-7^,Chimaera 1.27x10 ^-4^,MaxChi 3.29x10 ^-4^ |
| DNA U3 (46) | To.TOS93 | S.Q281 | T.Spa | 708-842/628-733 CR-M | 772-873 CR-M | MaxChi 2.34x10^-6^, 3Seq 1.37x10^-2^ |
| DNA U3 (47) | C.Q529_4,C.Q529_2 | Unknown | Congo isolates (BU10,BU20,BU16,BU19,BU18,BU15,BU12,BU17,BU7,Kwg-31,BU13,)A.B2847,A.B2817,To-TOS20.To.TOS78,To.TO208 | 26-105/25-104 Coding region | 167-247 Coding region | GENECONV 1.23x10^-4^, Sisscan3.72x10^-23^ |
| DNA U3 (48) | To.TOS43,To.TOS69,To.TOS59,C.LDH,C.DW4,C.HKU4,C.HKU,T.SPb,T.Q623,In.520,In.Q568,h.522B,Ph.571,Ph571_1,T.MP1,T.MP2 | Ph.MS14 | To.TOS87 | 37-69/36-68 Coding region | 178-209 Coding region | GENECONV 1.68x10^-3^,Maxchi 1.62x10^-2^, SiScan 8.21x10^-9^ |
| DNA U3 (49) | U3-T.TW3b | Mostly Congo, AUS & Tonga isolates, E.1,E.8,,B.526,T.TH16,B.526,B.547,I.523,I.Q524_2,Sri.Q553 | Unknown | 38-66/38-66 Coding region | 179-206 Coding region | RDP 1.26x10^-4 ,^GENECONV 1.89x10^-2^, 3Seq 1.40x10^-2^ |
| DNA S (50) | All Burundi, USA,Sri Lanka,Samoa,Rwanda,Fiji,Malwai,Tonga,Australian isolates except (A.B2819,A.B2830) All Pakistani isolates except (KM,NR)Some Indian & all Congo isolates, Cam.TV4.1,All Asian isolates except C.Q529_1,3,4,T.TW3,V.V6,T.625I,T.627 | V.V14 | A.B2819 | 482-829/479-799 CR-M | 691-1011 End of coding region & CR-M | RDP 0.0385, GENECONV 3.52x10^-14^, 3Seq 4.0x10 ^-2^,  Siscan 3.61x10 ^-22^,Chimaera 2.08 x10 ^-05^,MaxChi 4.24x10 ^-6^ |
| DNA M (51) | All Asian isolates | Co.Lubum-9 | Unknown | 280-972/279-896 CR-SL | 560-1046,1-130 end of coding region CR-M & CR-SL | RDP 6.28x10^-6^, GENECONV 3.49x10^-5^,Bootscan 1.47x10^-3^, Maxchi 4.08x10^-8^,Chimaera 2.06x10^-5^, SiScan 4.16x10^-23^,3Seq 2.28x10^-3^ |
| DNA M (52) | T.TW3 | E.9,P.NARC,Co.BU1,R.RW142,R.RW138,Co.BU6,Co.BU7,Co.BU12,Co.BU17,B.521,Sri.Q553,To.TO310,To.TO314,To.TOS85,I.SL | All Philippine isolates except Ph.MS6,T.627,All Indonesian isolates, I.523,I.523_6A | 793-935/740-848 CR-SL | 1028-1046,1-93 CR-SL | RDP 5.06x10^-5^, SisScan 3.41x10^-20^ |
| DNA-C (53) | All Philippine and Chinese isolates except (Hain,Q529,Q529_2)T.Q1160,T.627,I.523_6B,In.520,In.Q568_3,In.Q568_1 | All Pakistan, USA; Samoa, Malawi and Sri Lankan isolates, Few Congo, Tonga and Australian isolates,Indian isolates (UM,AS-JOR-B9,Q524 1-3,TN4,BH2,I:1,I.736) E.1,T.V-1a,B.526,B.547,B.548 | T.MP2,T.Q623 | 763-839/745-819 Around CR-SL | 987-1018,1-44, CR-SL | RDP 2.13x10^-7^, GENECONV 4.77x10^-8^, 3Seq 2.34x10 ^-4^,Siscan 2.19x10 ^-18^ |
| DNA C (54) | C-T.TH16,C-C.Q529,C-C.Q529_2 | Tonga isolates (TO314,TOS39,TOS40,TOS48,Q570.TO310,TO208,TO224,TO290,TOS90,Q276,536,TOS42,TOS43,TOS78,TOS4,TOS64,TOS57,TOS70,TOS59,TOS65),Sri.KP5,Sri.Kandy,T.V-1a, Australian isolates (B2845,B2844,B2848,B2846,A.2557,1429B,KP7,KP8,B2830),U.KP9,U.527,Co.BU9,Co.BU12,Ma.MAL73,Co.BU1,Co.BU10,B.526 | Unknown | 774-849/754-828 Around CR-SL | 997-1018,1-53, CR-SL | 3Seq 2.29×10^-2^,GENECONV 7.67×10^-5^ |
| Note: ^1^The recombinants, major and minor parent isolates are denoted using abbreviation of isolates in (Table 1)  ^2^The intergenomic recombination among and between subgroups of BBTV was determined based on full-length sequences which were started from the first nucleotide of major ORF present in each component and were aligned using MAFFT version 6.864 (Katoh et al., 2002).  ^3^Various methods including GENECONV (Padidam et al., 1999), Bootscan (Salminen et al., 1995),Maxchi (Maynard, 1992), Chimaera (Posada and Crandall, 2001), SiSscan (Gibbs et al., 2000), LARD (Holmes et al., 1999) and 3Seq (Boni et al., 2007) implemented in Recombination Detection Program (RDP) version 4 Beta14 (Martin et al., 2005) were used for detection of recombination events given in parenthesis against each component. | | | | | | |
