# Supplementary material for: Banana bunchy top virus genetic diversity in Pakistan and association of diversity with recombination in its genomes
Source: PLoS One. 2022 Mar 7;17(3):e0263875. doi: 10.1371/journal.pone.0263875 (PMC8901069; doi:10.1371/journal.pone.0263875)
Supplement: S3 Table — (DOCX) [file pone.0263875.s003.docx]

| **S3 Table.** **Intragenomic recombination in Banana bunchy top virus genomes.** | | | | | | |
| --- | --- | --- | --- | --- | --- | --- |
| **Event** | **Recombinant** | **Minor Parent** | **Major Parent** | **Position^1^ in alignment/in recombinant** | **Position of recombinant according to TJ1** | **Methods^2^ with average probability values** |
| **Intragenomic recombination between South Pacific and Asian subgroups** | | | | | | |
| Event#55 | All DNA-S isolates | Unknown | DNA-S-A.B2819 | 1008-1604/605-908 Some nucleotide Of CR-M and major portion of CR-SL | 817-1075,1-44 CR-M & CR-SL | RDP 8.42x10^-32^, GENECONV 1.33x10^-30^, Bootscan 1.03x10^-27^, Maxchi 5.65x10^-11^, Chimaera 4.27x10^-11^, SiSscan 3.53x10^-16^, 3Seq 3.39x10^-09^ |
| Event#56 | DNA-M all Asian isolates | All DNA-U3 Asian isolates except C.XTD,C.HS-5, C.LDH, C.XP-1,C.DW4,C.HKU4,C.HKU, T.MP1, Ph.571_1 | All DNA-M South Pacific isolates | 959-1110/488-572 CR-M | 829-847 CR-M | RDP 2.29x10^-13^, GENECONV 6.14x10^-16^, Bootscan 2.05x10^-14^, SiSscan 2.19x10^-02^, 3Seq 1.70x10^-08^ |
| Event#57 | DNA-R-T.MP2 | Some DNA-M Asian isolates (T.MP2,T.765,T.Q1160,E.8,T.Q623,624,T.SP) | All DNA-R isolates | 1380-1642/972-1049 CR-SL | 1078--1110,1-44, CR-SL | RDP 1.90x10^-14^, GENECONV 1.55x10^-14^, SiSscan 2.31x10^-4^, 3Seq 3.76x10^-07^ |
| Event#58 | DNA C (T.765,T.SP,T.Q623,T.Q624,T.625,T.MP2,Co.MVZ-81) | DNA M (T.SP,T.765,T.Q1160,T.Q623,T.Q624,T.MP2,E.8) | Some DNA C South Pacific and Asian isolates | 1384-1652/748-845 CR-SL | 1-67,989-1018 CR-SL | RDP 1.14x10^-9^, GENECONV 2.45x10^-11^, Bootscan 1.38x10^-2^, SiSscan 3.42x10^-15^, 3Seq 9.49x10^-4^ |
| Event#59 | All DNA R Asian isolates except T.TW3 and T.625I | Unknown | All DNA R South pacific isolates except To.TOS63A and To.TOS42 | 999-1213/893-963 CR-M | 998-1070 CR-M | RDP 8.42x10^-11^, GENECONV 2.11x10^-8^,Maxchi 6.69x10^-3^,, 3Seq 8.87x10^-06^ |
| Event#60 | Some DNA U3 Tonga isolates (TOS69,TOS28,TOS4,TOS58,TOS56,TOS61,TOS62,TOS71,TOS74,TOS83,TOS59,TO114,TO166) | DNA U3 some Chinese (HKU1,HKU2,HKU3,CMH,HAIN;DZH;DZHD,HF-1) and Thailand isolates | DNA U3 all Australian,Samoa & USA isolates, Egypt (E.1,E.8,E.9;E.EDRSA) Taiwan (T.627,T.V-1a) Indian (TN4,Q524_3,Q524_1,Q524_2,BH2,BT1,I.1;BG,AS-JOR-B3) Sri.Kandy & some Tonga isolates | 1292-1472/808-899 Around CR-M | 1-19,953-1062, CR-SL | RDP 1.82x10^-13^, GENECONV 8.32x10^-12^, Bootscan 1.14x10^-10^, Maxchi 2.41x10^-4^, Chimaera 3.31x10^-5^, SiSscan 7.30x10^-4^, 3Seq 1.40x10^-15^ |
| Event#61 | All DNA U3 Asian isolates except Ph.571_2 | Unknown | DNA U3 all Tonga, Congo,Pakistan, Burundi, USA, Malawi, & Australian (except four) isolates, Indian (BH2,KRL1,736,523,BGKVK;TN4Q524_3,Q524_1,Q524_2,BRJT9,BRT1,BG,I.1,AS-JOR-B3,LK) S.Q279,E.9;T.627,T.V-1a,Sri.Kandy,Sri.Q553,R.RW142,C.Q529_6 | 930-1081/583-659 Around CR-M | 730-813,CR-M | RDP 2.29x10^-12^, GENECONV 6.76x10^-10^, Bootscan 3.13x10^-9^, Maxchi 2.53x10^-2^, Chimaera 7.07x10^-3^,3Seq 2.03x10^-07^ |
| Event#62 | All DNA C south pacific isolates | All DNA U3 Tonga, Pakistan. Sri Lanka, Burundi,USA, Malawi, Australian except (1900A,KP17,KP18,602) Congo except Co.Mbk-24,R.RW142, C.Q529_6,S.Q279, E.9, T.627,T.V-1a, ) Indian isolates (736,BH2,KRL1,523,TN4,BGKVK,Q524 1-3,BRJT9,BG,BT1,I.1,AS-JOR-B3,LK),C.Q529_6,Q529_4,Q529_2, DNA M-P.NARC | All DNA C Asian isolates | 993-1080/601-664 CR-M | 816-905 CR-M | RDP 6.72x10^-10^, GENECONV 1.31x10^-6^, Bootscan 7.21x10^-9^, SiSscan 1.81x10^-35^, 3Seq 1.28x10^-05^ |
| Event#63 | DNA M Asian isolates except (T.TW3,T.765,T.MP2,T.Q623&624,T.Q1160,T.SP,E.8) All DNA M Pakistan,Sri Lanka,USA,Burundi,Samoa,Tonga,Rwanda,Malawi, Australain & mostly Congo isolates, some Indian isolates | Mostly DNA R Asian and South pacific isolates | Unknown | 1386-1644/731-818 CR-SL | 1019-1046,1-59 CR-SL | GENECONV 3.82x10^-3^, Bootscan 2.79x10^-7^, SiSscan 8.52x10^-8^ |
| Event#64 | DNA U3-Ph.571, U3-Ph.MS14 | Mostly DNA N isolates | DNA U3 all Thailand,Egypt,Australian,Samoa,USA isolates,Chinese isolates( HKU1-3,HF-1,CMH,HAIN,DZH,DZHD,Q529_6) Indian isolates (TN4,Q524_1-3,BRJT9,BH2,AS-JOR-B3,LK) Sri.Kandy,T.627,T.V-1a and mostly Tonga isolates | 1383-1530/803-865 Around CR-M | 1-39,1011-1062, CR-SL | RDP 9.75x10^-3^, GENECONV 1.84x10^-8^ |
| Event#65 | DNA U3 Thailand isolates | Unknown | All DNA U3 Tonga,Pakistan, Burundi,Malawi,Sri Lanka, Congo except Mbk-24, Australian except (1900A,KP17,KP18,602) Indian isolates (736,BH2,KRL1,523,TN4,BGKVK,Q524,BRJT9,BG,BT1,I.1,AS-JOR-B3,LK) E.9,T.627,T.V-1a, C.Q529_6 | 990-1075/595-653 Around CR-M | 744-805, CR-M | RDP 4.38x10^-9^, GENECONV 8.76x10^-10^, Bootscan 5.63x10^-7^, 3Seq 4.39x10^-4^ |
| Event#66 | DNA U3-C.Q529_4,C.Q529_2 | Unknown | DNA U3 isolates (To.TOS12,To.TOS29,To.TOS78,To.TOS87,To.TOS28,To.TOS4,To.TOS56,To.TOS71,To.TO114,I.AS-JOR-B3) | 958-1058/596-649 Around CR-M | 736-790 CR-M | GENECONV 8.30x10^-03^, SiSscan 1.04x10^-28^ |
| Event#67 | DNA C (T.TW3,Co.550,Co.Lubum-9,Co.Bmul-77,A.1429A,A.482 96-98,B.549) some Tonga isolates (TO121,TOS87,TO124,TO166,TOS16,TOS56,TOS61,TOS62,TOS22,TOS12,TOS21,TOS28,TOS29,TOS25,TOS76,TOS20,TOS2,TOS93,TOS91,TOS58,TOS82) | DNA U3 (T.TW3a,T.625I) | DNA C (C.Q529_4,C.Q529_2,Th.TH16) | 1358-1570/738-806 Around CR-SL | 980-1018,1-39 CR-SL | RDP 1.07x10^-07^, GENECONV 1.99x10^-06^ |
| **Intragenomic recombination among South Pacific isolates** | | | | | | |
| Event#68 | DNA U3 all Pakistani,Congo, Burundi & Rwanda isolates, some Indian isolates (I.KRL1,I.BBTR1,I.736,I.523,I.BGKVK),Sri.Q553, | Unknown | DNA U3 all Australian,USA & Samoa isolates, Indian (BG,TN4,Q524_3,Q524_1,Q524_2,BRJT9,BH2,BT1,I.1),Egypt (E.1,E.8,E.9,E.EDRSA-1991) Taiwan (T.627,T.V-1a) Sri.Kandy & some Tonga isolates | 1353-1484/854-946 Around CR-M | 999-1062,1-29 ,CR-SL | RDP 2.77x10^-14^, GENECONV 1.67x10^-14^, Bootscan 1.37x10^-7^, Maxchi 1.59x10^-3^, Chimaera 4.26x10^-4^, SiSscan 1.56x10^-5^, 3Seq 3.80x10^-13^ |
| Event#69 | DNA-U3 isolates (R.RW138,Co.Mbk-24,A.KP17,18,A.1900A,A.602,S.Q281,E.1,8,E.EDRSA-1991,I.MEG,I.UM) DNA-N isolates (Co.BU17-19,I.1,A.737,To.TOS80,87,5,57,71,72,63A,Q276-278,S.Q279,T.625I) | All DNA-R South pacific isolates and DNA-M isolates (E.1,E.KAL, some Tonga and Australian isolates) | Some DNA-U3 Tonga isolates (To.TOS42,43,67,68,55,63A,65,72,93) | 1028-1149/632-705 CR-M | DNA-U3 773-783 CR-M  DNA-N 896-912 End of CR-M | RDP 4.29x10^-12^, GENECONV 6.23x10^-15^, Bootscan 3.28x10^-11^, SiSscan 1.99x10^-02^, 3Seq 7.51x10^-04^ |
| Event#70 | DNA N-To.TOS93 | All DNA U3 Pakistan, Malawi,Burundi Tonga isolates except (To.208,To.224,To.290) Australian except (1900A,2557,KP17,KP18) Congo except (Lubum-9,BU13) S.Q279,Sri.Q553,E.9;T.627,T.V-1a, Indian isolates (LK,AS-JOR-B3,I.1,BG,BT1,Q524 1-3,BRJT9,TN4,BGKVK,523,736,KRL1) C.Q529_6 | DNA N (E.1,I.BT1) | 962-1081/531-607 CR-M | 778-882 CR-M | RDP 3.40x10^-10^, GENECONV 6.42x10^-6,^ 3Seq 3.66x10^-04^ |
| Event#71 | DNA M-Co.MVZ-80 | Unknown | All DNA M Pakistan,Sri Lanka Rwanda,Burundi.Malawi,USA,Samoa, Congo except Lubum-9, Australian except (B2829,KP16) Mostly Tonga isolates,E.1,E.9;E.KAL, Indian (UM,SL,AS-JOR-B3,TN4,LK,BT1,Tri8,BH2,I.2,Palani hills,Q524_3,Q524_1) | 1384-1640/736-821 Around CR-SL | 1002-1046,1-55CR-SL | GENECONV 4.40x10^-9^, Bootscan 4.04x10^-6^ |
| Event#72 | DNA R (A.482p2,A.482_96-98,M.MY01,MY03,M.MAL73E.9,B.549) Congo isolates (Tshil-75,Bmul-63,65,70,Kip-15,16,Lubum-9,4,Kase-111,Kbd—66,Nsan,550,Kwg-28,31,37,41,49,57), Tonga isolates (TOS91,TOS65,TOS76,TOS85,TOS2,TOS46,TOS15,TOS77,TOS90,TO121,Q276,Q277,KP4) | DNA C (To.TOS14,To.TOS15) | DNA R-I.1 | 1392-1644/990-1056 CR-SL | 1098-1110,1-44 CR-SL | RDP 5.83x10^-08^, GENECONV 2.10x10^-07^ |
| Event#73 | DNA-U3-Co.MVZ-80 | Unknown | DNA-U3 (Co.BU10,Co.BU11,Co.BU7,Co.BU13,Co.BU2) | 54-173/22-92 Coding region | 165-247 Coding region | RDP 1.52x10^-4^, GENECONV 1.46x10^-4^, Bootscan 5.23x10^-3^, |
| Event#74 | Some DNA U3 south pacific isolates (To.TOS71,TO114,TOS28,TOS4,TOS58,TOS56,TOS62,TOS61,TO166,TOS74,TOS83) | Unknown | DNA U3 (To.TOS60,To.TOS93,I.AS-JOR-B3,E.1,E.8,E.EDRSA-1991) | 1264-1282/790-806 Around CR-M | 933-949 CR-SL | GENECONV 1.21x10^-04^, SiSscan 1.29x10^-49^ |
| Event#75 | DNA U3-I.MEG | Unknown | DNA U3-I.UM | 183-256/112-173 Coding region | 256-318 Coding region | RDP 1.03x10^-03^, GENECONV 1.32x10^-02^ |
| Event#76 | DNA U3 (C.Q529_6,I.Q524_3,I.Q524_1,Sri.Kandy) | DNA U3 (To.TOS80,To.TOS29,To.TOS55,To.TOS82,To.TOS68,To.TOS63B,To.TOS43,To.TOS25,To.TOS21,To.Q278) | DNA U3-I.1 | 1084-1437/669-915 Around CR-M | 812-1062 Around CR-M | Maxchi 7.07x10^-3^, Chimaera 9.41x10^-3^, SiSscan 4.61x10^-3^ |
| Event#77 | DNA M-E.1 | Unknown | DNA M (I.Tri8,I.UM) | 640-1259/253-656 Around coding region &CR-M | 534-936 End of coding region & CR-M | Maxchi 2.84x10^-2^, Chimaera 2.90x10^-2^, SiSscan 8.58x10^-7^ |
| **Intragenomic recombination among Asian isolates** | | | | | | |
| Event#78 | DNA U3-T.TW3b | DNA-S of a Chinese isolate (C.HAIN) from Asian subgroup | DNA-U3 of all Philippine, Indonesian and Taiwan (except tw3a and spa) isolates, Chinese isolates (XTD, HS-5,HP-1, LDH, DW4, HKU) | 34-1388/19-858 Around coding region & CR-M | 162-1016 Coding region & CR-M | RDP 1.99x10^-18^, Maxchi 2.85x10^-12^, , Bootscan 2.06x10^-12^, Maxchi 5.07x10^-10^, Chimaera 5.34x10^-11^, SiSscan 1.08x10^-9^, 3Seq 3.85x10^-24^ |
| Event#79 | DNA-N all Asian isolates | DNA-U3 all Asian isolates | DNA-N (E.1, I.BT1) | 992-1066/542-590 CR-M | 818-869 CR-M | RDP 6.54x10^-15^, GENECONV 3.32x10^-16^, Bootscan 1.39x10^-11^, Maxchi 4.44x10^-03^, 3Seq 6.78x10^-09^ |
| Event#80 | Some DNA-U3 Asian isolates( C.HKU,C.HKU4,C.XTD,C.HS-5,C.XP-1,C.LDH,C.DW4,T.SPb,T.Q624-Q623,T.765,T.Q1160,T.725,T.TE3b,T.MP1&2,Ph.522A&B,Ph.571_1,Ph.MS6,7,15,16,17,18,In.Q568,568_1 | Unknown | All DNA-U3 Thai´s Isolates and Chinese (HF-1, HKU1,HKU2,HKU3,C.CMH,C.HAIN,C.DZH,C.DZHD) | 1290-1484/800-927 Around CR-M | 1-28,951-1062 CR-SL | RDP 2.55x10^-12^, GENECONV 5.54x10^-15^, Bootscan 3.45x10^-10^, Maxchi 8.42x10^-8^, Chimaera 9.11x10^-7^, SiSscan 1.40x10^-10^, 3Seq 1.11x10^-13^ |
| Event#81 | DNA U3-Ph.MS14 | Unknown | Some DNA U3 isolates (C.HKU1,C.HKU2,C.HKU3, C.HAIN,C.DZH,T.TH16) | 1236-1382/777-873 Around CR-M | 918-1010 around CR-SL | RDP 2.41x10^-4^, GENECONV 2.08x10^-2^ , SiSscan 3.33x10^-06^ |
| Event#82 | DNA M (T.MP2,T.765,T.Q1160,E.8,T.Q624,Q623,T.SP,T.TW3) | Unknown | All DNA M Asian isolates except T.TW3,T.765,T.MP2,T.SP,T.Q623,T.Q624,T.Q1160 | 1384-1639/730-814 Around CR-SL | 1017-1046,1-54 CR-SL | GENECONV 9.70x10^-8^, Bootscan 2.38x10^-7^ |
| Note: Please refer to Table 1 for the nomenclature of isolates.  ^1^The analysis for recombination is determined based on full-length sequences which were started from the first nucleotide of major ORF present in each component and  were aligned using MAFFT version 6.864 (Katoh et al., 2002).  ^2^ Various methods including GENECONV (Padidam et al., 1999), Bootscan (Salminen et al., 1995), Maxchi (Maynard, 1992), Chimaera (Posada and Crandall, 2001), SiSscan (Gibbs et al., 2000), LARD (Holmes et al., 1999) and 3Seq (Boni et al., 2007) implemented in Recombination Detection Program (RDP) version 4 Beta14 (Martin et al., 2005) were used for detection of recombination events. | | | | | | |
