# Supplementary figures and images for: Banana bunchy top virus genetic diversity in Pakistan and association of diversity with recombination in its genomes
Source: PLoS One. 2022 Mar 7;17(3):e0263875. doi: 10.1371/journal.pone.0263875 (PMC8901069; doi:10.1371/journal.pone.0263875)

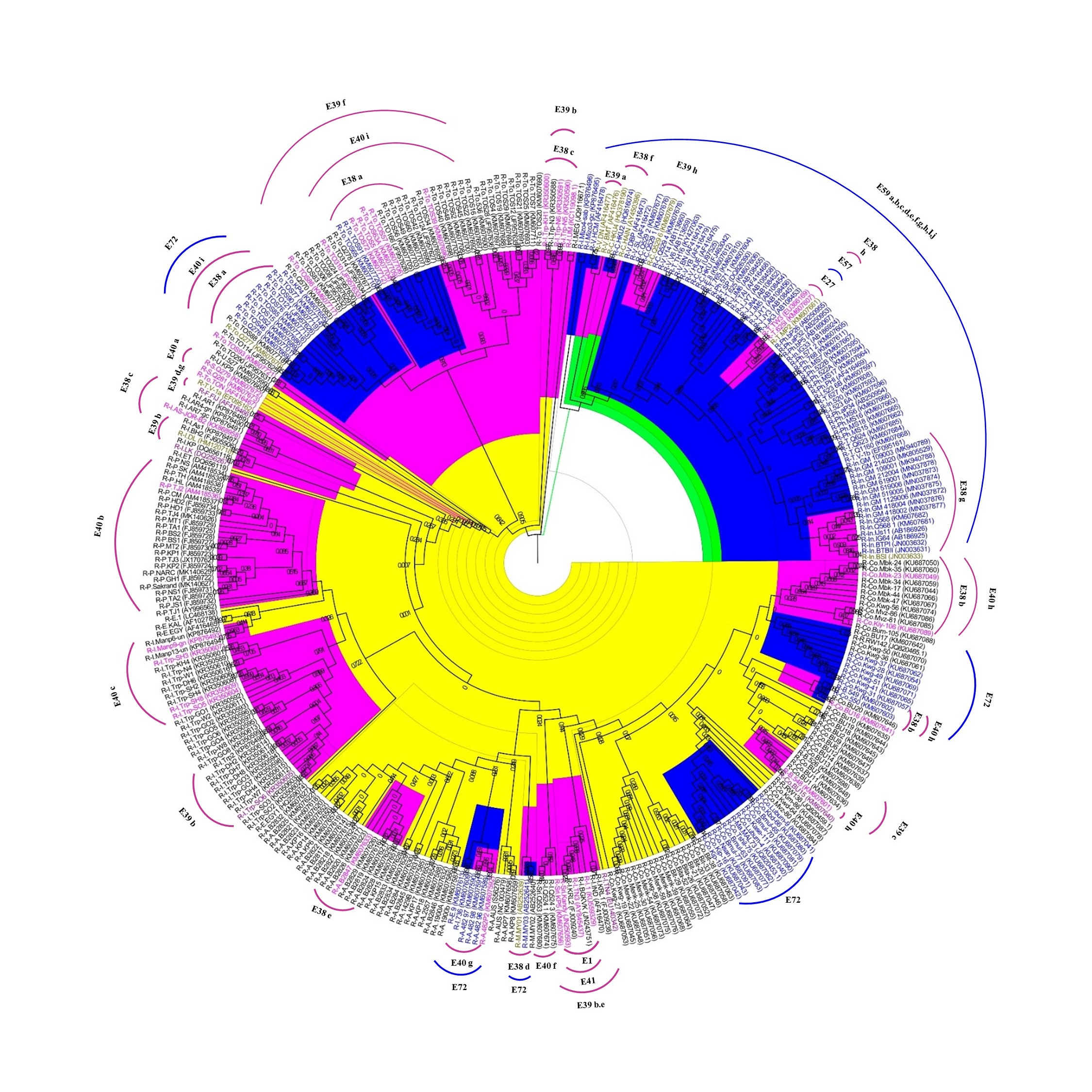

Supplement: S1 Fig — (TIF) [file pone.0263875.s004.tif]

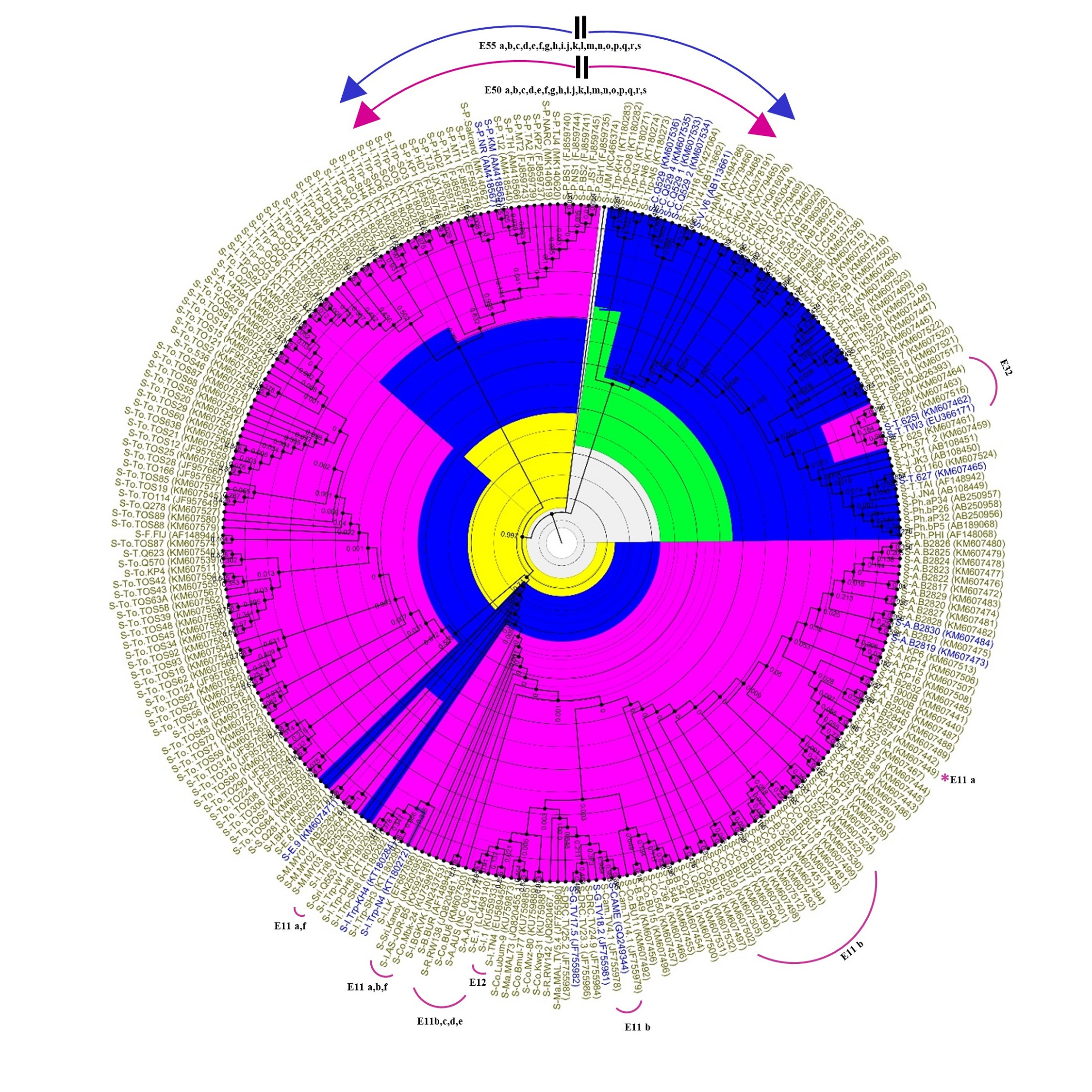

Supplement: S2 Fig — (TIF) [file pone.0263875.s005.tif]

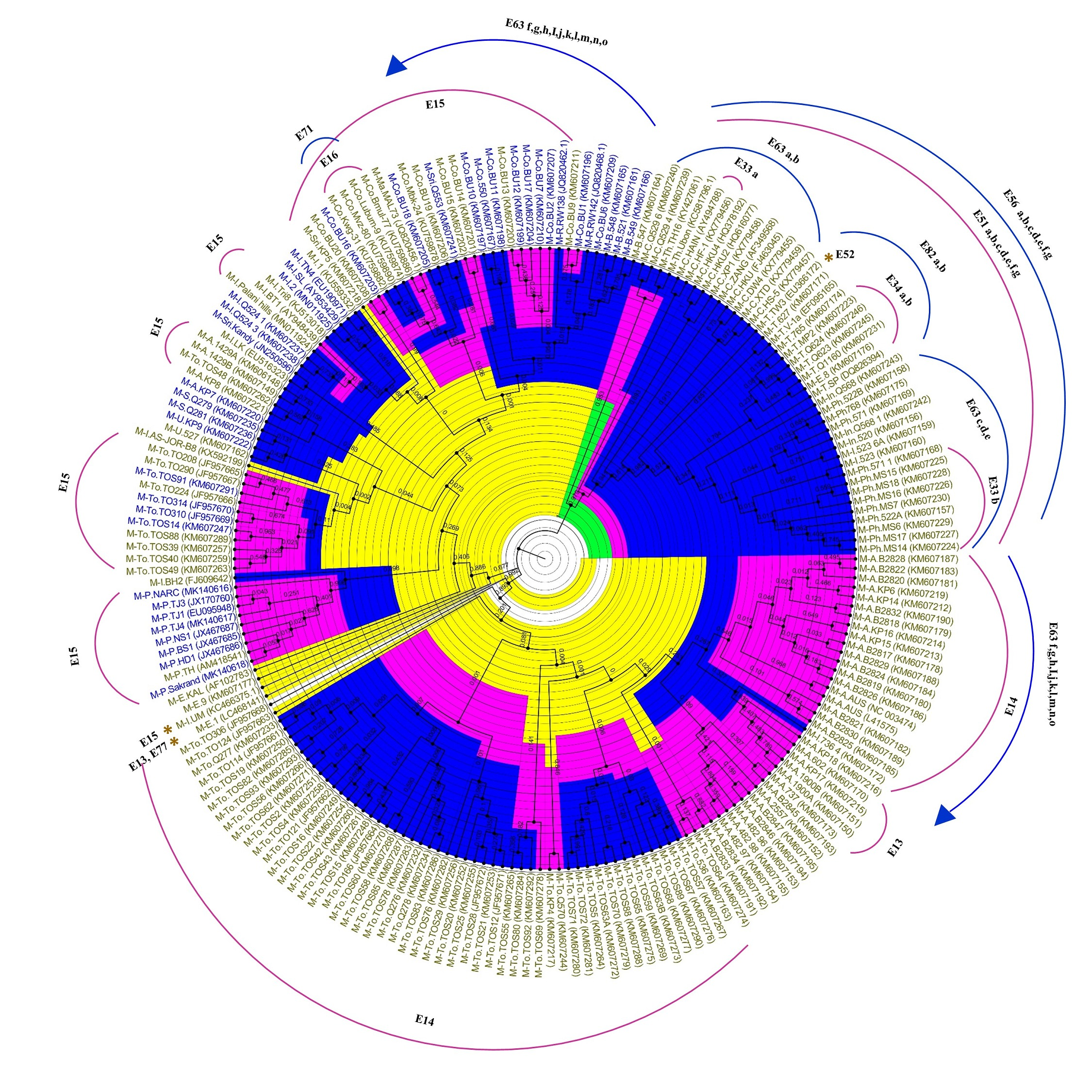

Supplement: S3 Fig — (TIF) [file pone.0263875.s006.tif]

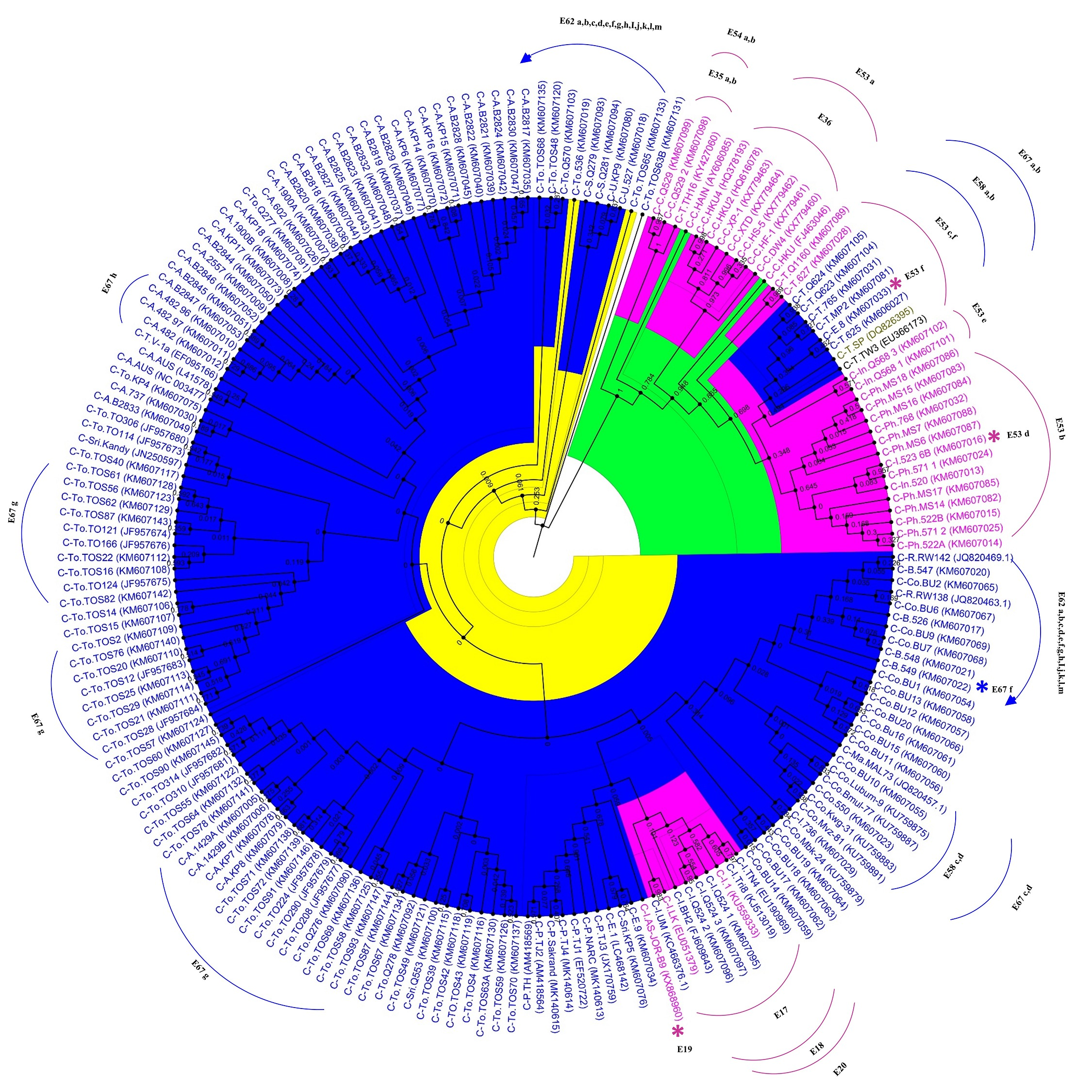

Supplement: S4 Fig — (TIF) [file pone.0263875.s007.tif]

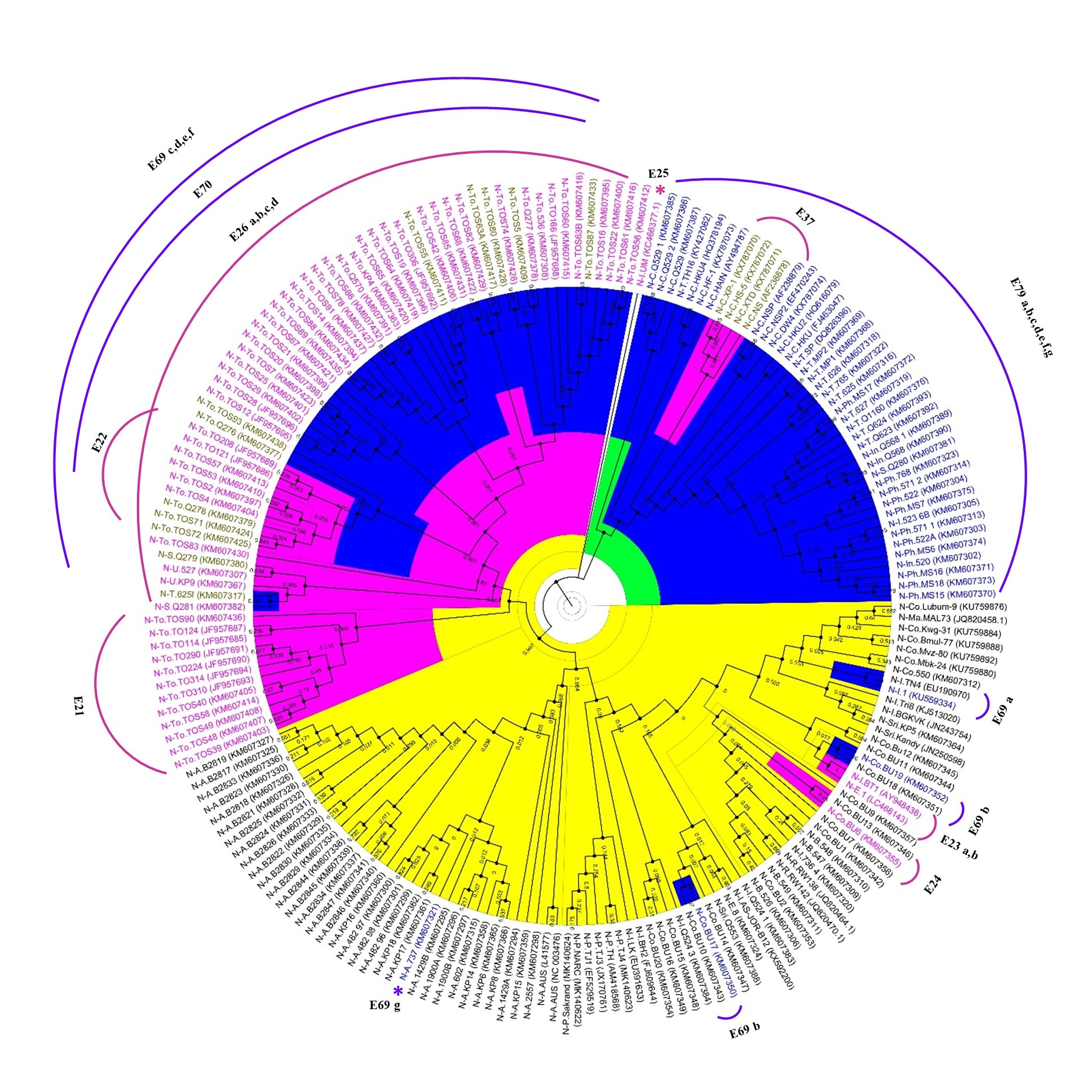

Supplement: S5 Fig — (TIF) [file pone.0263875.s008.tif]
